# Isolation, Co-Crystallization and Structure-Based Characterization of Anabaenopeptins as Highly Potent Inhibitors of Activated Thrombin Activatable Fibrinolysis Inhibitor (TAFIa)

**DOI:** 10.1038/srep32958

**Published:** 2016-09-08

**Authors:** Herman Schreuder, Alexander Liesum, Petra Lönze, Heike Stump, Holger Hoffmann, Matthias Schiell, Michael Kurz, Luigi Toti, Armin Bauer, Christopher Kallus, Christine Klemke-Jahn, Jörg Czech, Dan Kramer, Heike Enke, Timo H. J. Niedermeyer, Vincent Morrison, Vasant Kumar, Mark Brönstrup

**Affiliations:** 1 Sanofi-Aventis Deutschland GmbH, Industriepark Höchst, 65926, Frankfurt am Main, Germany; 2 Cyano Biotech GmbH, Magnusstraße 11, 12489, Berlin, Germany; 3 Interfaculty Institute for Microbiology and Infection Research, Eberhard Karls University Tübingen, Auf der Morgenstelle 28, 72076, Tübingen, Germany; 4 Sanofi, 153 Second Avenue, MA-02451, Waltham, USA; 5 Department of Chemical Biology, Helmholtz Centre for Infection Research, Inhoffenstraße 7, 38124 Braunschweig, Germany

## Abstract

Mature thrombin activatable fibrinolysis inhibitor (TAFIa) is a carboxypeptidase that stabilizes fibrin clots by removing C-terminal arginines and lysines from partially degraded fibrin. Inhibition of TAFIa stimulates the degradation of fibrin clots and may help to prevent thrombosis. Applying a lead finding approach based on literature-mining, we discovered that anabaenopeptins, cyclic peptides produced by cyanobacteria, were potent inhibitors of TAFIa with IC_50_ values as low as 1.5 nM. We describe the isolation and structure elucidation of 20 anabaenopeptins, including 13 novel congeners, as well as their pronounced structure-activity relationships (SAR) with respect to inhibition of TAFIa. Crystal structures of the anabaenopeptins B, C and F bound to the surrogate protease carboxypeptidase B revealed the binding modes of these large (~850 Da) compounds in detail and explained the observed SAR, i.e. the strong dependence of the potency on a basic (Arg, Lys) exocyclic residue that addressed the S1’ binding pocket, and a broad tolerance towards substitutions in the pentacyclic ring that acted as a plug of the active site.

Cardiovascular disease continues to be a major cause of morbidity and death worldwide^1^. A severe risk associated with most cardiovascular diseases, especially during or shortly after heart attack or stroke, is unwanted blood coagulation. It therefore comes as no surprise that anticoagulants belong to the most prescribed drugs. The major classes of drugs include vitamin K antagonists (e.g. warfarin), heparin derivatives (e.g. fondaparinux), platelet inhibitors (e.g. clopidogrel) and direct inhibitors of the coagulation factors thrombin and factor Xa (e.g. dabigatran and rivaroxaban)^2^. However, if overdosed, these drugs carry the risk of causing bleeding, especially in elderly patients with impaired liver function and extensive comedication^3^. As an alternative to the inhibition of blood coagulation, one could also stimulate the breakdown of blood clots, a process called fibrinolysis. The carboxypeptidase TAFIa (activated thrombin activatable fibrinolysis inhibitor) is a central player in fibrinolysis (Supporting Information, Figure S1)^4^,^5^. TAFIa removes carboxy-terminal lysines and arginines from partially degraded fibrin. Because these residues function as docking sites for plasmin and tPA, little plasmin is generated in the absence of these carboxy-terminal lysine and arginine residues, thereby protecting the clot against degradation^6^. Consequently, TAFIa inhibition results in increased plasmin generation and clot degradation, thus showing an antithrombotic effect.

To test the hypothesis that stimulation of fibrinolysis via TAFIa inhibition is associated with a lower risk of bleeding compared to established anticoagulants, we aimed at discovering small molecule inhibitors of TAFIa as novel antithrombotic agents. Purified natural products with elucidated structures were included in this search due to their proven track record as a source of leads and drugs^7^,^8^. A literature mining approach alerted us of the activity of anabaenopeptins against carboxypeptidase A^9^,^10^,^11^,^12^, an enzyme that is closely related to TAFIa. Anabaenopeptins are bioactive peptides, produced by cyanobacteria e.g. during algal blooms^11^. They are cyclic hexapeptides produced by non-ribosomal peptide synthetases (Fig. 1)^13^,^14^,^15^,^16^,^17^. Their chemical scaffold, first described in 1995^18^,^19^, is characterized by a conserved D-lysine residue at position 2 that spans a pentacycle through an isopeptide bond; the pseudo C-terminal residue is linked to the ε-amino function of lysine-2 via an ureido bond. Following the central concept of chemical genetics that similar receptors bind similar ligands^20^, the activity of anabaenopeptins against carboxypeptidase A motivated their test against TAFIa. As reported in a recent paper, a surprisingly potent, single digit nanomolar inhibition of TAFIa by anabaenopeptins was observed, inspiring the structure-based design and synthesis of truncated small molecule analogs^21^.

In the current paper, we explore the ability of natural anabaenopeptin analogues to inhibit TAFIa, based on the isolation of a series of 7 known and 13 hitherto undescribed anabaenopeptins from cyanobacteria. In addition, we obtained crystal structures of the complexes of anabaenopeptin B (**1**), anabaenopeptin C (**2**) and anabaenopeptin F (**3**) with the surrogate protease carboxypeptidase B (CPB). These co-crystal structures revealed the detailed protein-ligand interactions and helped explaining the structure-activity relationships. The results establish anabaenopeptins as a potent hit series for the inhibition of TAFIa and provide the basis for the rational design of related small molecule inhibitors.

## Results

The known anabaenopeptins B, C, and F (**1**, **2** and **3**) were isolated from a culture of the cyanobacterium *Planktothrix rubescens* and tested for their inhibitory activity against TAFIa in an enzymatic assay. Compounds **1**–**3** turned out to be potent inhibitors of TAFIa with IC_50_ values of 1.5, 1.9 and 1.5 nM, respectively^21^. Moreover, the selectivity against other proteases of the coagulation cascade (i.e. FXa, FVIIa, FIIa and FXIa) and against the carboxypeptidases A and N was >500 fold (Supporting Information, Table S1). Previous studies reported activities of anabaenopeptins with basic exocyclic residues against carboxypeptidase A in the low μM range. In a patent application, the inhibition of TAFIa by different marine anabaenopeptin analogues was described with IC_50_ values of 0.1 μM or higher^22^. Thus, while *per se* the activity against TAFIa was predictable, the high level of potency, two orders of magnitudes higher compared to the literature, was unexpected.

### Isolation and Structure Elucidation of Novel Anabaenopeptin Analogues

In order to define the essential structural contributors to activity, a series of 20 anabaenopeptin analogues was isolated from 20 to 200 L cultivations of eight cyanobacterial strains, classified as *Planktothrix* or *Nostoc* sp. As cyanobacteria are photosynthetic autotrophs and therefore can be cultivated in inorganic salt solutions rather than energy-rich cultivation media, the isolation of secondary metabolites is relatively facile due to a low organic media background. Following cell harvest, cell disruption and methanol extraction, all compounds could be obtained in high purity using 1–2 steps of preparative liquid chromatography on reversed phase columns. The yields for the 20 isolated anabaenopeptins ranged between 180 mg and 3.2 mg (Supporting Information). Thus, while the access to the metabolites was technically straightforward, the absolute productivity, expressed as isolated quantity per cultivation volume, remained low (≪1 mg/L).

**Figure 2:**
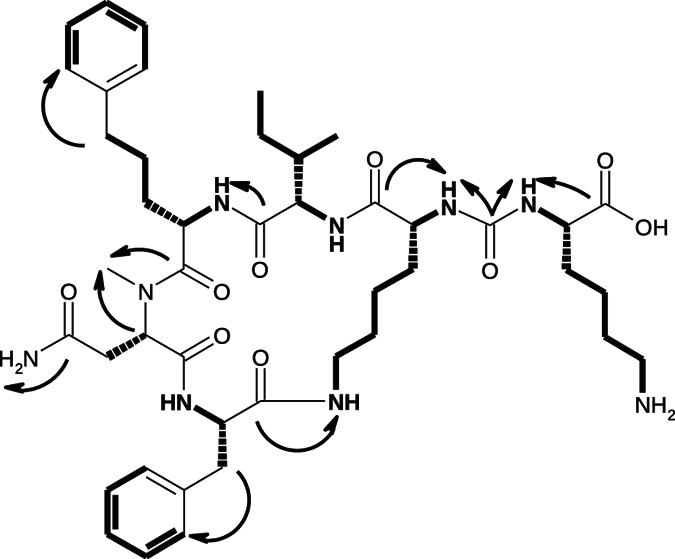
Structure elucidation of 4. Bold bonds indicate spin systems which could be assigned by correlations in the DQF-COSY and TOCSY spectra. Black arrows indicate important correlations in the HMBC spectrum, which were used for the connection of separated spin systems and the sequence assignment.

**Figure 3:**
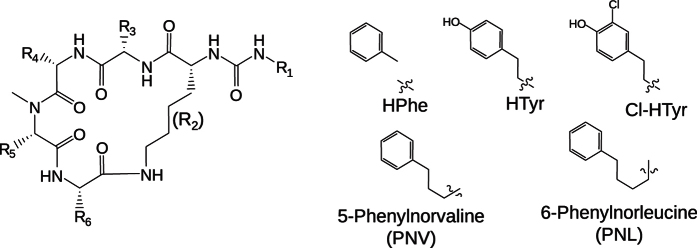
General structure of anabaenopeptins, and structure of residues HPhe, HTyr, PNV, PNL and Cl-HTyr. R1–R6 indicate side chains of amino acids. R2 is a Lys residue in all anabaenopeptins.

The structures of the anabaenopeptins were elucidated by the analyses of 2D-NMR experiments that comprised DQF-COSY, TOCSY, ROESY, HSQC, and HMBC spectra. The correlations that have been used for the identification of single amino acids and for the sequence assignment of analogue **4** are depicted in Fig. 2 as an example. The general structure of all analogues is depicted in Fig. 3, while the corresponding amino acid residues are decoded in Table 1. A complete assignment of proton and carbon chemical shifts is given in the Supporting Information.

While all 20 analogues carry the core anabaenopeptin scaffold described above *per definitionem*, structural diversity is obtained through the side chain decorations. For example, the exocyclic residue R_1_ carried basic (Lys, Arg), aromatic (Phe, Tyr) and unpolar (Ile) amino acids. With hydrophilic (Gly, Ser, Asn), hydrophobic (Ala) and aromatic (HTyr) congeners, a broad variety of residues was incorporated at position R_5_. In contrast, position R_3_ remained conserved and was occupied with Val or Ile. At position R_4_, only aromatic residues were found. In addition to homotyrosine (HTyr), a residue frequently found in anabaenopeptins at R_4_, we discovered that compounds **4**, **5**, **6** and **7** incorporated 5-phenylnorvaline (PNV), compound **8** was featured by 6-phenylnorleucine (PNL), and compound **9** had a 2-chloro-homotyrosine (Cl-HTyr) residue at R_4_. To the best of our knowledge, these residues have not been described in anabaenopeptins before. The closest analogue with an extended alkyl chain to a terminal aromatic residue is pompanopeptin B, isolated from the marine cyanobacterium *Lyngbya confervoides*, that carries an *N*-Me-2-amino-6-(4′-hydroxyphenyl)hexanoic acid (*N*-Me-Alpha) at the R_5_ position^23^.

In the case of **9–12**, (all with *N*-methyl glycine in position R_5_) the analysis of the NMR spectra was complicated by the presence of two sets of signals in a ratio of ca. 1.3:1.0. As indicated by exchange NOEs in the ROESY spectrum, these sets were caused by two different conformations that originated from a different orientation of the amide bond between N-Me-Gly and HTyr/Cl-HTyr in position 4. In the major conformer the orientation was *cis* (strong ROE between *N*-Me-Gly-Hα and HTyr/ClHTyr-Hα); while a *trans* orientation was prevalent in the minor conformer. These compounds have not been described in literature before, but are related to anabaenopeptins NZ825, NZ841 and NZ857^24^. In summary, the cultivation of *Planktothrix* or *Nostoc* sp. led to the known analogues Anabaenopeptin A (**13**)^18^, anabaenopeptin B (**1**)^18^,^25^, anabaenopeptin C (**2**)^26^, anabaenopeptin F (**3**)^27^, oscillamid Y (**14**)^28^ and anabaenopeptins 908 (**16**) and 915 (**15**)^29^, and to 13 analogues that have not been reported before. We have named these new analogues anabaenopeptin SA1 to anabaenopeptin SA13.

### Structure-Activity Relationships of Anabaenopeptins

The activity of all anabaenopeptins against TAFIa was measured in an enzymatic inhibition assay in order to derive structure-activity relationships (Table 1). A key finding was that the strongest determinant of activity was the R_1_ residue. The comparison of **17**, **3** and **14**, which only differ in their R_1_ residues, demonstrated that Lys or Arg side chains were associated with high potency (IC_50_ values of 2.1 and 1.5 nM, respectively), while the Tyr residue led to a decline of activity by two orders of magnitudes (IC_50_ of 400 nM).

An equivalent order was found for **2**, **1** and **13** with IC_50_ values of 1.9 nM, 1.5 nM and 440 nM for the Lys, Arg and Tyr side chains. The two sets of compounds (**17**, **3** and **14** *vs.* **2**, **1** and **13**) only differ in their R_3_ residue, thereby demonstrating that the substitution of Ile by Val at R_3_ has no effect on activity. While analogues with Tyr side chains at the R_1_ positions retained sub-μM potency, an activity drop by another 1–2 orders of magnitude was observed for analogues with Phe or Ile residues at R_1_ (e.g. **9, 10, 11** or **18, 7, 8**). Thus, the potency against TAFIa is strongly determined by the R_1_ residue and drops in the order Arg = Lys ≫ Tyr  > Phe ≈ Ile.

A reverse order of potencies has been observed for carboxypeptidase A, which exhibited a preference for aromatic and aliphatic side chains at R_1_: While anabaenopeptins G, I, J and with Tyr or Ile residues at R_1_ had nanomolar inhibitory concentrations, anabaenopeptin H, having Arg at R_1_, was found to have an IC_50_ of 3.4 μg/ml according to the literature^9^,^10^,^11^.

A pairwise comparison of **1** *vs*. **19**, differing only in their R_5_ residues, demonstrated that the replacement of Ala by Ser was associated with a tenfold loss of activity. A similar trend was observed for **13** vs. **20**, where the replacement of Ala by Ser at R_5_ led to a more than fivefold drop in activity. However, R_5_ is not per se intolerant towards substitutions with polar residues: The pair **17** *vs*. **4** was equipotent in spite of differences in their R_5_ and R_4_ positions (Ala and HTyr vs. Asn and PNV). This finding was confirmed by the pair **3** *vs*. **5** that differed at equivalent positions. Also a switch of aliphatic and aromatic side chains at R_5_ and R_6_ was tolerated in terms of potency, as demonstrated by the two pairs **3** (Phe at R_6_, Ala at R_5_) vs. **16** (Ile at R_6_, HTyr at R_5_) and **14** (Phe at R_6_, Ala at R_5_) vs. **15** (Ile at R_6_, HTyr at R_5_). As the activities for low-potency analogues (with R_1_ = Ile, Phe) were less consistent, we refrain to derive an SAR from their analogues.

In summary, major findings of the study were (i) a strong dependence of activity against TAFIa on basic R_1_ residues (ii) a drop of activity upon replacement of Ala by Ser at position R_5_, and (iii) an otherwise high tolerance towards substitutions in the pentacycle.

### Structural Biology of Anabaenopeptin Complexes

In order to understand the high affinity of anabaenopeptins for TAFIa and the empirically derived structure-activity relationships shown above, the binding modes of three representative analogues (anabaenopeptins B, C and F) in complex with a surrogate protease were determined by X-ray crystallography.

Carboxypeptidase B (CPB) was used instead of TAFIa for practical reasons, since TAFIa was found to be highly unstable with a half-life of approximately 2h at 22 °C^30^. CPB, on the other hand, is stable and highly homologous to TAFIa with 48% sequence identity. Superposition of the first subunits of the crystal structures of bovine proTAFI (pdb code 3dgv)^31^ and a high resolution crystal structure of CPB (pdb code 1z5r)^32^ revealed r.m.s. differences in Cα positions of 0.4 Å for 205 core residues, and 0.26 Å for 22 residues within 8 Å of the catalytic zinc, showing that the proteins and especially the active sites were indeed highly similar.

**Figure 4:**
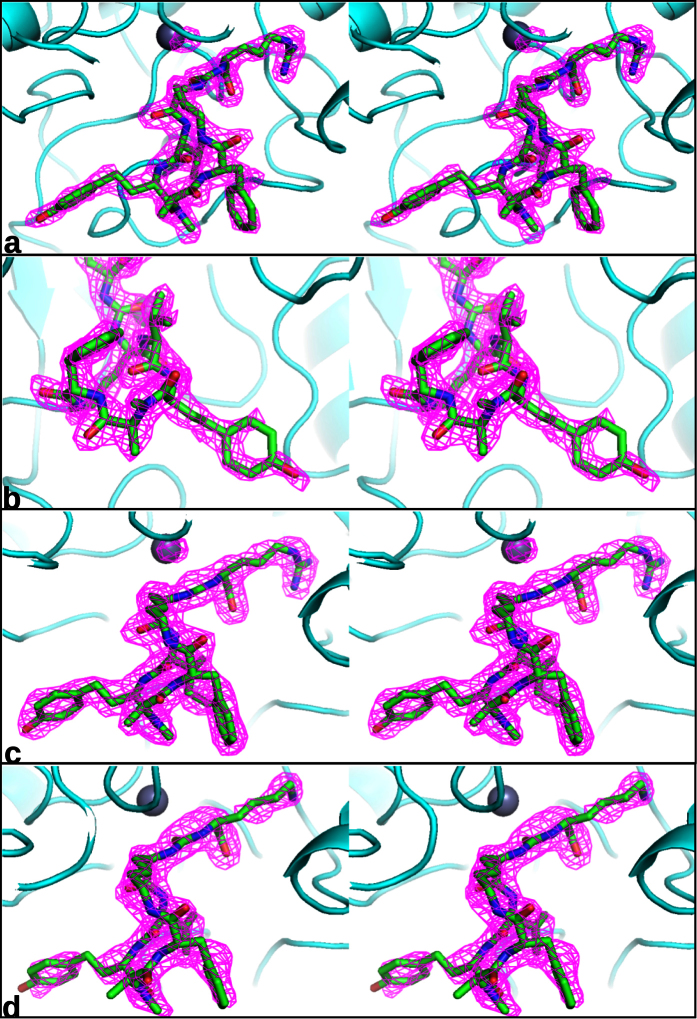
Stereo pictures of omit maps of one of the three independent active sites of P3_2_ carboxypeptidase crystals, soaked with 1 (**a,b**), 3 (**c**), and 2 (**d**). The contour level is 4σ. The resolution is 2.0 Å for the complex with 1, 2.3 Å for the complex with 3 and 2.2 Å for the complex with 2. Figure 2b shows a close-up of the 2.0 Å omit map of the 1–CPB complex contoured at 4σ. The weaker electron density of the homotyrosine residue of anabaenopeptin can be attributed to the flexibility of the side chain since it has few interactions with the protein.

We used CPB crystals grown in the presence of ε-caproic acid with space group P3_2_, since these crystals had wide solvent channels, which allowed the soaking-in of large inhibitors like the anabaenopeptins. Soaking the compounds into CPB crystals with other crystal forms caused the crystals to dissolve, probably because the solvent channels were not wide enough to accommodate these large inhibitors. After soaking, the crystals diffracted to 2.0–2.3 Å, enabling the calculation of detailed and accurate electron density maps (Fig. 4).

### Binding Mode of the Anabaenopeptins

**Figure 5:**
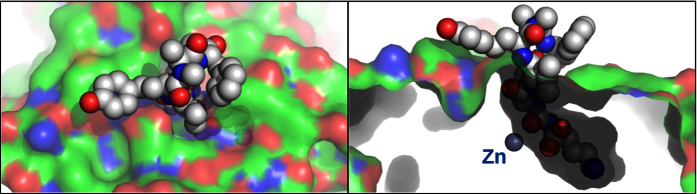
Two views, 90° apart, of the overall binding mode of 2 in carboxypeptidase B. Shown is **2** as a CPK model and carboxypeptidase B as a solvent accessible surface. Also indicated is the catalytic zinc. The anabaenopeptin C molecule fills the active site and access to the active site like a plug.

The general binding modes of **1**–**3** were found to be very similar and in the following discussion the complex with **2** is used as an example. The linear part of the anabaenopeptins, which mimics the carboxy-terminus of the TAFIa substrate fibrin, deeply penetrated the active site channel and bound in the carboxylate- and specificity-pockets, while the circular parts of the anabaenopeptins were positioned in the entrance of the channel and completely blocked the channel like a plug, thereby preventing other molecules from entering it (Fig. 5). Calculations with PISA^33^ revealed that the total surface area of **2** was 1036 Å^3^, while the area of the **2**–CBP interface was 519 Å^3^. Thus, about 50% of the surface of **2** got buried upon binding, contributing to the high affinity.

**Figure 6:**
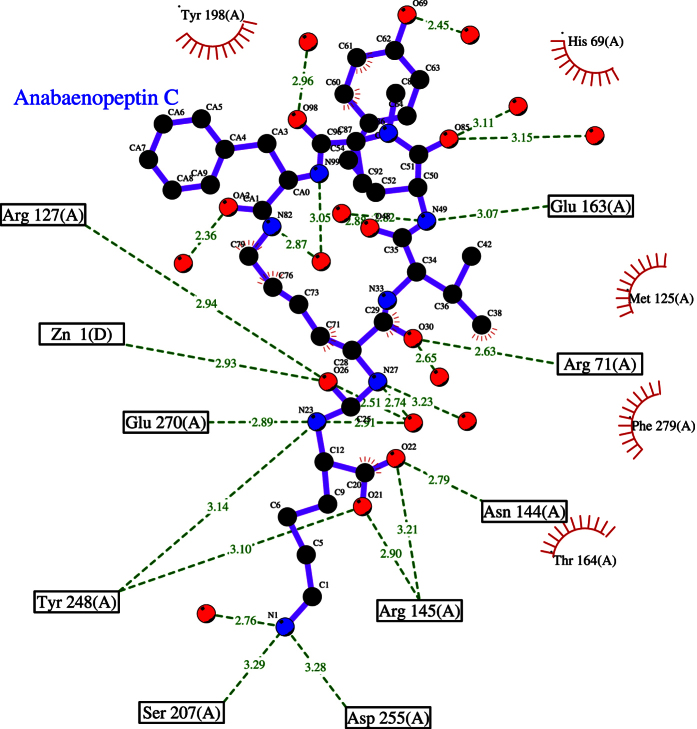
Cartoon of the interactions between 2 and the carboxypeptidase B active site. Arrows indicate potential hydrogen bonds; radiating “suns” indicate hydrophobic (van der Waals) interactions. Multiple interactions are present between the protein and the C-terminal lysine and the urea group of the inhibitor, but few interactions are present at the other side of the inhibitor in the region of the phenylalanine and tyrosine side chains. Not drawn by Ligplot are 3.4 Å interactions of the Tyr248 OH and the urea nitrogens N27 and N33 of **2**.

The Ligplot cartoon^34^ in Fig. 6 depicts the detailed interactions of **2** and CPB. In line with the very high binding affinity of **2**, there were no unsatisfied buried hydrogen bonds. All hydrogen bond donor and acceptor atoms of **2** interacted either with hydrogen bond donor/acceptors from the protein, or with bound water molecules. We found that the C-terminal arginine or lysine side chain (R_1_ in Fig. 3) bound in the S1’ binding pocket and interacted with the side chains of Asp255 and Ser207. The carboxylate group was bound in the acid-binding pocket and interact with the side chains of Asn144, Arg145 and Tyr248. Surprisingly, the urea nitrogens did not interact with the catalytic zinc, but with the hydroxyl group of Tyr248 and the Oε2 of Glu270. The plane of the urea group was approximately perpendicular with respect to the zinc, with the urea oxygen atom located at 2.4 Å distance. Difference electron density maps hint at the presence of a water molecule as additional zinc ligand.

Within the cyclic core of **2**, the side chain of the D-lysine residue (R_2_) had van der Waals interactions with the side chains of Tyr198 and Phe279. The amide moiety bridging R_2_-D-lysine and R_3_-valine interacted with the side chains of Arg71, Arg127 and Tyr248. The amide-nitrogen between the R_3_-valine and R_4_-homotyrosine residues interacted with the side chain of Glu163. Finally, a van der Waals contact was present between the R_4_-homotyrosine side chain of the anabaenopeptin and the side chain of Met125. No further contacts were observed between the ligand and the protein. Especially the R_4_-homotyrosine and R_6_-phenylalanine residues displayed few interactions with the protein. They also appeared to be more flexible than the rest of the molecule, as indicated by higher temperatures and weaker electron densities (Fig. 4b).

Our crystal structures rationalize the structure-activity relationships observed for the R_1_ residue in TAFIa and CPA. The R_1_ residue was bound in the S1’ pocket, which is specific for basic residues like arginine and lysine in TAFIa and CPB, while the S1’ pocket of CPA is specific for large apolar residues like phenylalanine and isoleucine. This difference is caused by the presence of two polar and/or acidic side chains in TAFIa/CPB (Ser207 and Asp255), which are apolar or absent in CPA (Gly207 and Ile255), and an extra apolar side chain (Ile243), which is absent in TAFIa/CPB (Gly243).

## Discussion

The present study describes a lead finding project for inhibitors of TAFIa, a promising target for the induction of fibrinolysis and the prevention of adverse cardiovascular events. A literature mining approach focused on natural products led to the discovery of anabaenopeptins as highly potent inhibitors of TAFIa with inhibitory concentrations in the single-digit nM range. A series of 20 anabaenopeptin analogues were obtained from different cyanobacterial strains. With 13 analogues not reported before, this report describes the largest number of new anabaenopeptins in a single study. The series allowed deriving initial SAR that pointed out the crucial importance of the exocyclic amino acid residue R_1_, but also suggested tolerance for modifications of the pentacyclic core. Both results were confirmed and rationally explained with the help of X-ray co-crystal structures of three anabaenopeptins in the surrogate carboxypeptidase B. Notably; it was possible to soak even large molecules like anabaenopeptins into a carefully selected crystal form of the target molecule. The SAR and the binding mode derived from this study encourage a structural optimization of the macrocyclic part of the molecule through either synthesis, a systematic isolation from cyanobacterial sources, or genetic engineering.

## Methods

### Cultivation, Isolation and Structure Elucidation of Anabaenopeptins

A series of *Planktothrix* and *Nostoc* strains have been used to produce the studied anabaenopeptin congeners: *Planktothrix rubescens* (CBT286) for anabaenopeptin A and oscillamide Y; *Planktothrix rubescens* (CBT287) for **1**, anabaenopeptin F, anabaenopeptin C and **17**; *Nostoc calcicola* (CBT158) for **5**, **7**, and **8**; *Nostoc insulare* (CBT163) for **6**, and **4**; *Planktothrix agardhii* (CBT292) for **15** and **16**; *Planktothrix rubescens* (CBT344) for **19** and **20**; *Nostoc* sp. (CBT599) for **18**; *Nostoc* sp. (ATCC53789) for **9**, **10**, **11** and **12**. The strains were classified on the basis of PCR analysis and sequencing of various marker genes as well as their morphology, and have been deposited in the Cyano Biotech (CBT) culture collection (Cyano Biotech, Berlin, Germany) or the ATTC under the accession numbers indicated above. The strains were cultivated fed-batch in BG11 medium^35^ at 20 °C under continuous light (60–80 μmol m^−2^ s^−1^) in photo bioreactors over a period of several weeks. The total reactor volume was 200 L for the Planktothrix strains and 20 L for the Nostoc strains. The isolation of the anabaenopeptins characterized in this study required individually adapted protocols for cell extraction and purification. Therefore, we don’t provide a generic protocol here, but refer to the Supporting Information, where detailed procedures for the isolation and data for the structural characterization of all 20 anabaenopeptins are provided.

### *In Vitro* Method for the Determination of IC_50’_s of TAFIa

The prepared substances were tested for TAFla inhibition using the Actichrome plasma TAFIa activity kit from American Diagnostica (Pr. No. 874). 29 μL of assay buffer (20 mM Hepes, 150 mM NaCI, pH 7.4) and 10 μL of TAFla (2.5 μg/ml) were added to 1 μL of a 5 mM DMSO solution of the substance and incubated in a 96 half-well microtiter plate at room temperature for 15 minutes. The enzymatic reaction was started by adding 10 μL of TAFla developer (prediluted 1:2 with water). The time course of the reaction was followed at 420 nm in a microtiter plate reader (SpectraMax plus 384; Molecular Devices) for 15 minutes. The IC_50_ was calculated from the averaged values (duplicate determination) of serial dilutions of the substance with the aid of the Grafit 4 software (Erithacus Software, UK).

### Crystallography of Anabaenopeptin Complexes

Carboxypeptidase B from porcine pancreas was purchased from Sigma-Aldrich (Pr. No. C-9584). The lyophilized protein was dissolved in water to 16 mg/ml with 40 mM ε-caproic acid and 1 μl protein solution was equilibrated against 14–20% PEG8000, 100 mM K-Cacodylate, pH 6.5 (anabaenopeptins B and F) or 100 mM MES pH 6.0 (anabaenopeptin C) and 200 mM NaOxalate in a hanging drop setup. Crystals appeared after 2–3 days. To prepare the anabaenopeptin complexes, a CBP crystal was transferred to reservoir solution with 10 mM anabaenopeptin. Crystals were soaked overnight, mounted in a small nylon loop and flash frozen in liquid nitrogen. Data were collected at the European Synchrotron Radiation Facility (ESRF). The crystals diffracted from 2.02 to 2.30 Å resolution. Data processing and scaling were carried out using the XDS package^36^. Model building and inhibitor fitting was done with Quanta (Molecular Simulations, Inc.) and refinement was done with CNX^37^. Figures were produced with Pymol^38^ The final statistics are listed in Table 2. Pictures of the final omit maps are shown in Fig. 4.

The coordinates for the complexes of **1**, **2** and **3** with carboxypeptidase B have been deposited in the Protein Data Bank under the accessions 5LRG, 5LRJ and 5LRK, respectively.

## Additional Information

**How to cite this article**: Schreuder, H. *et al.* Isolation, Co-Crystallization and Structure-Based Characterization of Anabaenopeptins as Highly Potent Inhibitors of Activated Thrombin Activatable Fibrinolysis Inhibitor (TAFIa). *Sci. Rep.* **6**, 32958; doi: 10.1038/srep32958 (2016).

## Supplementary Material

Supplementary Information

## Figures and Tables

**Figure 1:**
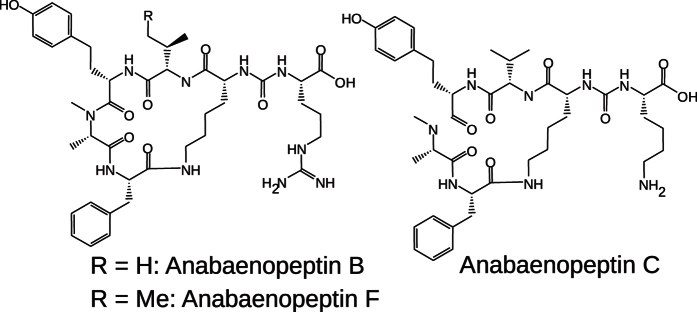
Structural formulae of the anabaenopeptins B, C, and F.

**Table 1:** Composition of anabaenopeptin analogues and their IC50 values against TAFIa.

Cpd	Trivial name	IC_50_ in nM	R_1_	R_3_	R_4_	R_5_	R_6_
**1**	Anabaenopeptin B^18^,^25^	1,5	Arg	Val	HTyr	Ala	Phe
**3**	Anabaenopeptin F^27^	1,5	Arg	Ile	HTyr	Ala	Phe
**16**	Anabaenopeptin 908^29^	1,8	Arg	Val	HTyr	HTyr	Ile
**5**	Anabaenopeptin SA1 (new)	2,2	Arg	Ile	PNV	Asn	Phe
**19**	Anabaenopeptin SA2 (new)	16	Arg	Val	HTyr	Ser	Phe
**2**	Anabaenopeptin C^26^	1,9	Lys	Val	HTyr	Ala	Phe
**17**	Anabaenopeptin SA3 (new)	2,1	Lys	Ile	HTyr	Ala	Phe
**4**	Anabaenopeptin SA4 (new)	3,4	Lys	Ile	PNV	Asn	Phe
**6**	Anabaenopeptin SA5 (new)	790	Ile	Val	PNV	Asn	Phe
**18**	Anabaenopeptin SA6 (new)	51000	Ile	Ile	Hphe	Asn	Phe
**7**	Anabaenopeptin SA7 (new)	13000	Ile	Ile	PNV	Asn	Phe
**8**	Anabaenopeptin SA8 (new)	4800	Ile	Ile	PNL	Asn	Phe
**9**	Anabaenopeptin SA9 (new)	31000	Phe	Ile	Cl-HTyr	Gly	Hphe
**10**	Anabaenopeptin SA10 (new)	7000	Phe	Val	HTyr	Gly	Cl-HTyr
**11**	Anabaenopeptin SA11 (new)	15000	Phe	Ile	HTyr	Gly	Cl-HTyr
**12**	Anabaenopeptin SA12 (new)	4300	Phe	Val	HTyr	Gly	HTyr
**13**	Anabaenopeptin A^18^	440	Tyr	Val	HTyr	Ala	Phe
**14**	Oscillamide Y^28^	400	Tyr	Ile	HTyr	Ala	Phe
**15**	Anabaenopeptine 915^29^	530	Tyr	Val	HTyr	HTyr	Ile
**20**	Anabaenopeptin SA13 (new)	2500	Tyr	Val	HTyr	Ser	Phe

The structure of anabaenopeptins and the composition of R_**1**_–R_**6**_ are given in Fig. 3.

**Table 2:** Data collection and refinement statistics of CPB–inhibitor complexes.

Inhibitor	1	2	3
PDB Code	5LRG	5LRJ	5LRK
Data collection			
Space group	*P3*_*2*_	*P3*_*2*_	*P3*_*2*_
Cell dimensions			
a, b (Å)	124.78	124.69	124.63
c (Å)	48.90	48.12	48.48
Resolution (Å)	54.04–2.02	62.40–2.20	62.24–2.30
	(2.08–2.02)*	(2.27–2.20)*	(2.39–2.30)*
*I/σI*	10.6 (3.4)	12.7 (3.7)	12.8 (4.1)
Observed reflections	160589 (13355)	131294 (11644)	106601 (11392)
*R*_*meas*_ (%)	12.2 (40.7)	9.3 (39.0)	9.8 (34.5)
Completeness (%)	98.1 (97.6)	99.7 (99.5)	99.3 (99.3)
Redundancy	2.9 (2.9)	3.1 (3.1)	2.9 (2.8)
Refinement			
Protein atoms	7308	7308	7308
Inhibitor atoms	180**	174**	183**
water molecules	1756	1457	999
zinc ions	3	3	3
Resolution (Å)	54.0–4–2.02	62.4–2.20	62.2–2.30
*R*_*work*_ (%)	17.6	15.2	15.4
*R*_*free*_ (%)	22.9	21.0	20.0
rmsd bond lengths (Å)	0.009	0.005	0.010
rmsd bond angles (°)	0.77	1.05	0.83

^*^The highest resolution bin is given in brackets.

^**^Three inhibitor molecules.
