# Supplementary material for: Isolation, Co-Crystallization and Structure-Based Characterization of Anabaenopeptins as Highly Potent Inhibitors of Activated Thrombin Activatable Fibrinolysis Inhibitor (TAFIa)
Source: Sci Rep. 2016 Sep 8;6:32958. doi: 10.1038/srep32958 (PMC5015106; doi:10.1038/srep32958)
Supplement: Supplementary Information [file srep32958-s1.docx]

**Supporting Material**

Isolation, Co-Crystallization and Structure-Based Characterization of Anabaenopeptins as Highly Potent Inhibitors of Activated Thrombin Activatable Fibrinolysis Inhibitor (TAFIa)

Herman Schreuder,*^[a]^ Alexander Liesum,^[a]^ Petra Lönze,^[a]^ Heike Stump,^[a]^ Holger Hoffmann,^[a]^ Matthias Schiell,^[a]^ Michael Kurz,^[a]^ Luigi Toti,^[a]^ Armin Bauer,^[a]^ Christopher Kallus,^[a]^ Christine Klemke-Jahn,^[a]^ Jörg Czech,^[a]^ Dan Kramer,^[b]^ Heike Enke,^[b]^ Timo H. J. Niedermeyer,^[b,c]^ Vincent Morrison,^[d]^ Vasant Kumar^[d]^ and Mark Brönstrup*^[a,e]^

^a^ Sanofi-Aventis Deutschland GmbH, Industriepark Höchst, 65926 Frankfurt am Main, Germany ^b^ Cyano Biotech GmbH, Magnusstraße 11, 12489 Berlin, Germany ^c^ present address: Interfaculty Institute for Microbiology and Infection Research, Eberhard Karls University Tübingen, Auf der Morgenstelle 28, 72076 Tübingen, Germany ^d^ Sanofi-Aventis, 153 Second Avenue, Waltham, MA-02451, USA ^e^ present address: Helmholtz Centre for Infection Research, Inhoffenstraße 7, 38124 Braunschweig, Germany.

Table of contents

[Figure S1: Role of TAFI in the fibrinolysis 2](#_Toc402628606)

[Table S1: Activities of Anabaenopeptins against a panel of proteases/carboxypeptidases. 3](#_Toc402628607)

[Structure Elucidation of Anabaenopeptins 3](#_Toc402628608)

[NMR spectroscopy 3](#_Toc402628609)

[Isolation and Structural Data of Anabaenopeptins 4](#_Toc402628610)

[Isolation of Anabaenopeptin A, Anabaenopeptin B, Anabaenopeptin F, Anabaenopeptin C, Oscillamide Y, 17, 20, 19, 5, 7 and 8 4](#_Toc402628611)

[Isolation of 15 and 16 46](#_Toc402628612)

[Isolation of 9, 10, 11 and 12 56](#_Toc402628613)

[Isolation of 4 and 6 75](#_Toc402628614)

[Isolation of 18 84](#_Toc402628615)

Figure S1: Role of TAFI in the fibrinolysis. Thrombin, generated by the blood coagulation cascade not only cleaves fibrinogen to generate insoluble fibrin which contributes to the building of a blood clot, it also cleaves proTAFI, generating active TAFIa. TAFIa removes C-terminal lysines from partially degraded fibrin. Fibrin-lys functions as a cofactor for the activation of plasminogen by tPA to the fibrin-degrading plasmin. Without C-terminal lysines present on fibrin, very little plasmin is generated and very little fibrin is degraded, resulting in a stable clot. Inhibiting TAFIa leads to increased plasmin generation and clot dissolution, which could prevent thrombosis. Abbreviations: TAFI, thrombin activatable fibrinolysis inhibitor; TAFIa, activated TAFI; tPA, tissue plasminogen activator, generates active plasmin from plasminogen, leading to clot dissolution. Currently the only approved therapy for stroke.

### **Table S1: Activities of Anabaenopeptins against a panel of proteases/carboxypeptidases**.

|  | CPA | CPN | FXa | FVIIa | FIIa | FXIa |
| --- | --- | --- | --- | --- | --- | --- |
|  | IC_50_ (µM) / Inh.@100µM in % | | Ki (µM)/ Inh.@100µM in % | | | |
| Anabaenopeptin B | 3.9 | 5.4 | 22% | 35% | 1.6 | 24 |
| Anabaenopeptin F | 1.1 | 2.7 | 38 | 35 | 0.35 | 3.9 |
| Anabaenopeptin C | 31% | 5.7 | 42% | 36% | 6.0 | 12.0 |

## Structure Elucidation of Anabaenopeptins

### NMR spectroscopy

^1^H-1D- and 2D-NMR spectra were recorded on either a Bruker AVANCE 500 spectrometer operating at a proton frequency of 500.30 MHz and a ^13^C-carbon frequency of 125.82 MHz or on a Bruker AVANCE 700 spectrometer operating at a proton frequency of 700.20 MHz and a ^13^C-carbon frequency of 176.08 MHz. Both instruments were equipped with a 5 mm TXI cryo probe heads. The 1D ^13^C-spectra were recorded on a Bruker DRX 600 operating at a proton frequency of 600.20 MHz and a ^13^C-carbon frequency of 150.94 MHz. This instrument was equipped with a room temperature ^13^C-selective probe head. All experiments were carried out with samples of 3 - 5 mg compound dissolved in 600 μl d6-DMSO at 300 K. For structure elucidation and complete assignment of proton and carbon resonances 1D-^1^H, 1D-^13^C, DQF-COSY, ROESY (mixing time 150 ms, spinlock field 2 kHz), HSQC, and HMBC spectra were acquired. ^1^H- and ^13^C-chemical shifts were referenced to the solvent signals (^1^H: 2.50 ppm, ^13^C: 39.50 ppm).

Two-dimensional homonuclear experiments, DQF-COSY and ROESY, were performed with a spectral width of 10 ppm. Spectra were recorded with 512 increments in t_1_ and 4096 complex data points in t_2_. For each t_1_ value 2 (DQF-COSY) or 8 (ROESY) transients were averaged, respectively.

For HSQC spectra 512 increments with 2048 complex data points in t_2_ were collected using a sweep width of 10 ppm in the proton and 160 ppm in the carbon dimension. For each t_1_ value 4 transients were averaged. The HMBC spectrum was acquired with a sweep width of 10 ppm in the proton and 200 ppm in the carbon dimension using a defocusing delay of 62 ms (optimized for coupling constants of 8 Hz). A total of 16 transients were averaged for each of 512 increments in t_1_, and 4096 complex points in t_2_ were recorded.

## Isolation and Structural Data of Anabaenopeptins

Isolation of Anabaenopeptin A, Anabaenopeptin B, Anabaenopeptin F, Anabaenopeptin C, Oscillamide Y, 17, 20, 19, 5, 7 and 8

**Cell disruption and extraction (CBT287, CBT286, CBT344, CBT158)**

After completion of a 200 L-fermentation, the culture broth was filtered and the remaining cells were freeze dried. The lyophilized material was transferred into a 2 L Schott bottle and suspended with 2 L MeOH/H_2_O (ratio 1:1). The suspension was dispersed for 5 min using an ultra turrax and then transferred into a french press. Cell disruption has been performed for 30 min at a pressure of 2250 bar. The extract (~2 L) was centrifuged for 30 min using a Heraeus cryofuge 8500 (4 °C, 5000 rpm). After 30 min the supernatant was decanted and the cell pellet was re-suspended in an ultrasonic bath with 2 L MeOH/H_2_O (ratio 1:1) for 30 min. Extract 2 was also centrifuged for 30 min at 5000 rpm. The supernatants were combined and filtered to give ~4 L crude exctract.

**Solid phase extraction**

The crude extract (ca. 4 L) has been loaded onto a column (dimension: 160 x 200 mm) filled with ~3.0 L of CHP-20P (MCI® Gel, 75-150µ, Mitsubishi Chemical Corporation) material. For strains CBT287 and CBT344 a gradient with 2-propanol : ammonium acetate pH 7.0 was applied whereas for strain CBT158 a gradient with acetonitrile : water was applied (10 % to 100 % within 40 min, flow rate: 240 ml/min). Fractions have been collected every 4 min over a period of 40 min. Fractions containing the compounds of interest were freeze-dried and further purified.

**Isolation of Anabaenopeptin A**

10g of the dried biomass was stirred for 60 min in 2.5 l of a methanol:water (50:50) mixture. The solution was filtered, and the filtrate was put on an SPE column (MCI, CHP20P material, 50mm x 200 mm). A solid phase extraction using a NH4OAc (pH 4.6) : acetonitrile gradient (95:5 at 0 min to 0:100 at 20 min to 0:100 at 30 min) was performed applying a 90 ml/min flow rate and a fraction volume of 50 ml. Fractions 9-15 contained the compounds of interest and were therefore combined and freeze-dried, yielding 1.9 g of raw material. The raw material was dissolved in 40 ml DMSO and further purified by reversed-phase chromatography. The separation was performed on a Waters® X-Bridge RP-18 5µm (30 mm x 100 mm) using a 0,3% formic acid : acetonitrile gradient (95:5 at 0 min to 5:95 at 20 min to 5:95 at 30 min) applying a 50 ml/min flow rate. The fractions were analyzed and collected into 13 pools. Pool 6 contained the target compound and was freeze-dried. The freeze-dried material was then dissolved in 1.5 ml DMF and further purified by reversed-phase chromatography. The separation was performed on a Waters® X-Bridge RP-18 5µm (19 mm x 100 mm) using a 0,3% formic acid : acetonitrile gradient (95:5 at 0 min to 5:95 at 20 min to 5:95 at 30 min) applying a 75 ml/min flow rate and a fraction collection triggered by the UV signal intensity at 220 nm. After freeze-drying of fractions, high purity material of anabaenopeptin A (32 mg).

*Anabaenopeptin A* (13)

UV: 204, 278 nm

Molecular formula: C44H57N7O10, Monoisotopic molecular mass (calc.): 843.4167 Da

calc. [M-H]^-^: 842.4089 Da; observed [M-H]^-^: 842.4073 Da

Fig. Structure of *Anabaenopeptin A* (13)


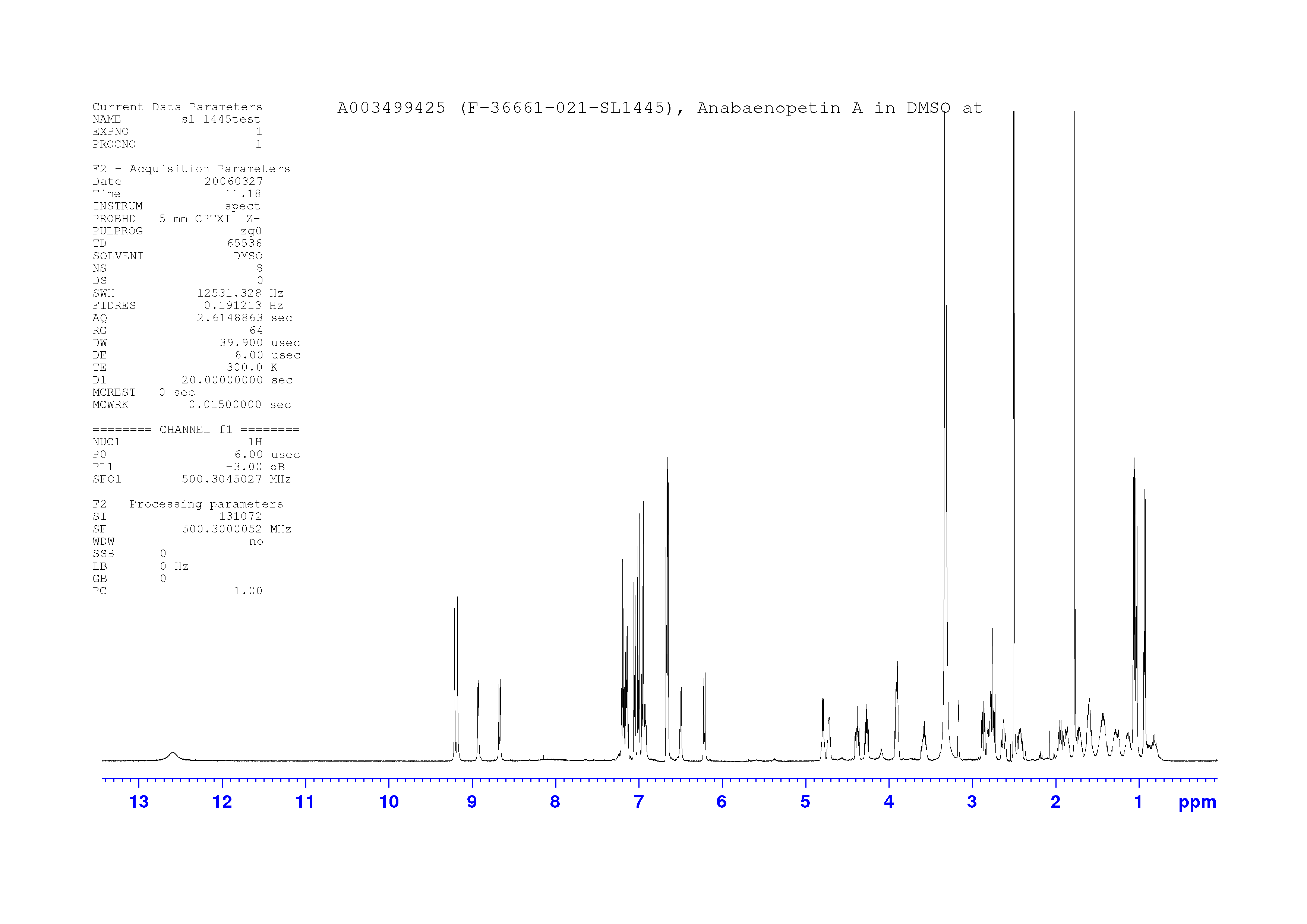
Fig. : ^1^H-spectrum of *Anabaenopeptin A* in DMSO at 300 K.

^1^H-NMR (DMSO-d_6_, 500MHz) δ 0.81 (m,1H), 0.93 (d, 3H), 1.03 (d, 3H), 1.06 (d, 3H), 1.13 (m, 1H), 1.44 (m, 2H), 1.60 (m, 2H), 1.72 (m, 1H), 1.77 (s, 3H), 1.87 (m, 1H), 1.94 (m, 1H), 2.43 (m, 1H), 2.63 (m, 1H), 2.83-2.71 (m, 3H), 3.58 (m, 1H), 3.91 (m, 2H), 4.27 (m, 1H), 4.38 (m, 1H), 4.72 (m, 1H), 4.79 (m, 1H), 6.21 (d, 1H), 6.50 (1H, d), 6.66 (m, 4 H), 6.92 (d, 1H), 6.95 (d, 2H), 7.01 (d, 2H), 7.21-7.12 (m, 4 H), 8.67 (d, 1H), 8.93 (d, 1H), 9.18 (s, 1H), 9.21 (s, 1H), 12.59 (s, 1H).

**Isolation of Anabaenopeptin B and Anabaenopeptin F**

Fractions 5-9 from solid phase extraction of CBT287 were separately purified using a Waters Dynamax Pursuit C18 column (dimension: 41 mm x 100 mm, 10 µm) with a Waters XTerra® pre-column (dimension: 19 x 10 mm, 10 µm). Compounds were eluted using a gradient of acetonitrile : ammonium acetate buffer pH 7.0 (flow rate: 150 ml/min, 5 to 95 % within 43 min). The eluents have been collected in 50 ml-fractions using UV-triggering. The purification of fraction 6 gave highly pure material of anabaenopeptin B (180 mg) and anabaenopeptin F (86 mg). Fractions containing other anabaenopeptins were combined and freeze dried.

*Anabaenopeptin B* (**1**)

UV: 206, 276 nm

C41H60N10O9, Monoisotopic molecular mass (calc.): 836.4545 Da

Calc. [M+H^+^]: 837.4623 Da; observed [M+H^+^]: 837.4633 Da

Fig. Structure of *Anabaenopeptin B*

**
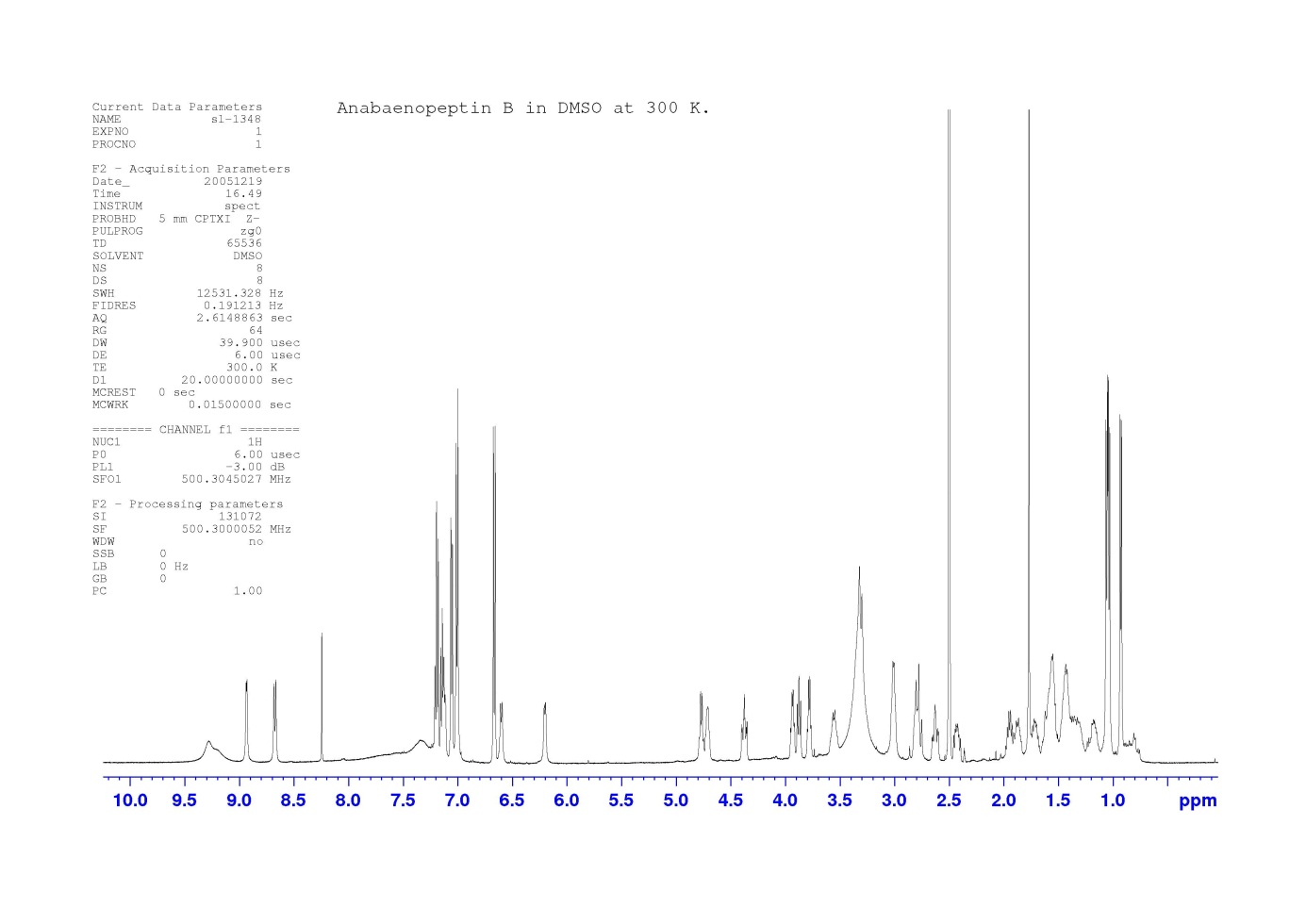
**

Fig. : ^1^H-spectrum of *Anabaenopeptin B* in DMSO at 300 K.


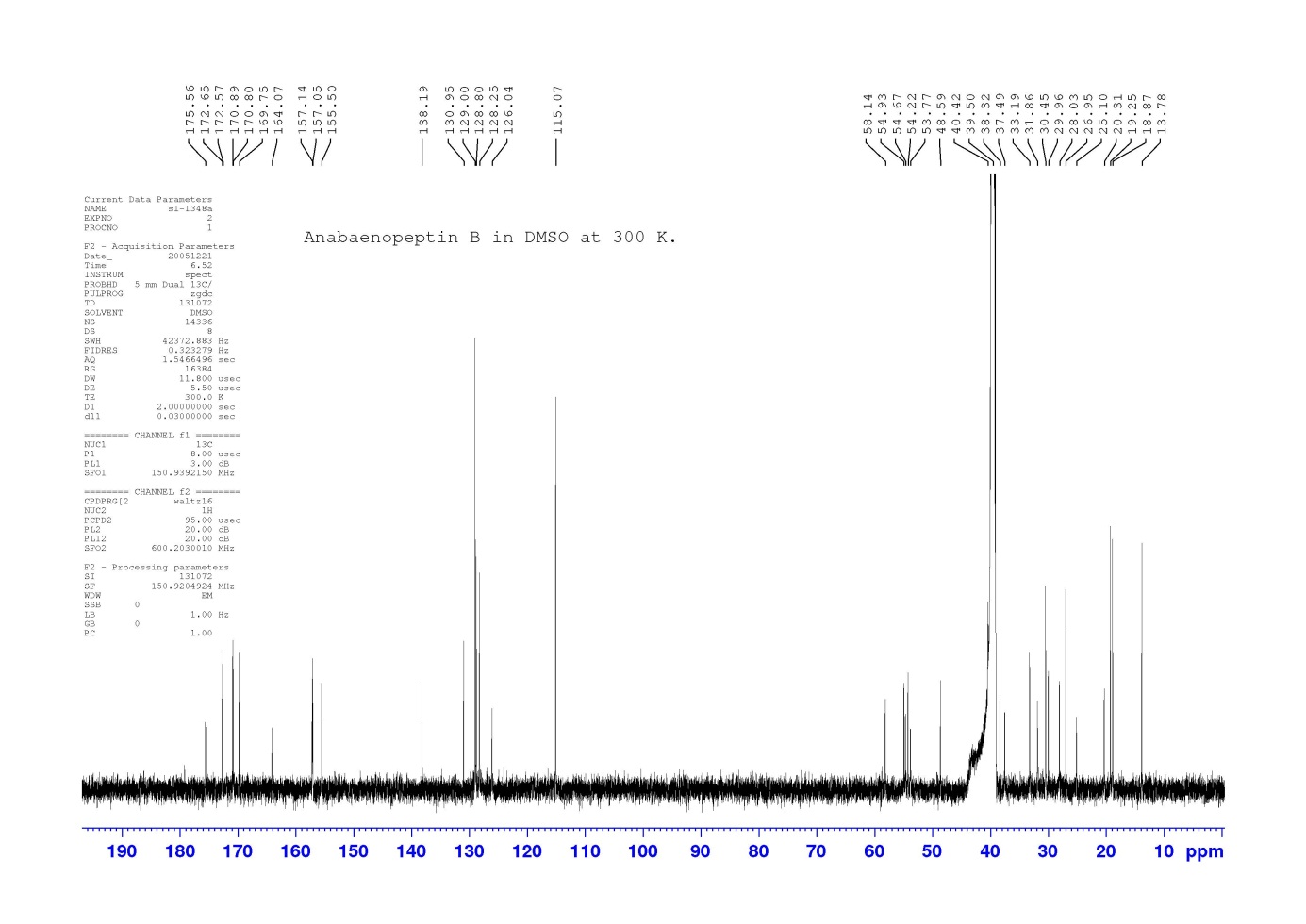


Fig. : ^13^C-spectrum of *Anabaenopeptin B* in DMSO at 300 K.

Table : Chemical shifts of *Anabaenopeptin B* in DMSO at 300 K.

|  | ^1^H | ^13^C |
| --- | --- | --- |
| Phe-1 NH | 8.67 | - |
| α | 4.38 | 54.93 |
| β | 3.31/2.78 | 37.49 |
| γ | - | 138.19 |
| δ | 7.06 | 128.80 |
| ε | 7.19 | 128.25 |
| ζ | 7.14 | 126.04 |
| C’ | - | 170.80 |
| N-Me-Ala-2 NMe | 1.77 | 26.95 |
| α | 4.77 | 54.22 |
| β | 1.06 | 13.78 |
| C’ | - | 169.75 |
| HTy-3 NH | 8.93 | - |
| α | 4.72 | 48.59 |
| β | 1.88/1.71 | 33.19 |
| homo-β | 2.63/2.43 | 30.45 |
| γ | - | 130.95 |
| δ | 7.01 | 129.00 |
| ε | 6.67 | 115.07 |
| ζ | - | 155.50 |
| ζ-OH | broad | - |
| C’ | - | 170.89 |
| Val-4 NH | 7.01 | - |
| α | 3.88 | 58.14 |
| β | 1.95 | 29.96 |
| γ | 1.04 | 18.87 |
| γ’ | 0.93 | 19.25 |
| C’ | - | 172.65 |
| Lys-5 NH | 6.60 | - |
| α | 3.93 | 54.67 |
| β | 1.60 | 31.86 |
| γ | 1.31/1.17 | 20.31 |
| δ | 1.44 | 28.03 |
| ε | 3.56/2.81 | 38.32 |
| ζ-NH | 7.13 | - |
| C’ | - | 172.57 |
| Arg-6 NH | 6.20 | - |
| α | 3.78 | 53.77 |
| β | 1.56 | 30.45 |
| γ | 1.43/1.36 | 25.10 |
| δ | 3.01 | 40.42 |
| ε | broad | - |
| ζ | - | 157.14 |
| ζ-NH_2_ | broad | - |
| C’ | - | 175.56 |
| 1’ | - | 157.05 |

*Anabaenopeptin F* (**3**)

UV: 208, 278 nm

C42H62N10O9, Monoisotopic molecular mass (calc.): 850.47012 Da

Calc. [M+H^+^]: 851.4780 Da; observed [M+H^+^]: 851.4803 Da

Fig. Structure of *Anabaenopeptin F*

**
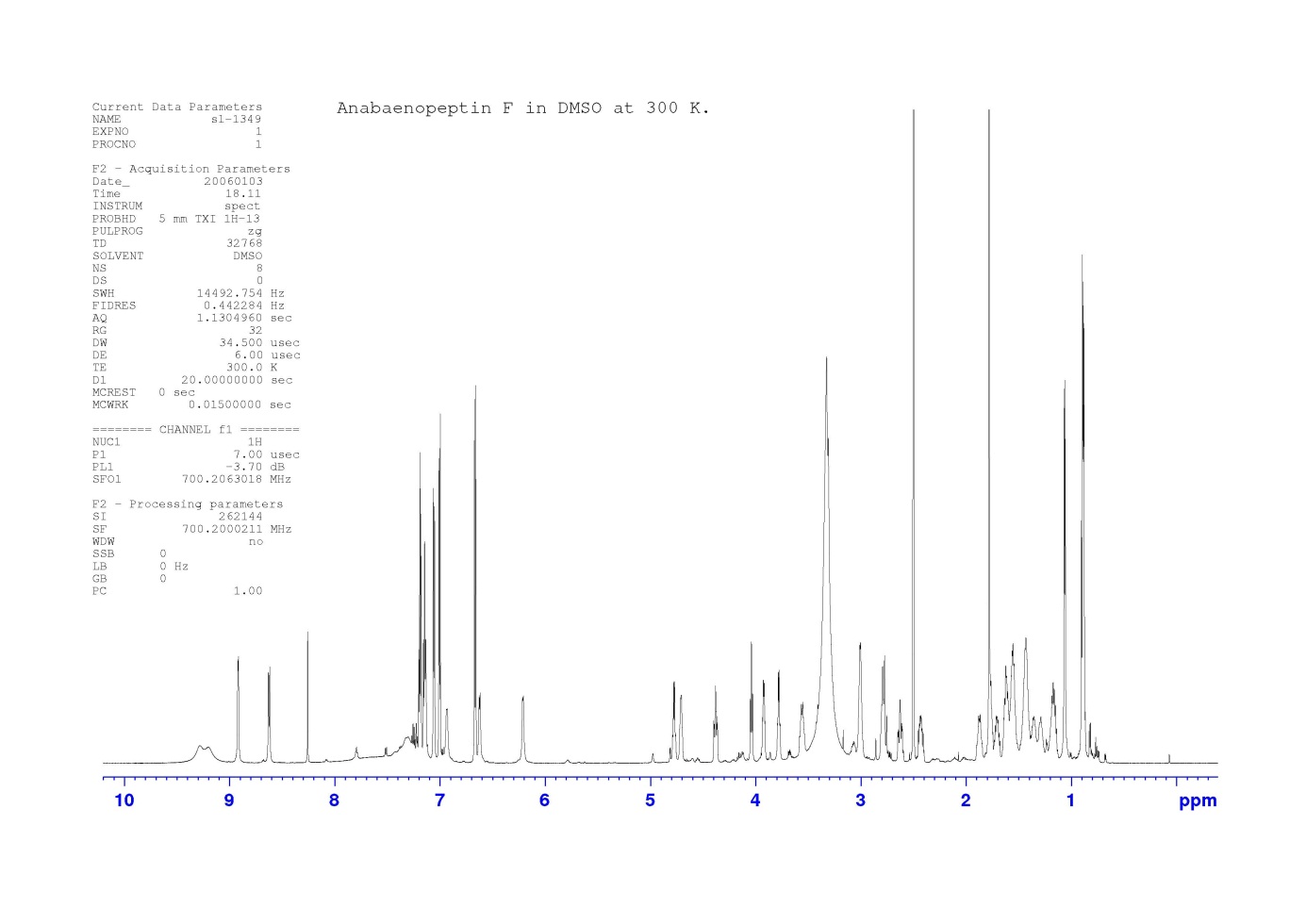
**

Fig. : ^1^H-spectrum of *Anabaenopeptin F* in DMSO at 300 K.


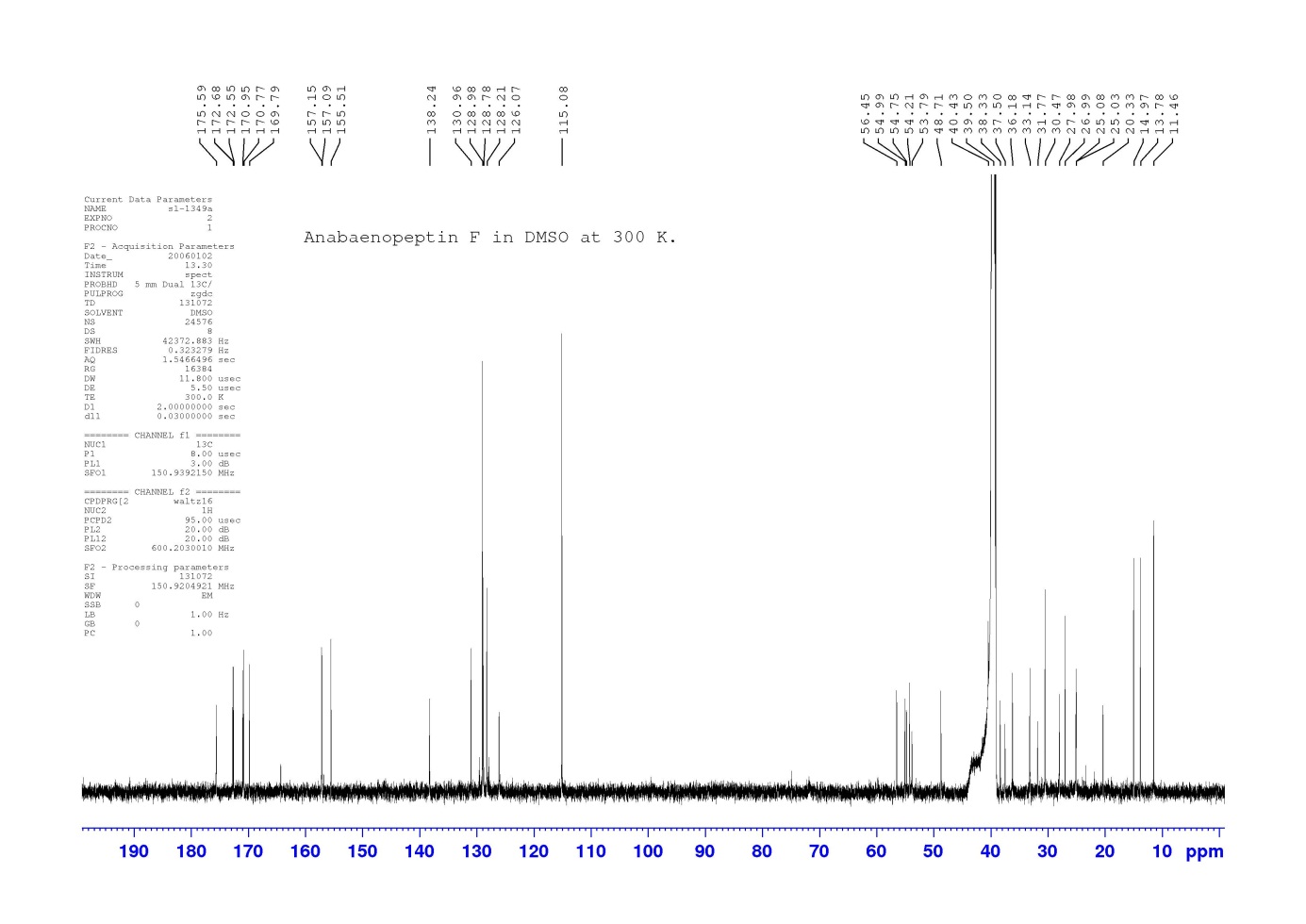


Fig. : ^13^C-spectrum of *Anabaenopeptin F* in DMSO at 300 K.

Table : Chemical shifts of *Anabaenopeptin F* in DMSO at 300 K.

|  | ^1^H | ^13^C |
| --- | --- | --- |
| Phe-1 NH | 8.62 | - |
| α | 4.38 | 54.99 |
| β | 3.31/2.77 | 37.50 |
| γ | - | 138.24 |
| δ | 7.06 | 128.78 |
| ε | 7.19 | 128.21 |
| ζ | 7.15 | 126.07 |
| C’ | - | 170.77 |
| N-Me-Ala-2 NMe | 1.78 | 26.99 |
| α | 4.77 | 54.21 |
| β | 1.06 | 13.78 |
| C’ | - | 169.79 |
| HTy-3 NH | 8.92 | - |
| α | 4.71 | 48.71 |
| β | 1.87/1.71 | 33.14 |
| homo-β | 2.63/2.43 | 30.47 |
| γ | - | 130.96 |
| δ | 7.00 | 128.98 |
| ε | 6.67 | 115.08 |
| ζ | - | 155.51 |
| ζ-OH | broad | - |
| C’ | - | 170.95 |
| Ile-4 NH | 6.93 | - |
| α | 4.04 | 56.45 |
| β | 1.76 | 36.18 |
| β-Me | 0.88 | 14.97 |
| γ | 1.62/1.16 | 25.03 |
| δ | 0.89 | 11.46 |
| C’ | - | 172.68 |
| Lys-5 NH | 6.62 | - |
| α | 3.92 | 54.75 |
| β | 1.61/1.56 | 31.77 |
| γ | 1.29/1.18 | 20.33 |
| δ | 1.44 | 27.98 |
| ε | 3.56/2.79 | 38.33 |
| ζ-NH | 7.14 | - |
| C’ | - | 172.55 |
| Arg-6 NH | 6.21 | - |
| α | 3.78 | 53.79 |
| β | 1.55 | 30.47 |
| γ | 1.43/1.36 | 25.08 |
| δ | 3.01 | 40.43 |
| ε | Broad | - |
| ζ | - | 157.15 |
| ζ-NH_2_ | broad | - |
| C’ | - | 175.59 |
| 1’ | - | 157.09 |

**Isolation of Oscillamide Y**

The separation was performed on a Waters® Sunfire RP-18 5µm (30 mm x 100 mm) using a 0,3% formic acid : acetonitrile gradient (95:5 at 0 min to 5:95 at 20 min to 5:95 at 30 min) applying a 50 ml/min flow rate and a fraction collection triggered by the UV signal intensity at 220 nm. After freeze-drying of fractions, high purity material of anabaenopeptin B (66 mg), anabaenopeptin F (45 mg) and oscillamide Y (24 mg) was obtained.

**14**, *Oscillamide Y*

UV: 203, 278 nm

C45H59N7O10, Monoisotopic molecular mass (calc.): 857.4323 Da

Calc. [M-H^+^]: 856.4245 Da; Observed [M-H^+^]: 856.4248 Da

Fig. 1 Structure of **14**, *Oscillamide Y*


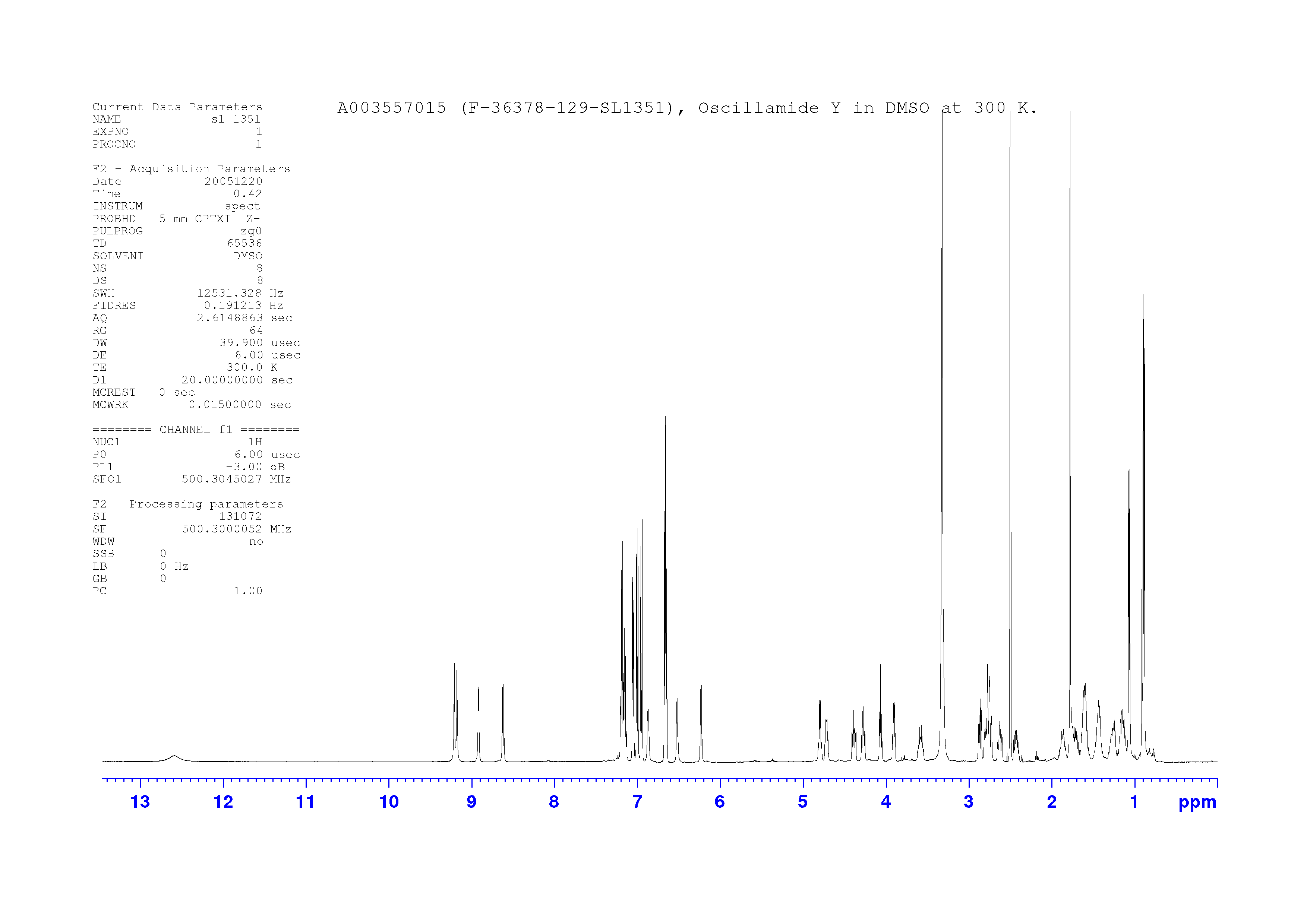


Fig. : ^1^H-spectrum of **14**, *Oscillamide Y* in DMSO at 300K.


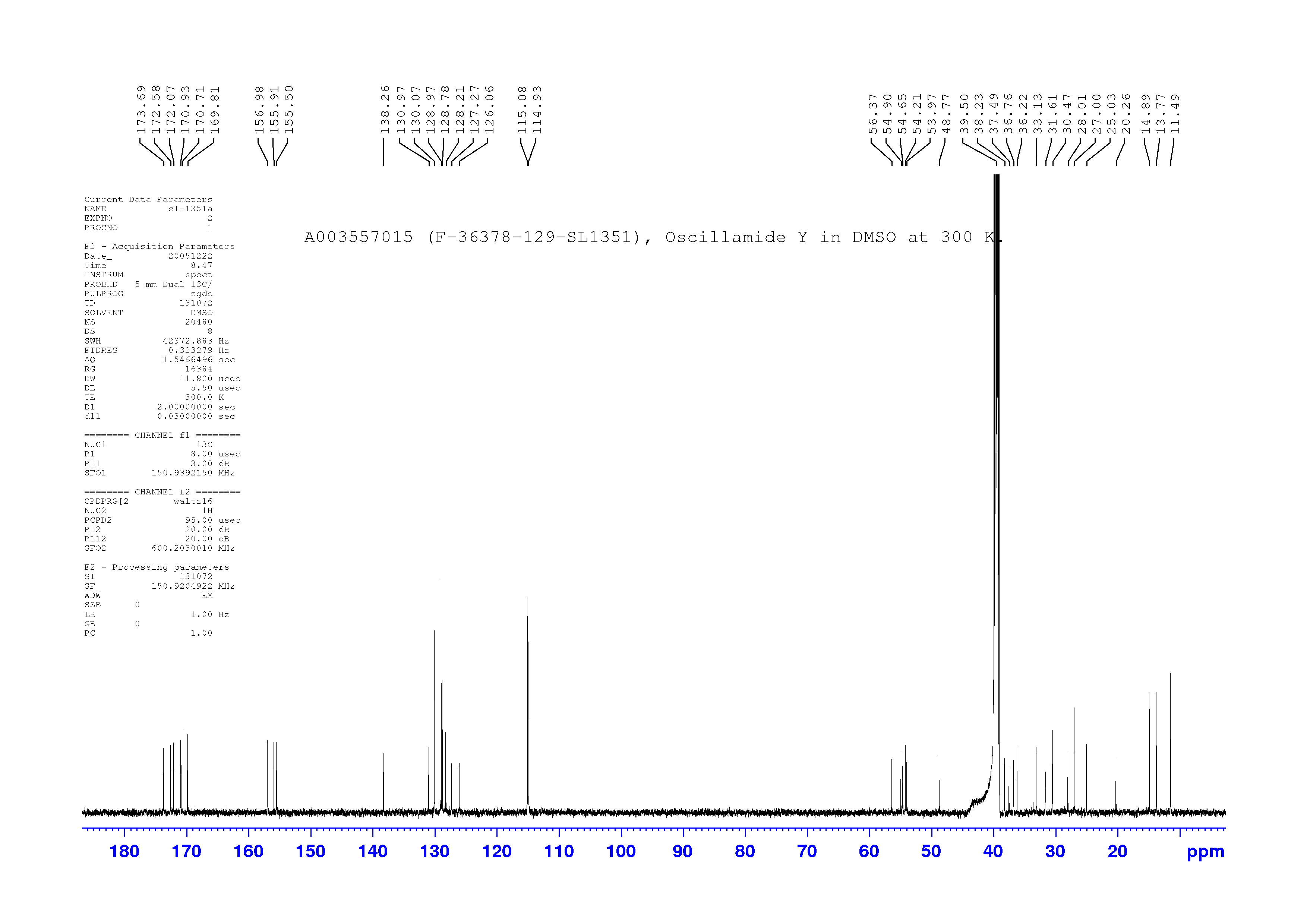


Fig. : ^13^C-spectrum of **14**, *Oscillamide Y* in DMSO at 300K.

Table 1: Chemical shifts of **14**, *Oscillamide Y* in DMSO at 300 K.

|  | ^1^H | ^13^C |
| --- | --- | --- |
| Phe-1 NH | 8.62 | - |
| α | 4.38 | 54.90 |
| β | 3.32/2.74 | 37.49 |
| γ | - | 138.26 |
| δ | 7.05 | 128.78 |
| ε | 7.19 | 128.21 |
| ζ | 7.15 | 126.06 |
| C’ | - | 170.71 |
| N-Me-Ala-2 NMe | 1.77 | 27.00 |
| α | 4.79 | 54.21 |
| β | 1.06 | 13.77 |
| C’ | - | 169.81 |
| HTy-3 NH | 8.92 | - |
| α | 4.71 | 48.77 |
| β | 1.87/1.70 | 33.13 |
| homo-β | 2.62/2.43 | 30.48 |
| γ | - | 130.97 |
| δ | 7.00 | 128.97 |
| ε | 6.66 | 115.08 |
| ζ | - | 155.50 |
| ζ-OH | 9.18 | - |
| C’ | - | 170.94 |
| Ile-4 NH | 6.87 | - |
| α | 4.06 | 56.37 |
| β | 1.76 | 36.22 |
| β-Me | 0.88 | 14.89 |
| γ | 1.61/1.15 | 25.03 |
| δ | 0.89 | 11.49 |
| C’ | - | 172.58 |
| Lys-5 NH | 6.52 | - |
| α | 3.90 | 54.65 |
| β | 1.60 | 31.61 |
| γ | 1.26/1.14 | 20.26 |
| δ | 1.48 | 28.01 |
| ε | 3.58/2.79 | 38.24 |
| ζ-NH | 7.16 | - |
| C’ | - | 172.07 |
| Tyr-6 NH | 6.23 | - |
| α | 4.27 | 53.97 |
| β | 2.86/2.75 | 36.76 |
| γ | - | 127.27 |
| δ | 6.95 | 130.07 |
| ε | 6.65 | 114.93 |
| ζ | - | 155.91 |
| ζ-OH | 9.21 | - |
| C’ | - | 173.69 |
| 1’ | - | 156.98 |

**Isolation of anabaenopeptin C and 17**

The pre-purified pool of residual fractions from the HPLC run that led to the isolation of anabaenopeptin B and anabaenopeptin F was directly loaded onto a Waters Sunfire C18 column (dimension: 20 mm x 100 mm, 5 µm) with a Waters XTerra® pre-column (dimension: 19 x 10 mm, 10 µm). Compounds were eluted with a gradient of acetonitrile : ammonium acetate buffer pH 7.0 (flow rate: 45 ml/min, 5 to 40 % at 40 min, 40 to 95 % at 43 min). The eluents have been collected in 1 ml-fractions using UV-triggering. After freeze-drying, pure material of anabaenopeptin C (11 mg) and **17** (16 mg) was obtained.

*Anabaenopeptin C* (**2**)

UV: 206, 276 nm

C41H60N8O9, Monoisotopic molecular mass (calc.): 808.44833 Da

Calc. [M+H^+^]: 809.4561 Da; Observed [M+H^+^]: 809.4580 Da

Fig. Structure of *Anabaenopeptin C*

**
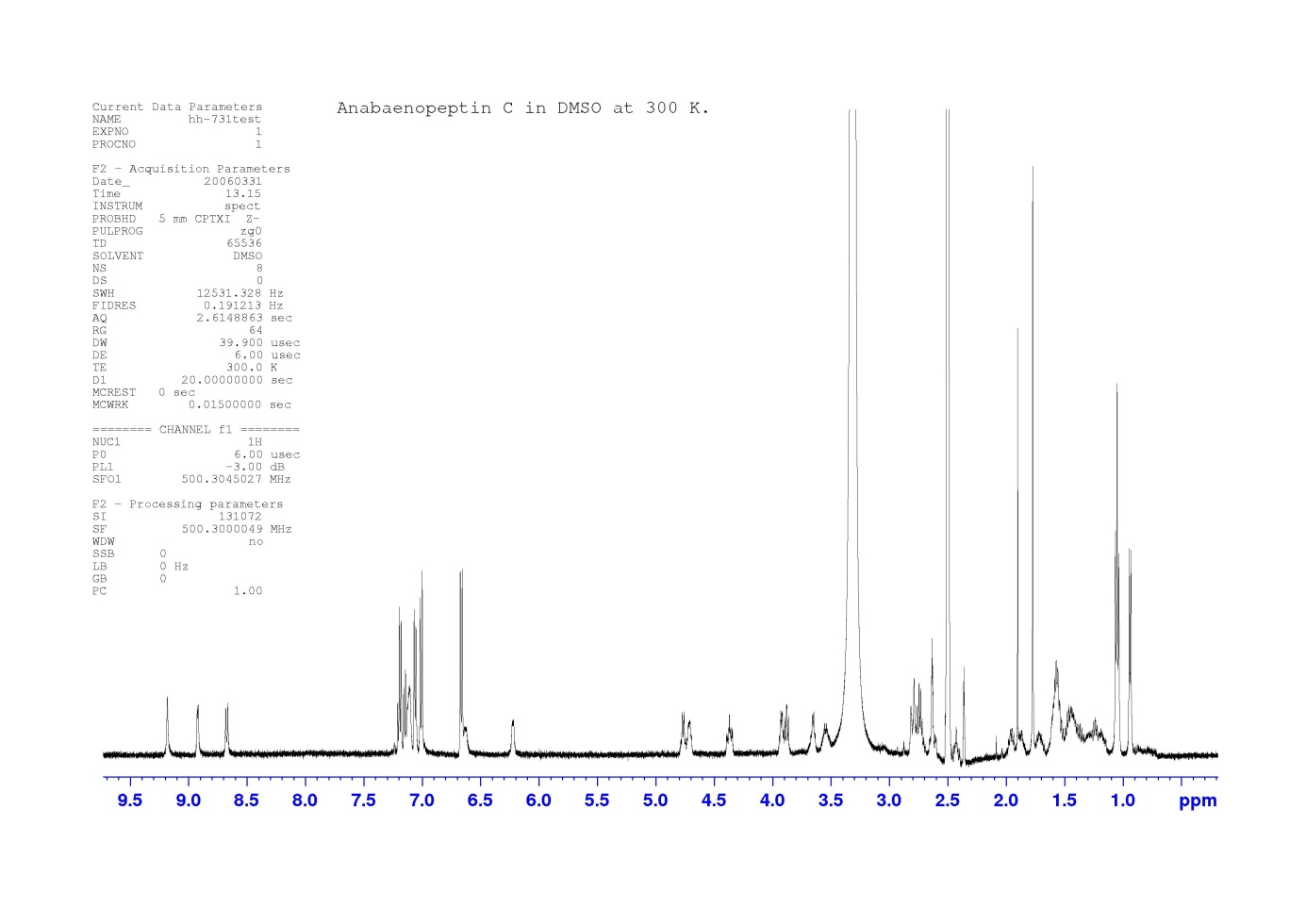
**

Fig. : ^1^H-spectrum of *Anabaenopeptin C* in DMSO at 300 K.

^1^H-NMR (DMSO-d_6_, 500MHz) δ 0.94 (d, 3 H), 1.04 (d, 3 H), 1.06 (d, 3 H), 1.96 – 1.12 (m, 15 H), 1.77 (s, 3H), 2.74 (m, 1 H), 2.79 (m, 1 H), 3.54 (m, 1 H), 3.65 (m, 1 H), 3.88 (m, 1 H), 3.92 (m, 1 H), 4.37 (m, 1 H), 4.71 (m, 1 H), 4.77 (m, 1 H), 6.22 (d, 1 H), 6.63 (m, 1 H), 6.67 (d, 2 H), 7.01 (d, 2 H), 7.06 (d, 2 H), 7.11 (m, 2 H), 7.14 (m, 1H), 7.20 (t, 2 H), 8.67 (d, 1 H), 8.92 (d, 1 H), 9.18 (s, 1H)

**17**

UV: 206, 277 nm

C42H62N8O9, Monoisotopic molecular mass (calc.): 822.46398 Da

Calc. [M+H^+^]: 823.4718 Da; observed [M+H^+^]: 823.4701 Da

Fig. 1 Structure of **17**


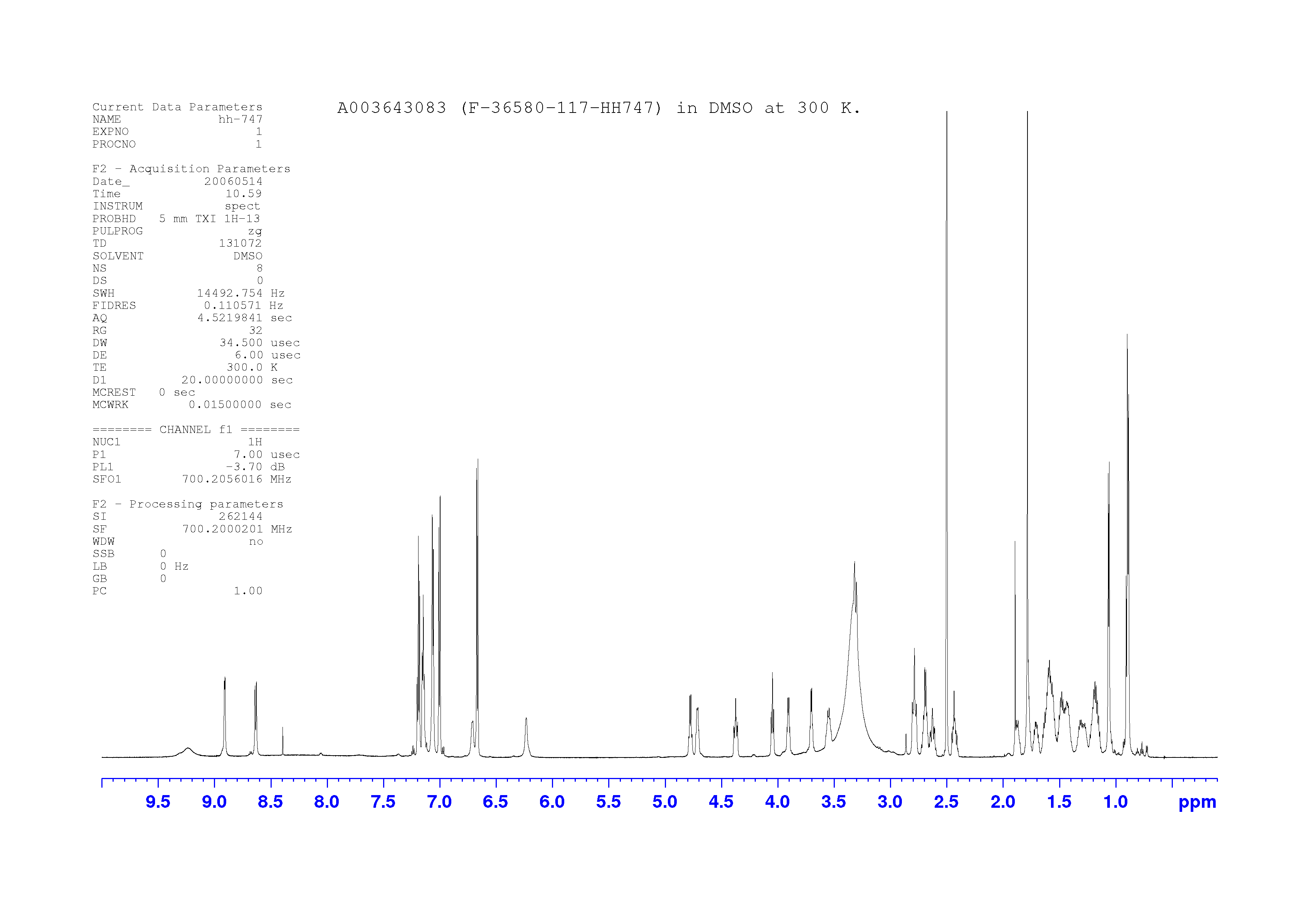


Fig. : ^1^H-spectrum of **17** in DMSO at 300 K.


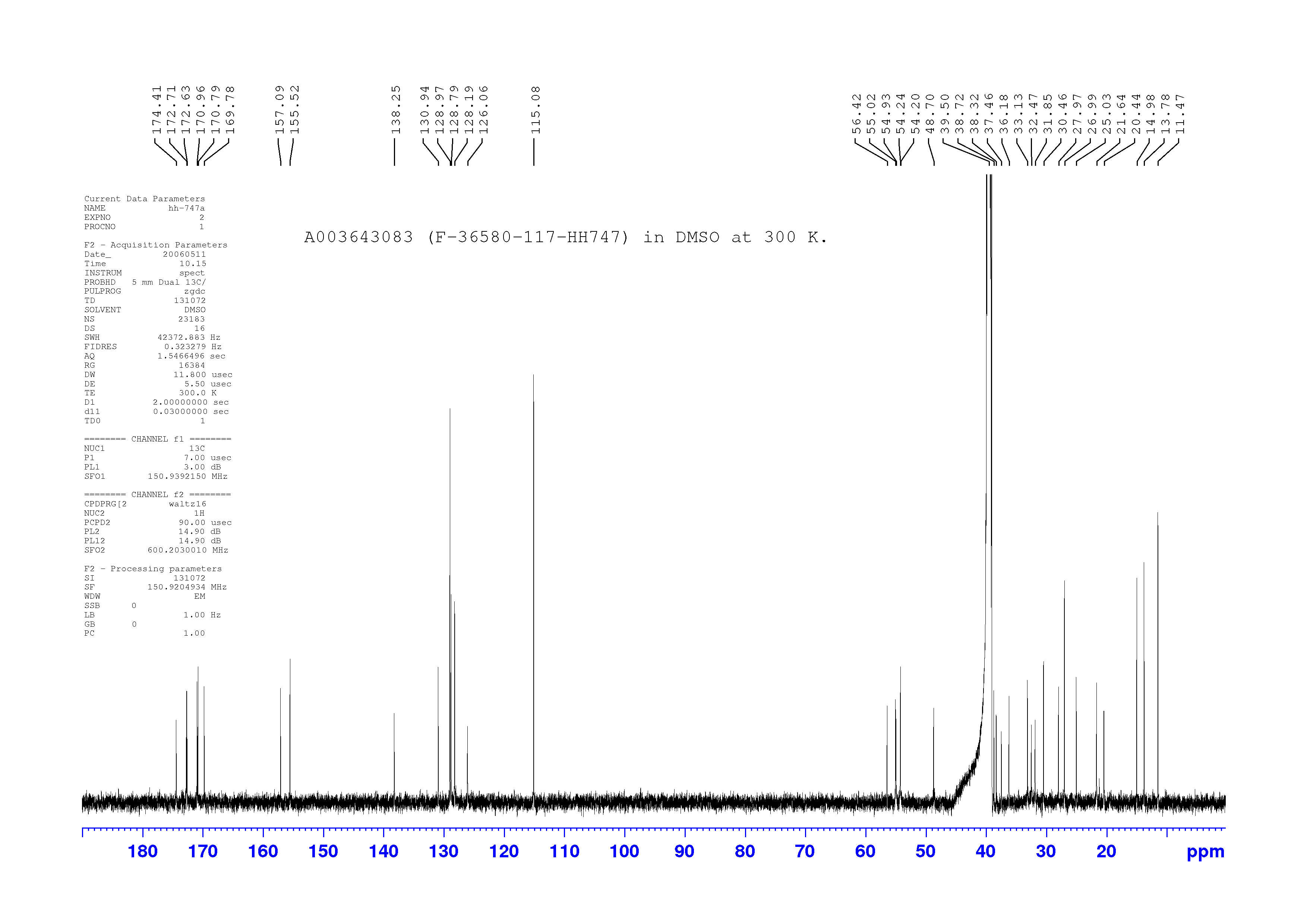


Fig. : ^13^C-spectrum of **17** in DMSO at 300 K.

Table 1: Chemical shifts of **17** in DMSO at 300 K.

|  | ^1^H | ^13^C |
| --- | --- | --- |
| Phe-1 NH | 8.62 | - |
| α | 4.37 | 55.02 |
| β | 3.30/2.78 | 37.46 |
| γ | - | 138.25 |
| δ | 7.06 | 128.79 |
| ε | 7.19 | 128.19 |
| ζ | 7.14 | 126.06 |
| C’ | - | 170.79 |
| N-Me-Ala-2 NMe | 1.78 | 26.99 |
| α | 4.77 | 54.20 |
| β | 1.06 | 13.78 |
| C’ | - | 169.78 |
| HTy-3 NH | 8.91 | - |
| α | 4.71 | 48.70 |
| β | 1.87/1.70 | 33.13 |
| homo-β | 2.62/2.43 | 30.46 |
| γ | - | 130.94 |
| δ | 7.00 | 128.97 |
| ε | 6.66 | 115.08 |
| ζ | - | 155.52 |
| ζ-OH | 9.24 | - |
| C’ | - | 170.96 |
| Ile-4 NH | 7.07 | - |
| α | 4.04 | 56.42 |
| β | 1.77 | 36.18 |
| β-Me | 0.88 | 14.98 |
| γ | 1.63/1.16 | 25.03 |
| δ | 0.89 | 11.47 |
| C’ | - | 172.71 |
| Lys-5 NH | 6.71 | - |
| α | 3.90 | 54.93 |
| β | 1.59 | 31.86 |
| γ | 1.27/1.19 | 20.44 |
| δ | 1.42 | 27.97 |
| ε | 3.54/2.79 | 38.32 |
| ζ-NH | 7.15 | - |
| C’ | - | 172.63 |
| Lys-6 NH | 6.23 | - |
| α | 3.70 | 54.24 |
| β | 1.56 | 32.47 |
| γ | 1.31/1.18 | 21.64 |
| δ | 1.48 | 26.99 |
| ε | 2.68 | 38.72 |
| C’ | - | 174.41 |
| 1’-C’ | - | 157.09 |

**Isolation of 20 and 19**

Fractions 1-9 from solid phase extraction of CBT344 were separately purified using a Phenomenex C18 AX column (dimension: 30 mm x 100 mm, 5 µm) with a Waters XTerra® pre-column (dimension: 19 x 10 mm, 10 µm). Compounds were eluted using a gradient of acetonitrile : 10 % formic acid (flow rate: 70 ml/min, 5 to 95 % within 43 min). The eluents have been collected in 45 ml-fractions using UV-triggering. The purification of fractions 1 and 2 gave highly pure material of **20** (5 mg). Fractions containing **19** were combined, freeze dried and further purified using a Phenomenex AX C18 column (dimension: 21 mm x 100 mm, 5 µm) with a Waters XTerra® pre-column (dimension: 19 x 10 mm, 10 µm). Compounds were eluted using a gradient of acetonitrile : 10 % formic acid (flow rate: 50 ml/min, 5 to 95 % within 43 min). After freeze-drying, pure material of **19** (4 mg) was obtained.

**20**

UV: 206, 217s, 278 nm

C44H57N7O11, Monoisotopic molecular mass (calc.): 859.4116 Da

Calc. [M+H^+^]: 860.4194 Da; observed [M+H^+^]: 860.4232 Da

Fig. 1 Structure of **20**


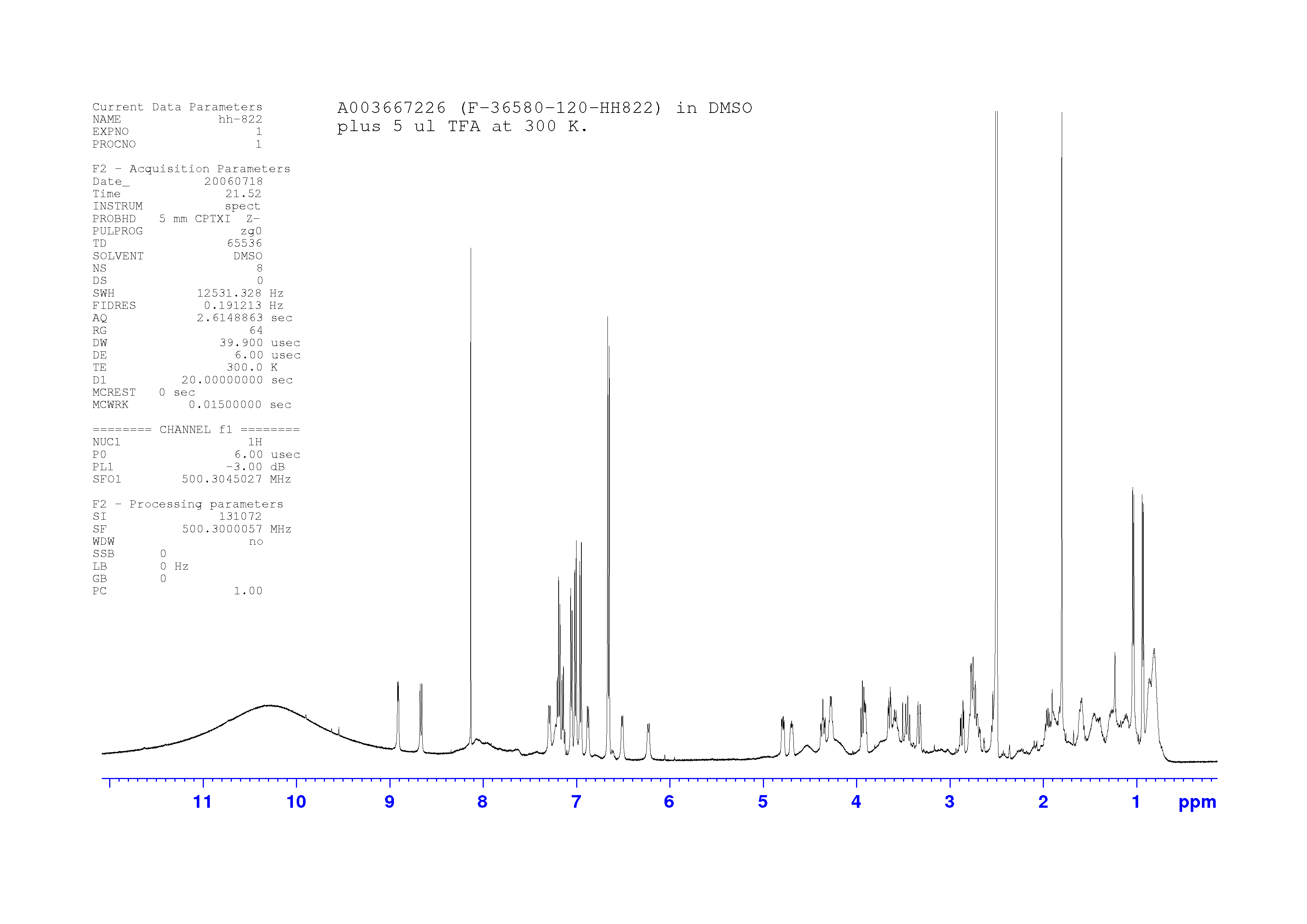


Fig. : ^1^H-spectrum of **20** in DMSO (after addition of 5 μL TFA) at 300 K.


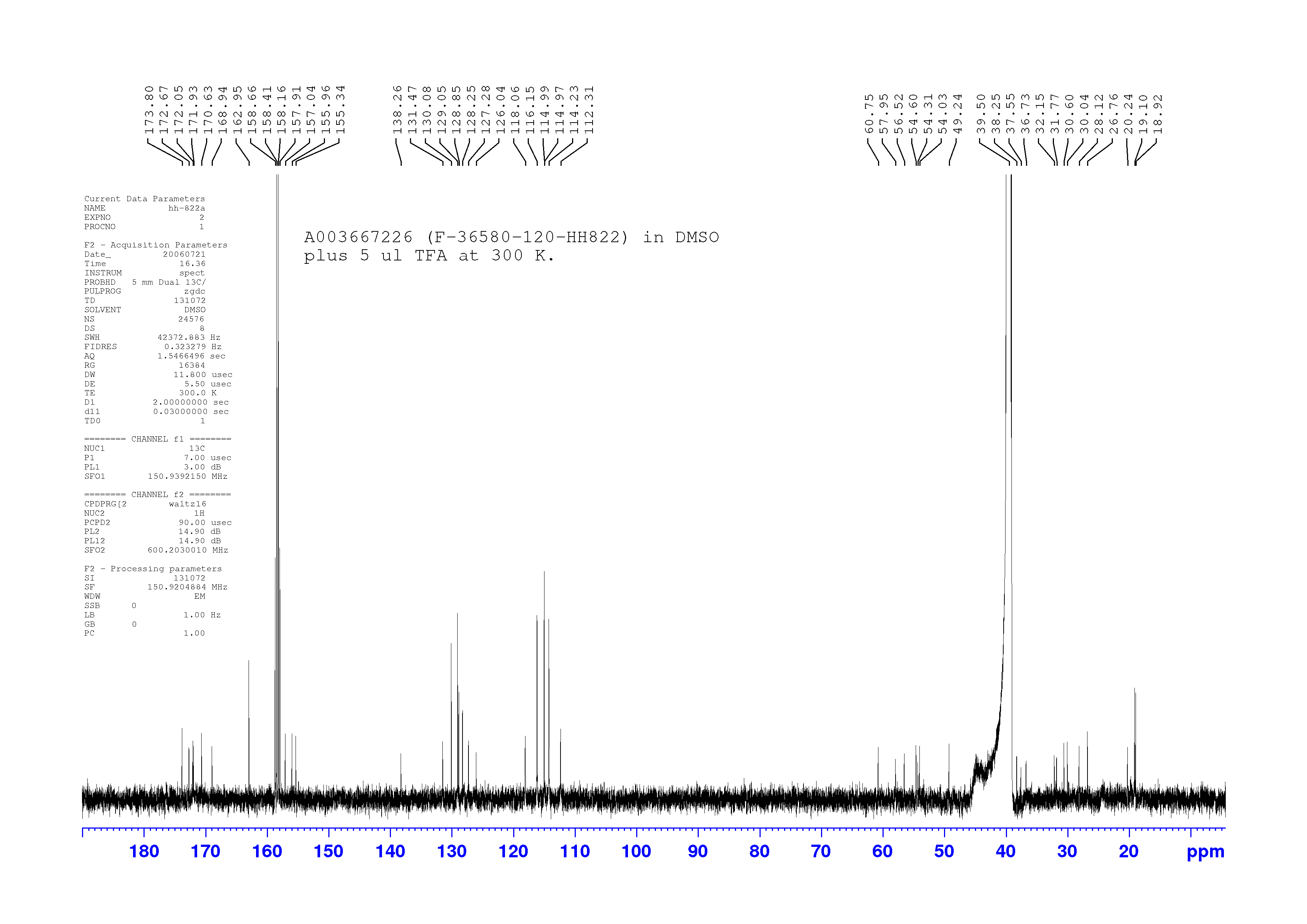


Fig. : ^1^H-spectrum of **20** in DMSO (after addition of 5 μL TFA) at 300 K.Table 1: Chemical shifts of **20** in DMSO (after addition of 5 μL TFA) at 300 K.

|  | ^1^H | ^13^C |
| --- | --- | --- |
| Phe-1 NH | 8.66 | - |
| α | 4.36 | 54.31 |
| β | 3.33/2.74 | 37.55 |
| γ | - | 138.26 |
| δ | 7.05 | 128.85 |
| ε | 7.18 | 128.25 |
| ζ | 7.13 | 126.04 |
| C’ | - | 170.63 |
| N-Me-Ser-2 NMe | 1.80 | 26.76 |
| α | 4.78 | 60.75 |
| β | 3.64/3.44 | 56.52 |
| β-OH | broad | - |
| C’ | - | 168.94 |
| HTy-3 NH | 8.91 | - |
| α | 4.69 | 49.24 |
| β | 1.89/1.82 | 32.15 |
| homo-β | 2.70/2.52 | 30.60 |
| γ | - | 131.47 |
| δ | 7.01 | 129.05 |
| ε | 6.65 | 114.99 |
| ζ | - | 155.34 |
| ζ-OH | broad | - |
| C’ | - | 171.93 |
| Val-4 NH | 6.87 | - |
| α | 3.93 | 57.95 |
| β | 1.95 | 30.04 |
| γ | 1.03 | 18.92 |
| γ’ | 0.93 | 19.10 |
| C’ | - | 172.67 |
| Lys-5 NH | 6.50 | - |
| α | 3.90 | 54.60 |
| β | 1.59 | 31.77 |
| γ | 1.27/1.11 | 20.24 |
| δ | 1.42 | 28.12 |
| ε | 3.58/2.77 | 38.25 |
| ζ-NH | 7.28 | - |
| C’ | - | 172.05 |
| Tyr-6 NH | 6.23 | - |
| α | 4.27 | 54.03 |
| β | 2.87/2.75 | 36.72 |
| γ | - | 127.28 |
| δ | 6.95 | 130.08 |
| ε | 6.65 | 114.97 |
| ζ | - | 155.96 |
| C’ | - | 173.80 |
| 1’-C’ | - | 157.04 |

**19**

UV: 206, 220s, 275 nm

C41H60N10O10, Monoisotopic molecular mass (calc.): 852.44939 Da

Calc. [M+H^+^]: 853.4572 Da; observed [M+H^+^]: 853.4603 Da

Fig. 1 Structure of **19**


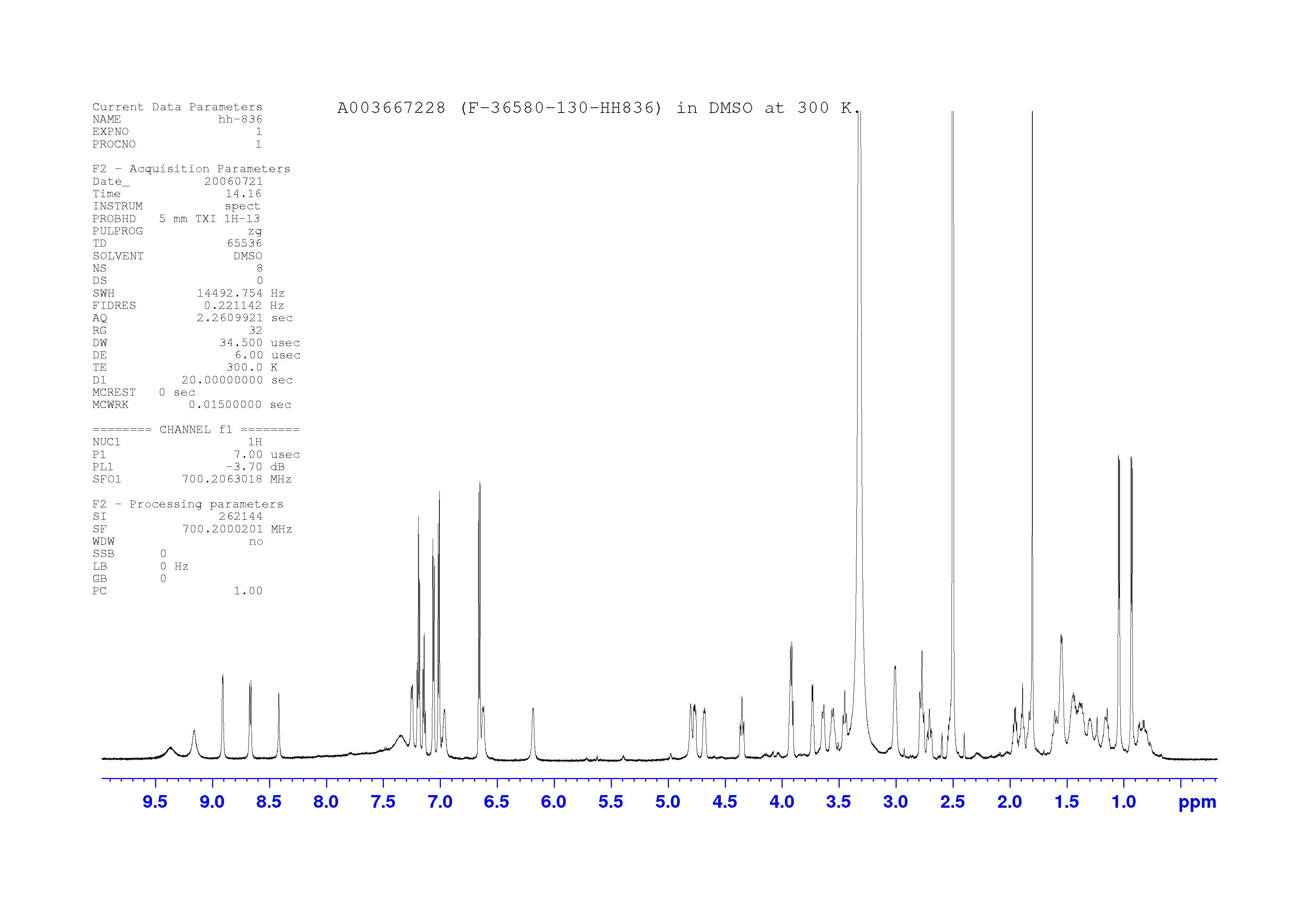


Fig. : ^1^H-spectrum of **19** in DMSO at 300 K.


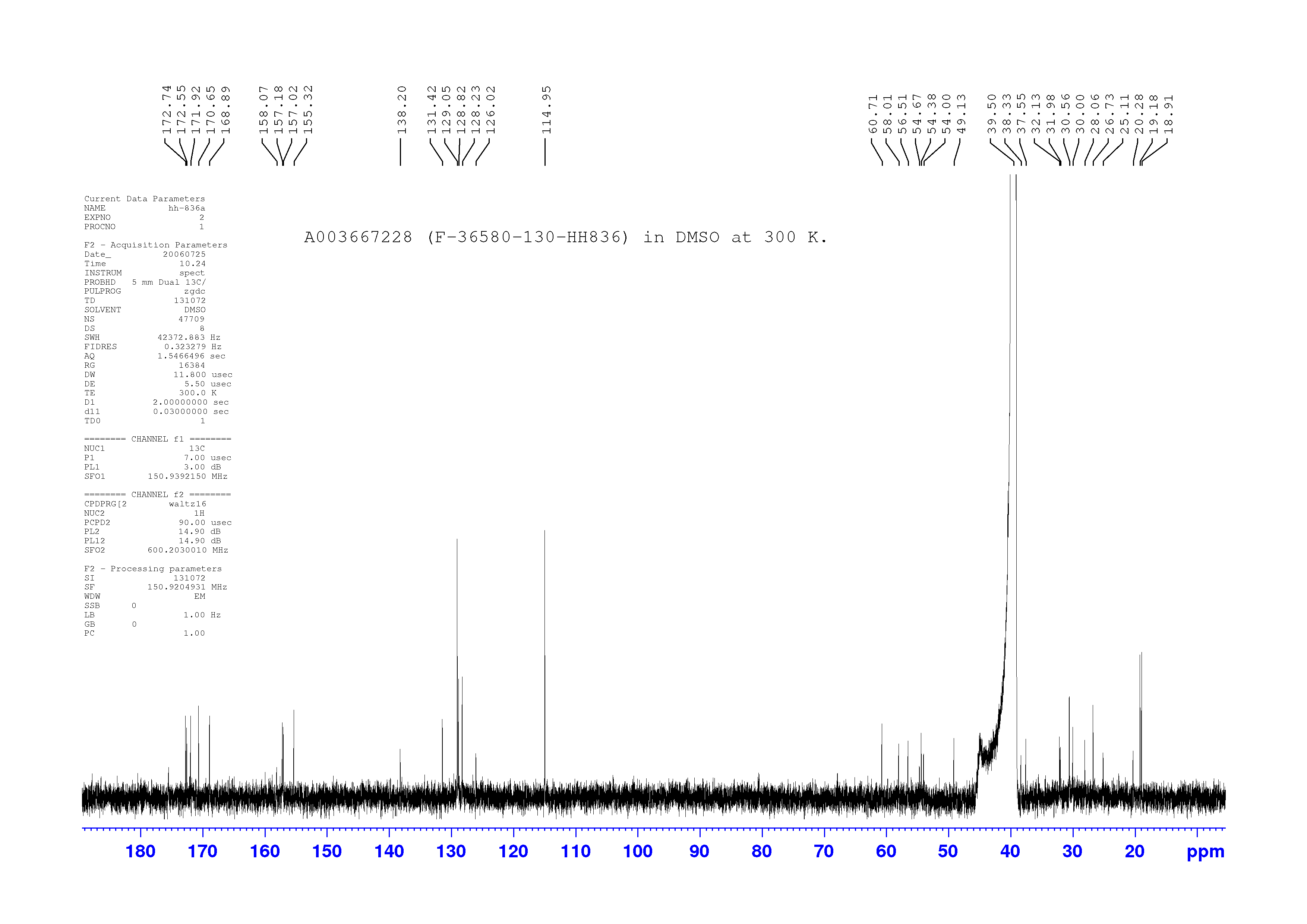


Fig. : ^13^C-spectrum of **19** in DMSO at 300 K.

Table 1: Chemical shifts of **19** in DMSO at 300 K.

|  | ^1^H | ^13^C |
| --- | --- | --- |
| Phe-1 NH | 8.67 | - |
| α | 4.35 | 54.38 |
| β | 3.32/2.77 | 37.55 |
| γ | - | 138.20 |
| δ | 7.06 | 128.82 |
| ε | 7.19 | 128.23 |
| ζ | 7.14 | 126.02 |
| C’ | - | 170.65 |
| N-Me-Ser-2 NMe | 1.80 | 26.73 |
| α | 4.76 | 60.71 |
| β | 3.64/3.45 | 56.51 |
| β-OH | n.a. | - |
| C’ | - | 168.89 |
| HTy-3 NH | 8.91 | - |
| α | 4.68 | 49.13 |
| β | 1.90/1.83 | 32.13 |
| homo-β | 2.71/2.53 | 30.56 |
| γ | - | 131.42 |
| δ | 7.01 | 129.05 |
| ε | 6.65 | 114.96 |
| ζ | - | 155.32 |
| ζ-OH | n.a. | - |
| C’ | - | 171.92 |
| Val-4 NH | 6.96 | - |
| α | 3.91 | 58.01 |
| β | 1.95 | 30.00 |
| γ | 1.04 | 18.91 |
| γ’ | 0.93 | 19.18 |
| C’ | - | 172.74 |
| Lys-5 NH | 6.61 | - |
| α | 3.92 | 54.67 |
| β | 1.60/1.55 | 31.98 |
| γ | 1.30/1.16 | 20.28 |
| δ | 1.45/1.39 | 28.06 |
| ε | 3.55/2.78 | 38.33 |
| ζ-NH | 7.24 | - |
| C’ | - | 172.55 |
| Arg-6 NH | 6.18 | - |
| α | 3.73 | 54.00 |
| β | 1.54 | 30.56 |
| γ | 1.42/1.35 | 25.11 |
| δ | 3.02 | ~ 40.4 |
| ε | n.a. | - |
| ζ | - | 157.18 |
| C’ | - | 157.02 |
| 1’-C’ | - | 157.04 |

n.a.: not assigned

**Isolation of 5, 7 and 8**

Fractions 3-4 and 5-7 from solid phase extraction CBT158 were purified using a Phenomenex C18 column (dimension: 50 mm x 100 mm, 10 µm) with a Waters XTerra® pre-column (dimension: 19 x 10 mm, 10 µm). Compounds were eluted using a gradient of acetonitrile : ammonium acetate buffer pH 7.0 (flow rate: 120 ml/min, 5 to 95 % within 27 min). The eluents have been collected using UV-triggering. Fractions containing compounds of interest were combined and freeze dried. Pre-purified fractions 2-3 were further purified using a Phenomenex C18 column (dimension: 21 mm x 100 mm, 5 µm) with a Waters XTerra® pre-column (dimension: 19 x 10 mm, 10 µm) applying a gradient of acetonitrile : 10 % formic acid (flow rate: 70 ml/min, 5 to 95 % within 27 min). After freeze drying pure material of **5** (47 mg) and **7** (39 mg) was obtained. Pre-purified fraction 12 was further purified using a Phenomenex C18 column (dimension: 21 mm x 100 mm, 5 µm) with a Waters XTerra® pre-column (dimension: 19 x 10 mm, 10 µm) applying a gradient of acetonitrile : 10 % formic acid pH 2.0 (flow rate: 45 ml/min, 5 to 55 % at 27 min, 55 to 95 % at 29 min). After freeze drying pure material of **8** (4 mg) was obtained.

**5**

UV: 209 nmx

C44H65N11O9, Monoisotopic molecular mass (calc.): 891.4967 Da

Calc. [M-H^-^]: 890.4888 Da; observed [M-H^-^]: 890.4953 Da

Fig. 1: Structure of **5**


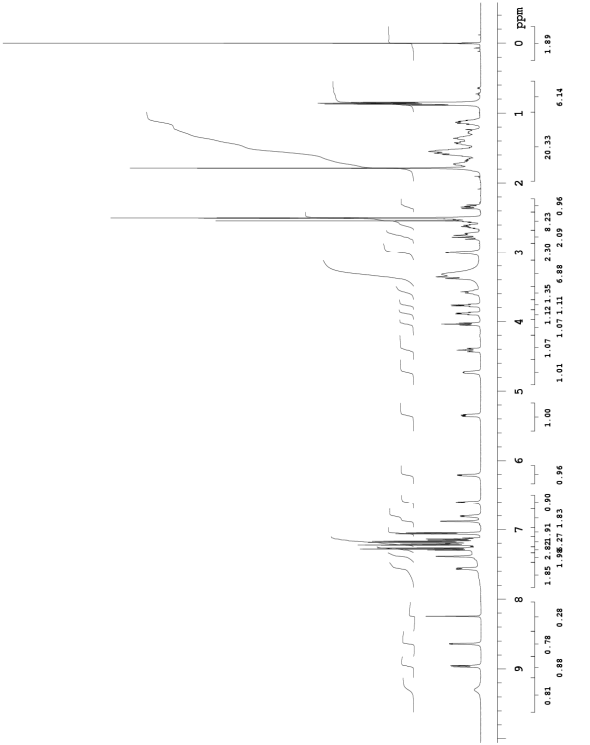


Fig.: ^1^H NMR Spectrum of **5** in DMSO at 298 K


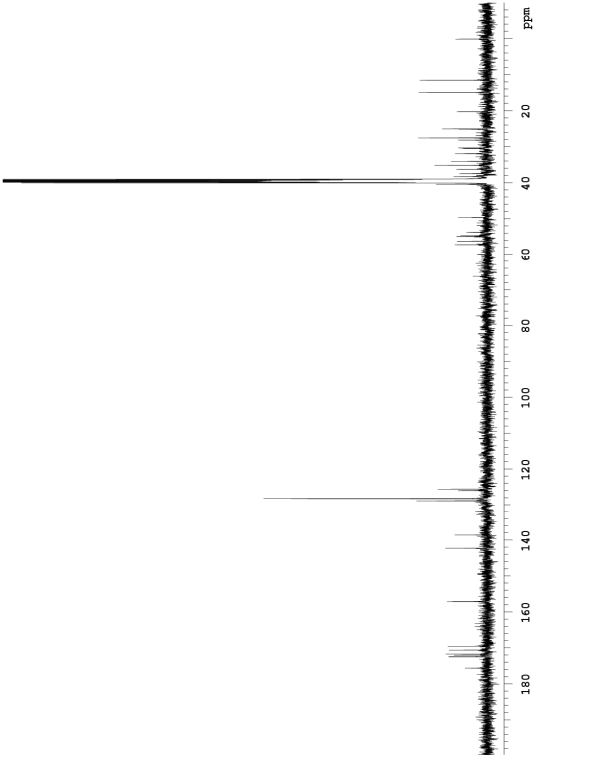


Fig.: ^13^C NMR Spectrum of **5** in DMSO at 298 K

Table 1: Chemical shifts of **5** in DMSO at 298 K.

|  | ^1^H | ^13^C |
| --- | --- | --- |
| Phe-1 NH | 8.98 | - |
| α | 4.42 | 54.95 |
| β | 3.35,2.79 | 37.33 |
| γ | - | 138.66 |
| δ | 7.05 | 128.89 |
| ε | 7.17 | 128.15 |
| ζ | 7.14 | 125.92 |
| C’ | - | 169.61 |
| N-Me-Asn-2 NMe | 1.80 | 27.42 |
| α | 5.34 | 57.41 |
| β | 2.35,2.32 | 33.95 |
| γ | - | 172.13 |
| NH2 | 6.88/7.39 | - |
| C’ | - | 169.75 |
| APPA-3 NH | 8.66 | - |
| α | 4.75 | 49.7 |
| β | 1.81/1.63 | 30.26 |
| Β’ | 1.79/1.67 | 27.40 |
| Β’’ | 2.6/2.52 | 35.28 |
| γ | - | 142.37 |
| δ | 7.22 | 128.27 |
| ε | 7.28 | 128.24 |
| ζ | 7.17 | 125.73 |
| C’ | - | 172.19 |
| Ile-4 NH | 6.82 | - |
| α | 4.05 | 56.33 |
| β | 1.73 | 36.05 |
| β-Me | 0.86 | 15.06 |
| γ | 1.59/1.1 | 24.95 |
| δ | 0.86 | 11.56 |
| C’ | - | 172.56 |
| Lys-5 NH | 6.61 | - |
| α | 3.89 | 54.95 |
| β | 1.57 | 30.57 |
| γ | 1.27/1.15 | 20.31 |
| δ | 1.45 | 28.29 |
| ε | 3.58/2.74 | 38.12 |
| ζ-NH | 7.57 | - |
| C’ | - | 172.69 |
| Arg-6 NH | 6.21 | - |
| α | 3.76 | 53.84 |
| β | 1.54/1.59 | 31.78 |
| γ | 1.35,1.43 | 25.02 |
| δ | 3.0 | 40.45 |
| ε-NH | 9.31 | - |
| ζ | - | 157.53 |
| C’ | - | 175.60 |
| 1’-C’ | - | 157.20 |

APPA=2-Amino-5-phenylpentanoic acid

**7**

UV: end

C44H64N8O9, Monoisotopic molecular mass (calc.): 848.4796 Da

Calc. [M+H^+^]: 849.4875 Da; observed [M+H^+^]: 849.4866 Da

Fig. 1 Structure of **7**


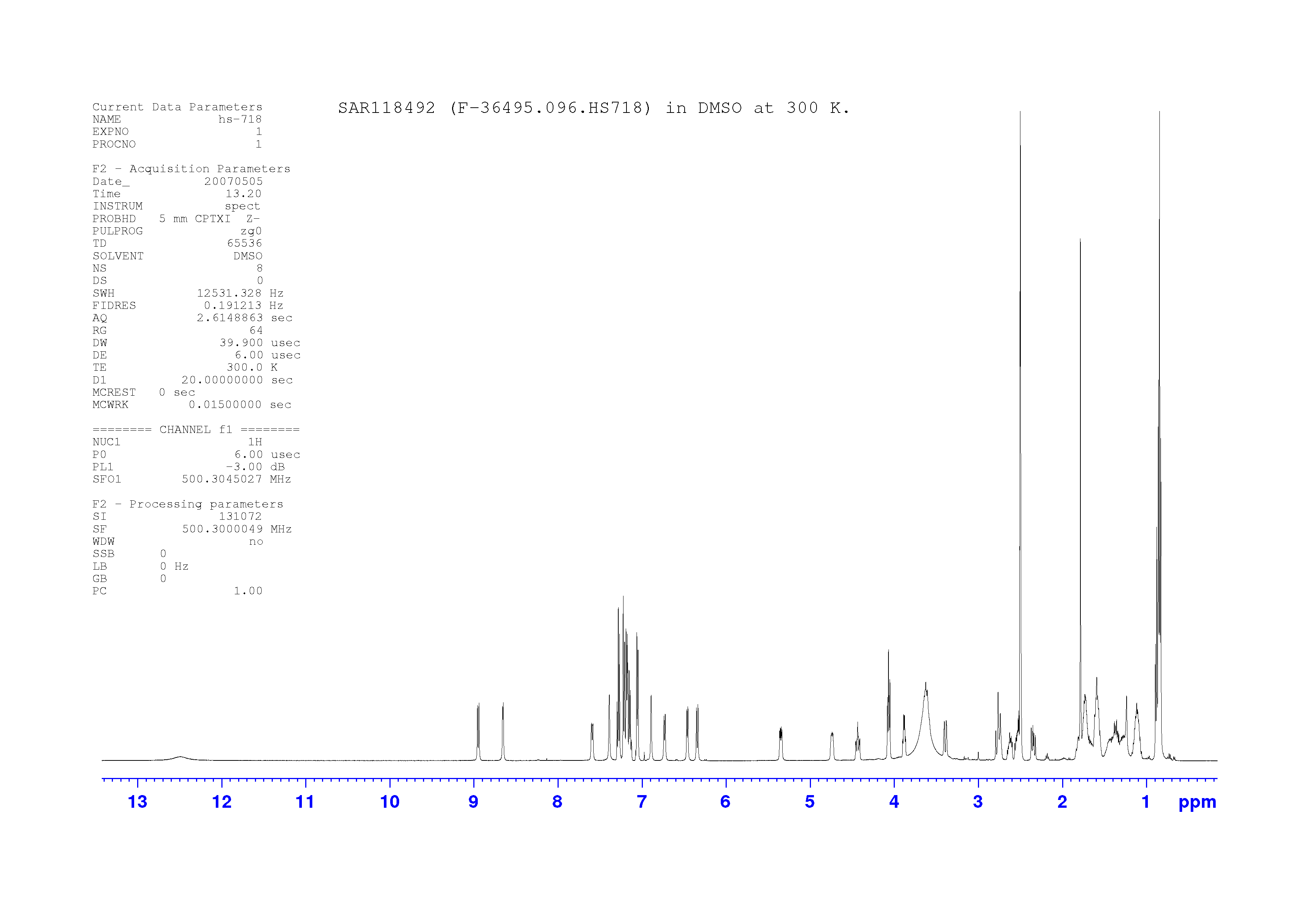


Fig. : ^1^H-spectrum of **7** in DMSO at 300K.


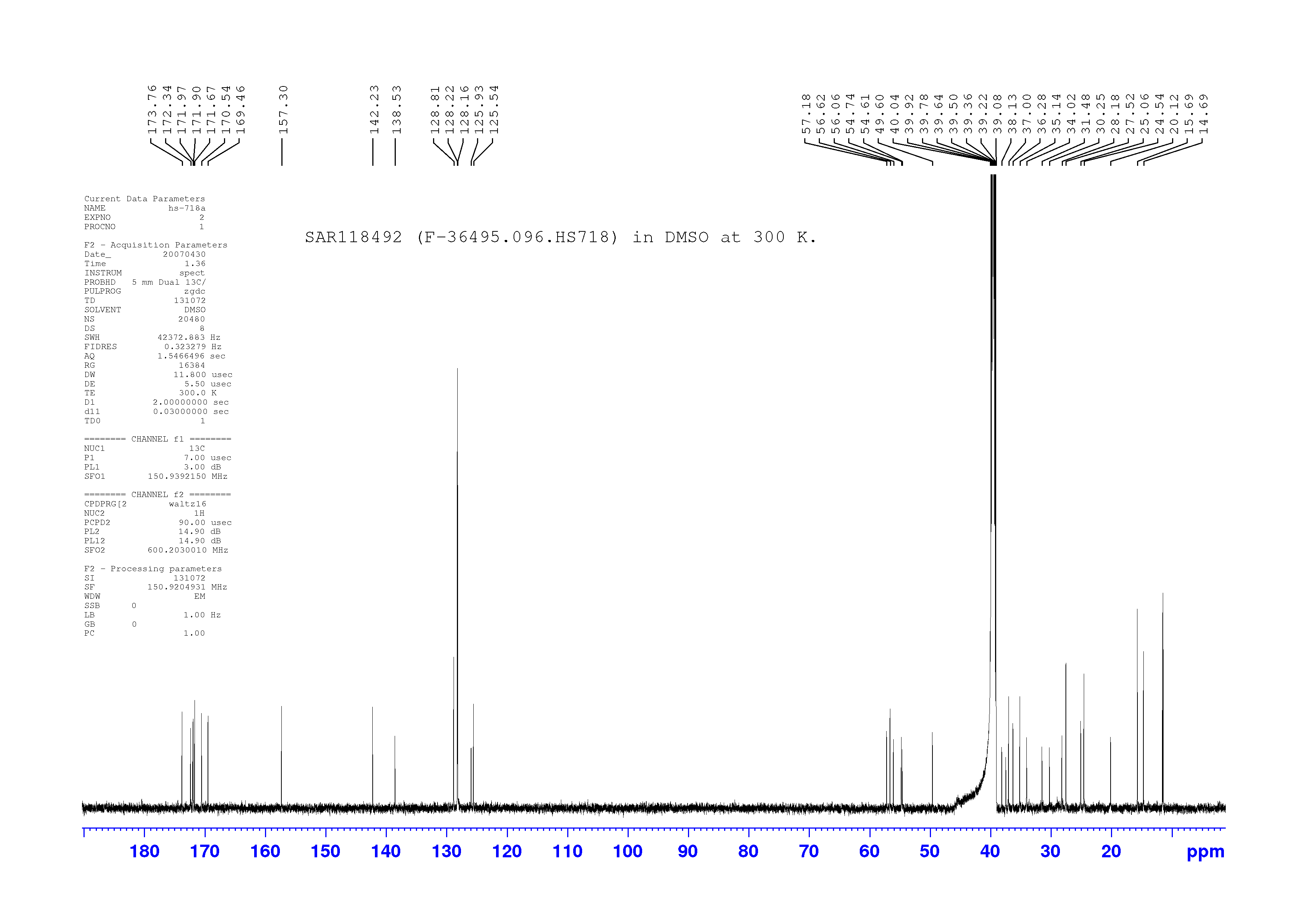


Fig. : ^13^C-spectrum of **7** in DMSO at 300K.

Table 1: Chemical shifts of **7** in DMSO at 300 K.

|  | ^1^H | ^13^C |
| --- | --- | --- |
| Phe-1 NH | 8.95 | - |
| α | 4.43 | 54.74 |
| β | 3.39/2.77 | 37.46 |
| γ | - | 138.53 |
| δ | 7.05 | 128.81 |
| ε | 7.19 | 128.16 |
| ζ | 7.14 | 125.93 |
| C’ | - | 170.54 |
| N-Me-Asn-2 NMe | 1.79 | 27.52 |
| α | 5.35 | 57.18 |
| β | 2.51/2.35 | 34.02 |
| γ | - | 171.67 |
| NH2 | 7.39/6.89 | - |
| C’ | - | 169.46 |
| APPA-3 NH | 8.65 | - |
| α | 4.74 | 49.60 |
| β | 1.81/1.59 | 30.25 |
| Β’ | 1.76/1.68 | 27.52 |
| Β’’ | 2.62/2.54 | 35.14 |
| γ | - | 142.23 |
| δ | 7.22 | 128.22 |
| ε | 7.28 | 128.16 |
| ζ | 7.17 | 125.54 |
| C’ | - | 171.97 |
| Ile-4 NH | 6.73 | - |
| α | 4.07 | 56.06 |
| β | 1.73 | 36.28 |
| β-Me | 0.85 | 14.69 |
| γ | 1.58/1.13 | 25.06 |
| δ | 0.88 | 11.52 |
| C’ | - | 172.34 |
| Lys-5 NH | 6.46 | - |
| α | 3.88 | 54.61 |
| β | 1.60 | 31.48 |
| γ | 1.29/1.11 | 20.12 |
| δ | 1.42 | 28.18 |
| ε | 3.62/2.75 | 38.13 |
| ζ-NH | 7.60 | - |
| C’ | - | 171.90 |
| Ile-6 NH | 6.34 | - |
| α | 4.06 | 56.62 |
| β | 1.72 | 37.00 |
| β-Me | 0.84 | 15.69 |
| γ | 1.35/1.11 | 24.54 |
| δ | 0.84 | 11.43 |
| C’ | - | 173.76 |
| 1’-C’ | - | 157.30 |

APPA=2-Amino-5-phenylpentanoic acid

**8**

UV: 220 nm

C45H66N8O9, Monoisotopic molecular mass (calc.): 862.4952 Da

Calc. [M+H]+: 863.5031 Da; Observed [M+H]+: 863.5031

Fig. 1: Structure of **8**


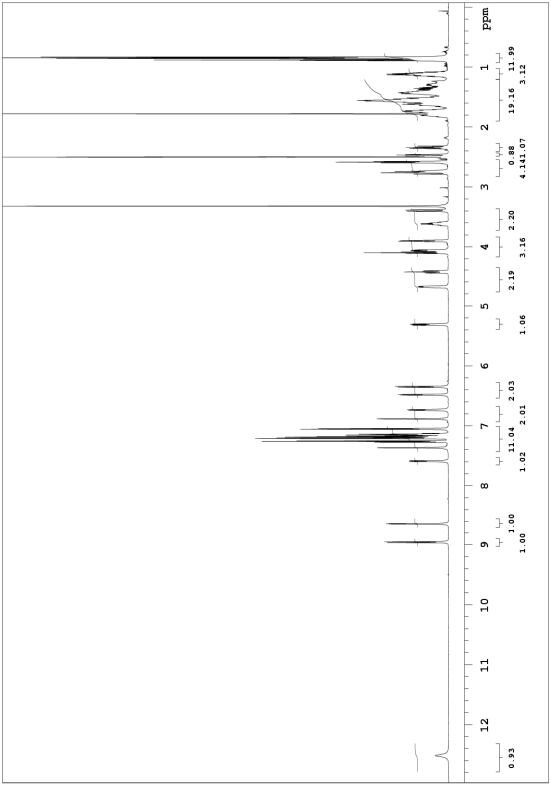


Fig.: ^1^H NMR Spectrum of **8** in DMSO at 298K

Fig.: ^13^C NMR Spectrum of **8** in DMSO at 298K

Table 1: Chemical shifts of **8** in DMSO at 298 K.

|  | ^1^H | ^13^C |
| --- | --- | --- |
| Phe-1 NH | 8.96 | - |
| α | 4.43 | 54.73 |
| β | 3.39/2.76 | 37.48 |
| γ | - | 138.56 |
| δ | 7.05 | 128.83 |
| ε | 7.19 | 128.18 |
| ζ | 7.14 | 125.94 |
| C’ | - | 170.57 |
| N-Me-Asn-2 NMe | 1.78 | 27.54 |
| α | 5.31 | 57.16 |
| β | 2.47/2.34 | 34.03 |
| γ | - | 171.63 |
| NH2 | 7.37/6.88 | - |
| C’ | - | 169.48 |
| APHA-3 NH | 8.64 | - |
| α | 4.68 | 49.67 |
| β | 1.81/1.53 | 30.24 |
| Β’ | 1.43 | 25.46 |
| Β’’ | 1.57 | 31.13 |
| B’’’ | 2.59 | 35.38 |
| γ | - | 142.44 |
| δ | 7.22 | 128.26 |
| ε | 7.26 | 128.18 |
| ζ | 7.16 | 125.52 |
| C’ | - | 172.13 |
| Ile-4 NH | 6.74 | - |
| α | 4.09 | 55.97 |
| β | 1.76 | 36.29 |
| β-Me | 0.84 | 14.68 |
| γ | 1.55/1.14 | 25.13 |
| δ | 0.88 | 11.56 |
| C’ | - | 172.35 |
| Lys-5 NH | 6.48 | - |
| α | 3.90 | 54.66 |
| β | 1.62 | 31.51 |
| γ | 1.30/1.13 | 20.14 |
| δ | 1.42 | 28.18 |
| ε | 3.61/2.74 | 38.13 |
| ζ-NH | 7.59 | - |
| C’ | - | 171.93 |
| Ile-6 NH | 6.35 | - |
| OH | 12.52 | - |
| α | 4.06 | 56.69 |
| β | 1.73 | 37.03 |
| β-Me | 0.84 | 15.72 |
| γ | 1.37/1.11 | 24.56 |
| δ | 0.84 | 11.47 |
| C’ | - | 173.79 |
| 1’-C’ | - | 157.33 |

APHA=2-Amino-6-phenylhexanoic acid

### Isolation of 15 and 16

Extraction of biomass of CBT292

82g of dried biomass were extracted with a mixture of water : methanol 1:1 repeatedly. All extracts were combined and freeze dried to give 51g of crude extract lyophilisate.

Solid phase extraction of CBT292

51g of the dried extract were dissolved in altogether 4.3 L mixture of water : methanol 1:1, the pH of this extract was 4.9. The crude extract was loaded onto a column filled with ~4.3L of CHP20-P (MCI® Gel, 75-150µ, Mitsubishi Chemical Corporation) material.
A gradient with ammonium acetate (50g NH_4_Ac /L H_2_O adjusted to pH 4.6 with acetic acid): acetonitrile was applied (50% to 100% within 80 minutes, flow rate 90mL/min). Fractions were collected every 3 minutes. The fractions containing the compounds of interest were freeze-dried and further purified.

Isolation of **15** and **16**

Fraction 14 from the solid phase extraction was separately purified using a Phenomenex Luna C18(2) column (dimension: 21x250mm, 10µm) with a Waters X-Terra pre-column (dimension: 19x10mm, 10µm).
~188mg of the freeze-dried mixture was dissolved in 15mL acetonitrile / water and loaded onto the column via a preparative injection valve. The compounds were eluted using a gradient of 0.1% trifluoroacetic acid: acetonitrile (20% to 60% within 45 minutes, flow rate: 60mL/min). Fractions were collected using UV-triggering (224nm ) in 30mL fractions.
The separation yielded 51.9 mg of anabaenopeptin 915 (**15**) after freeze-drying.

Fraction 12 from the initial solid phase extraction was submitted to the next purification step using the same Phenomenex Luna C18(2) column (dimension: 21x250mm, 10µm) equipped with a Waters pre-column (dimension: 19x10mm, 10µm). ~278mg of the freeze-dried fraction no.12 was dissolved in ~12mL acetonitrile /water, loaded onto the column using a preparative injection valve and further purified using a gradient of 0.1% formic acid: acetonitrile (15% to 30% within 45 minutes, flow rate: 60mL/min). Fractions were collected using UV-triggering (230nm ) in 30mL fractions. Fractions 12-20 contained the compounds of interest and were freeze-dried. They were then finally purified using a Waters Sunfire column (dimension: 19x100mm, 5µm) in combination with a Waters pre-column (dimension: 19x10mm, 10µm). ~25mg of freeze-dried material was dissolved in ~5mL acetonitrile/water and injected on the column. A gradient of 0.1% trifluoroacetic acid : acetonitrile (5% to 60% within 30 minutes, flow rate: 45mL/min)was applied. Fractions were collected using UV-triggering (224nm) in 9mL fractions. The sequence of separations yielded 10.5mg of **16** after freeze-drying.

**16**, Anabaenopeptin 908^[[1]](#footnote-1)^

UV: 203, 220, 278 nm

C_45_H_68_N_10_O_10_, monoisotopic molecular mass (calc.): 908.5120 Da
Calc. [M+H^+^]: 909.5198 Da; observed [M+H^+^]: 909.5180 Da

Fig. 1 Structure of **16**


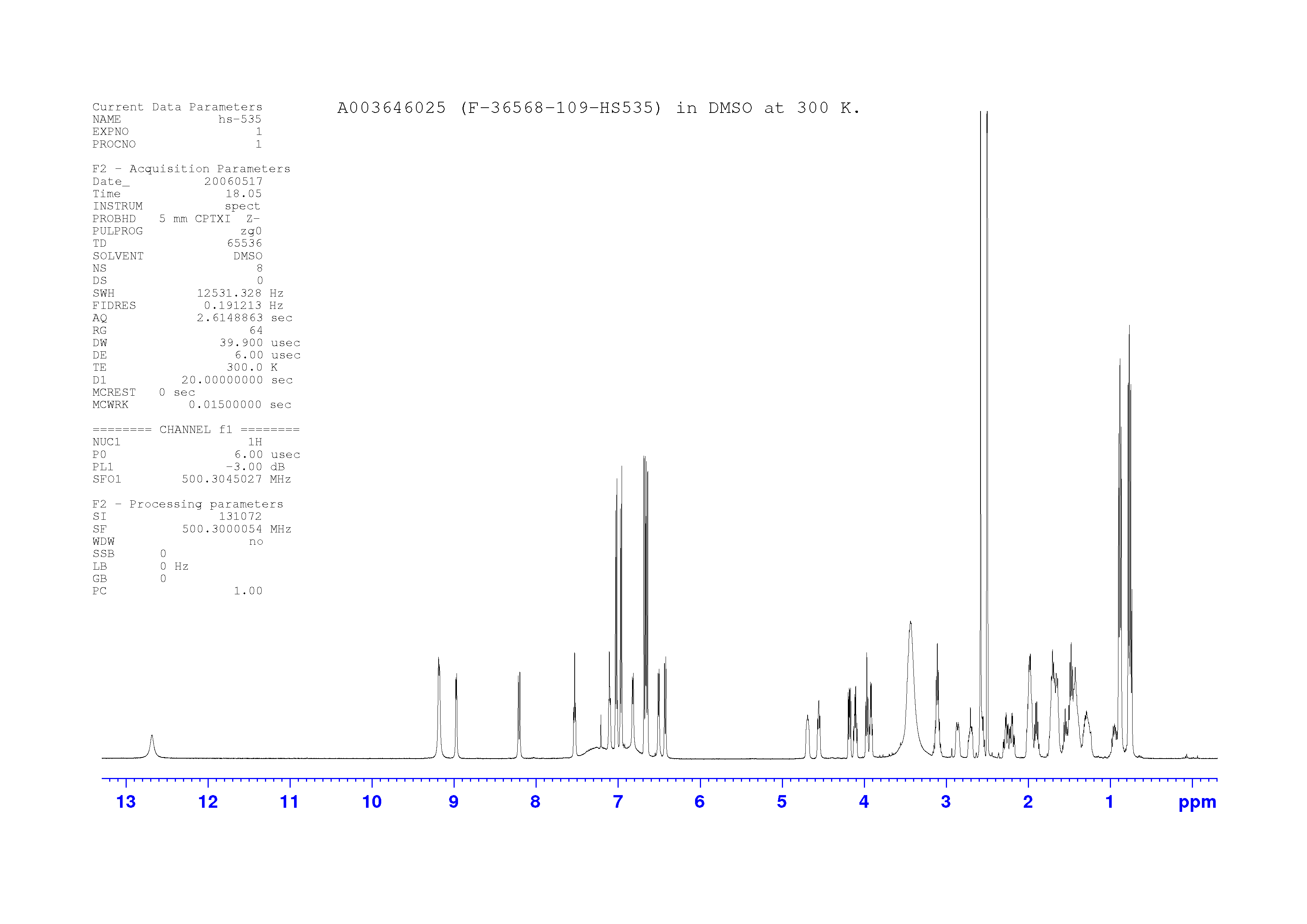


Fig. : ^1^H-spectrum of **16** in DMSO at 300 K.


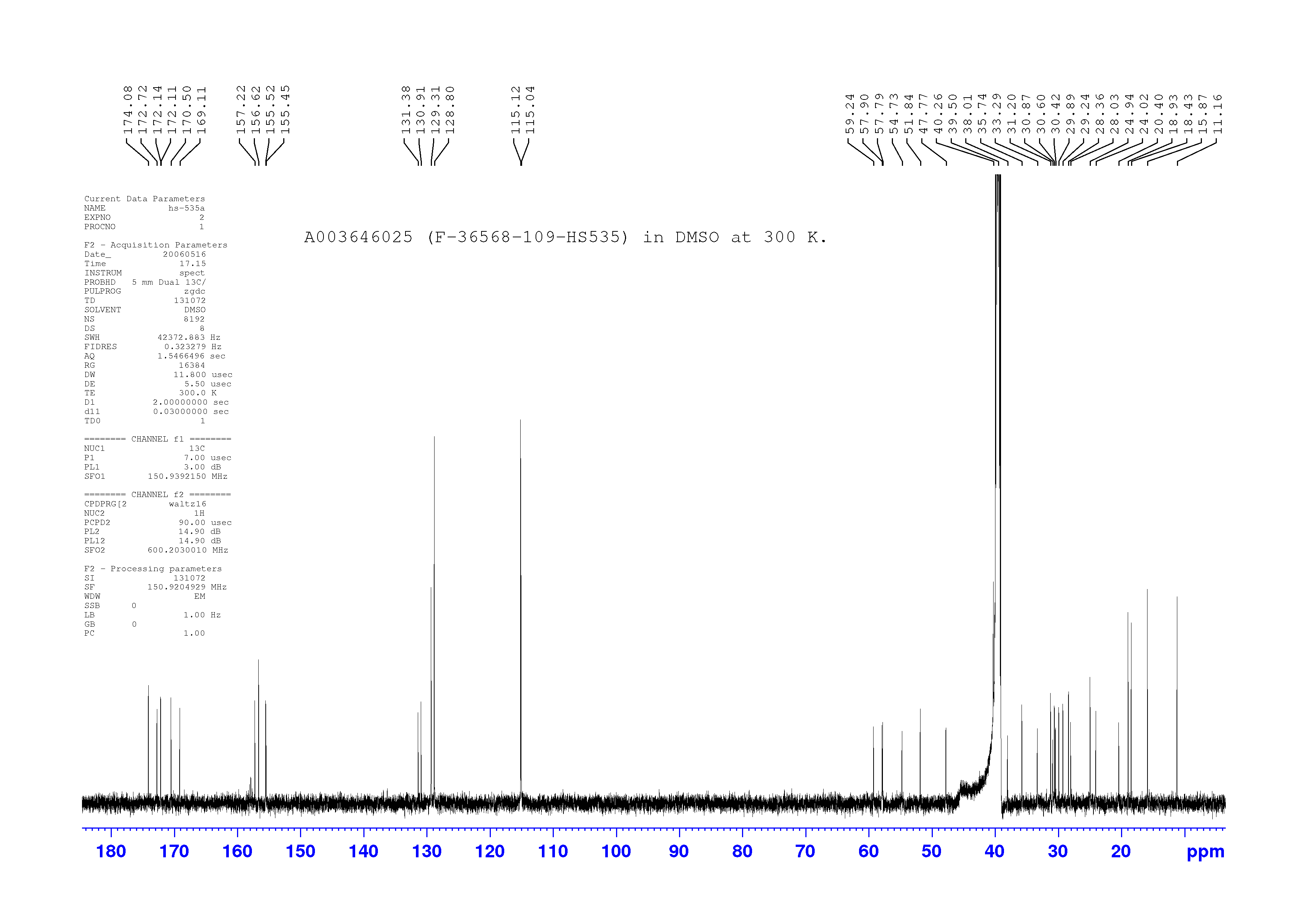


Fig. : ^13^C-spectrum of **16** in DMSO at 300 K.

Table 1: Chemical shifts of **16** in DMSO at 300 K.

|  | ^1^H | ^13^C |
| --- | --- | --- |
| Ile-1 NH | 8.20 | - |
| α | 4.18 | 57.90 |
| β | 1.97 | 35.74 |
| β-Me | 0.77 | 15.87 |
| γ | 1.30/0.94 | 24.02 |
| δ | 0.74 | 11.16 |
| C’ | - | 170.50 |
| HTy-2 NMe | 2.57 | 28.36 |
| α | 4.55 | 59.24 |
| β | 1.98/1.70 | 30.60 |
| homo-β | 2.26/2.19 | 31.20 |
| γ | - | 131.38 |
| δ | 6.96 | 128.80 |
| ε | 6.67 | 115.13 |
| ζ | - | 155.45 |
| ζ-OH | 9.18 | - |
| C’ | - | 169.11 |
| HTy-3 NH | 8.97 | - |
| α | 4.68 | 47.77 |
| β | 1.98/1.69 | 33.29 |
| homo-β | 2.69/2.56 | 30.42 |
| γ | - | 130.91 |
| δ | 7.02 | 129.31 |
| ε | 6.64 | 115.04 |
| ζ | - | 155.52 |
| ζ-OH | 9.19 | - |
| C’ | - | 172.11 |
| Val-4 NH | 6.82 | - |
| α | 3.96 | 57.79 |
| β | 1.90 | 29.90 |
| γ | 0.88 | 18.93 |
| γ’ | 0.87 | 18.43 |
| C’ | - | 172.72 |
| Lys-5 NH | 6.50 | - |
| α | 3.91 | 54.73 |
| β | 1.64 | 30.87 |
| γ | 1.40/1.26 | 20.40 |
| δ | 1.42 | 28.03 |
| ε | 3.45/2.86 | 38.01 |
| ζ-NH | 7.10 | - |
| C’ | - | 172.14 |
| Arg-6 NH | 6.42 | - |
| α | 4.10 | 51.84 |
| β | 1.70/1.54 | 29.25 |
| γ | 1.47 | 24.94 |
| δ | 3.10 | 40.26 |
| ε | 7.53 | - |
| ζ | - | 156.62 |
| C’ | - | 174.08 |
| 1’-C’ | - | 157.22 |

**15**, Anabaenopeptin 915^[[2]](#footnote-2)^

UV: 201, 223, 278 nm

C_48_H_65_N_7_O_11_, monoisotopic molecular mass (calc.): 915.4742 Da
Calc. [M+H^+^]: 916.4820 Da; observed [M+H^+^]: 916.4817

Fig. 1 Structure of **15**


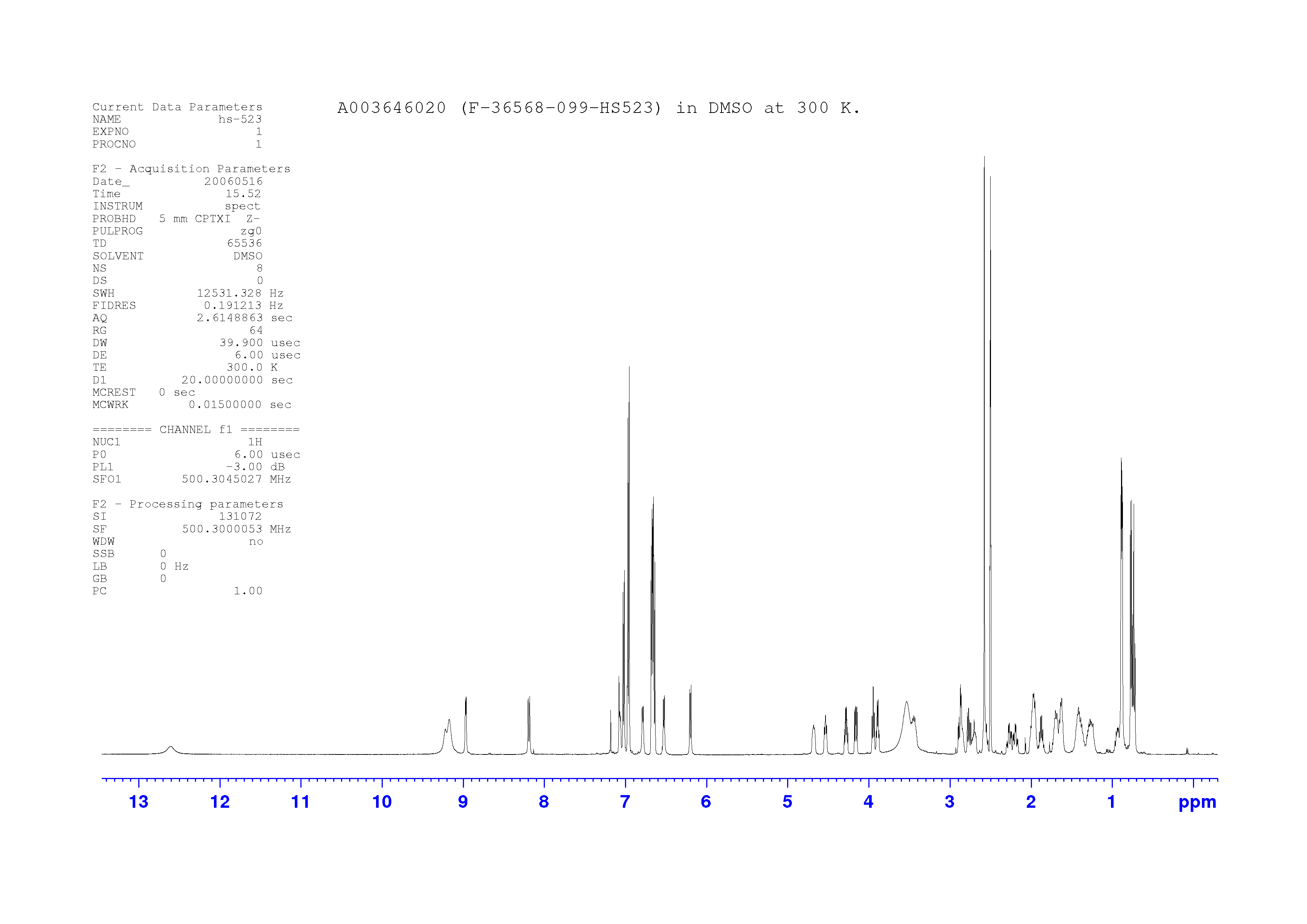


Fig. : ^1^H-spectrum of **15** in DMSO at 300 K.


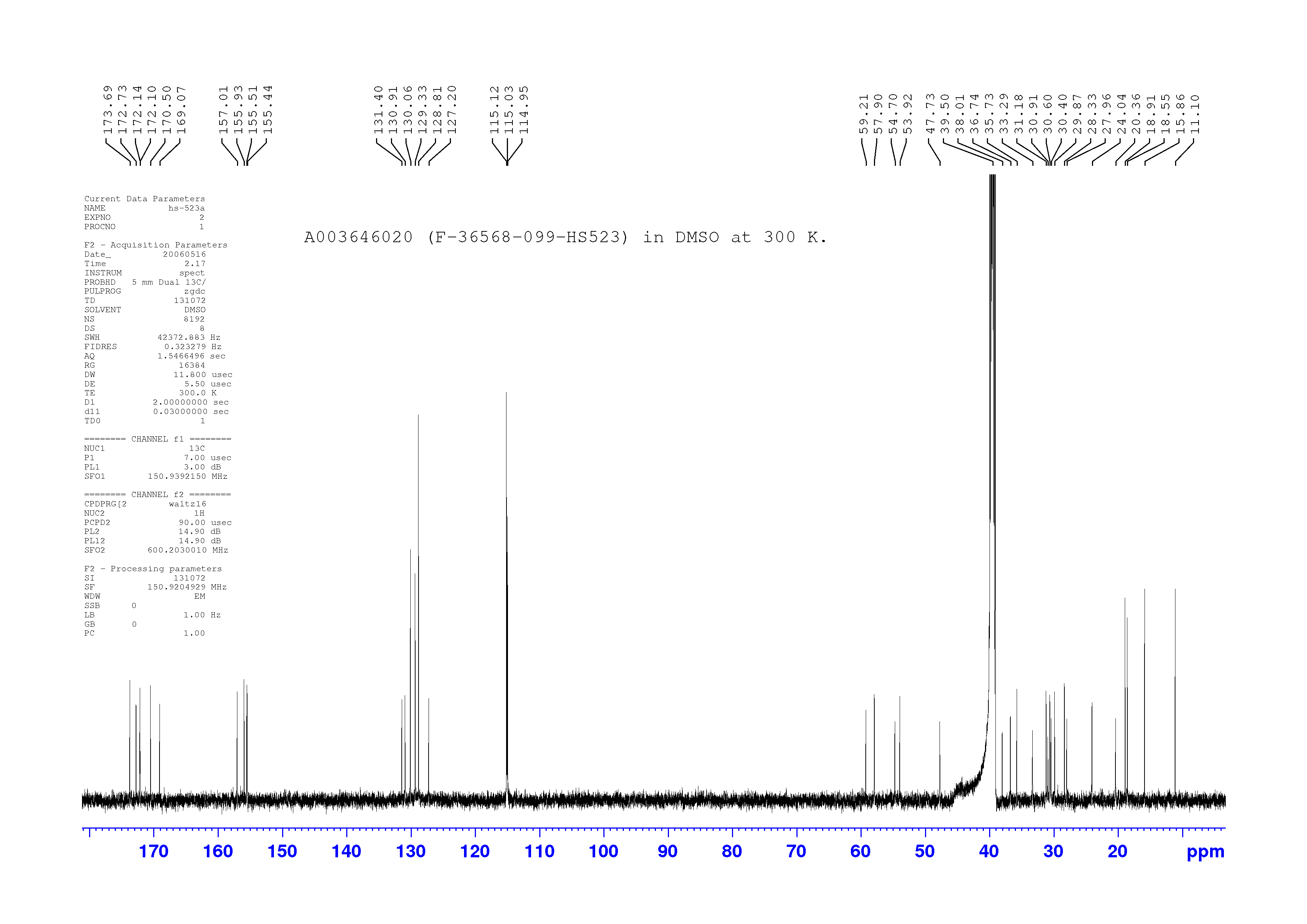


Fig. : ^13^C-spectrum of **15** in DMSO at 300 K.

Table 1: Chemical shifts of **15** in DMSO at 300 K.

|  | ^1^H | ^13^C |
| --- | --- | --- |
| Ile-1 NH | 8.19 | - |
| α | 4.16 | 57.90 |
| β | 1.95 | 35.73 |
| β-Me | 0.76 | 15.87 |
| γ | 1.29/0.93 | 24.04 |
| δ | 0.73 | 11.10 |
| C’ | - | 170.50 |
| HTy-2 NMe | 2.57 | 28.34 |
| α | 4.53 | 59.22 |
| β | 1.97/1.69 | 30.60 |
| homo-β | 2.27/2.19 | 31.18 |
| γ | - | 131.40 |
| δ | 6.96 | 128.81 |
| ε | 6.67 | 115.12 |
| ζ | - | 155.44 |
| ζ-OH | ~ 9.2 | - |
| C’ | - | 169.07 |
| HTy-3 NH | 8.97 | - |
| α | 4.67 | 47.73 |
| β | 1.98/1.68 | 33.29 |
| homo-β | 2.69/2.56 | 30.41 |
| γ | - | 130.91 |
| δ | 7.02 | 129.33 |
| ε | 6.64 | 115.03 |
| ζ | - | 155.51 |
| ζ-OH | ~ 9.2 | - |
| C’ | - | 172.10 |
| Val-4 NH | 6.79 | - |
| α | 3.94 | 57.92 |
| β | 1.87 | 29.87 |
| γ | 0.88 | 18.91 |
| γ’ | 0.88 | 18.55 |
| C’ | - | 172.74 |
| Lys-5 NH | 6.52 | - |
| α | 3.89 | 54.70 |
| β | 1.62 | 30.91 |
| γ | 1.38/1.24 | 20.36 |
| δ | 1.41 | 27.97 |
| ε | 3.44/2.85 | 38.01 |
| ζ-NH | 7.07 | - |
| C’ | - | 172.14 |
| Tyr-6 NH | 6.19 | - |
| α | 4.27 | 53.93 |
| β | 2.87/2.76 | 36.74 |
| γ | - | 127.21 |
| δ | 6.96 | 130.06 |
| ε | 6.66 | 114.95 |
| ζ | - | 155.93 |
| ζ-OH | ~ 9.2 | - |
| C’ | - | 173.69 |
| 1’-C’ | - | 157.01 |

Isolation of 9, 10, 11 and 12

Solid phase extraction of ATCC53789

Solid phase extraction was carried out in two identical steps, the first one using one third of the crude extract volume, the second one using the remaining two thirds of the extract volume. The crude extracts (~7L; ~14L) were loaded onto a column filled with CHP20-P (MCI® Gel, 75-150µ, Mitsubishi Chemical Corporation) material respectively.
For both parts a gradient with 0.1% formic acid : acetonitrile was applied (10% to 80% within 60 minutes, flow rate 250mL/min). Fractions were collected every minute. The fractions containing the compounds of interest were pooled and freeze-dried.

Isolation of **9**, **10**, **11** and **12**

Fractions 49, 50 and fraction 54 from solid phase extraction of ATCC53789 were separately purified whereas the combined fractions 45 and 46 were submitted to the next purification step. For all initial HPLC-separations the Varian Load & Lock Dynamic Axial Compression column filled with Phenomenex Luna C18(2) (dimension: 50x90mm, 10µm) equipped with an inline filter (dimension: 21x2mm, 10µm) was used. Compounds were eluted using a gradient of 0.1% formic acid : acetonitrile (for **9** /fraction 54 within 30 minutes from 27% to 40%, flow rate 140mL/min). The eluates were collected in ~40mL fractions (18sec) using UV-triggering (220nm).
The purification of fraction 54 yielded 45.5mg of **9**. From the separations of fractions 45 and 46, 49 and 50 the compounds **12** (14.4mg), **10** (3.2mg) and **11** (42.8mg) were obtained using comparable purification strategies. All compounds were freeze-dried and submitted for structure-elucidation and testing of biological activity.

**9**

UV: 206s, 219, 281 nm
C_45_H_58_ClN_7_O_9_, monoisotopic molecular mass (calc.): 875.398456 Da
Calc. [M+H^+^]: 876.4062 Da; observed [M+H^+^]: 876.409 Da

The analysis of the nmr spectra is hampered by the presence of two sets of signals in a ratio of ca. 1.3:1.0. As indicated by exchange NOEs in the ROESY spectrum these sets are caused by two different conformations. The two conformations originate from a different orientation of the amide bond between N-Me-Gly and HTy-3. In the main conformer the orientation is cis (strong ROE between N-Me-Gly-Hα and HTy-Hα), in the minor conformer the orientation is trans. This compound has not been described in literature but is related to *Anabaenopeptins NZ825*, *NZ841* and *NZ857*^[[3]](#footnote-3)^.

Fig. 1 Structure of **9**


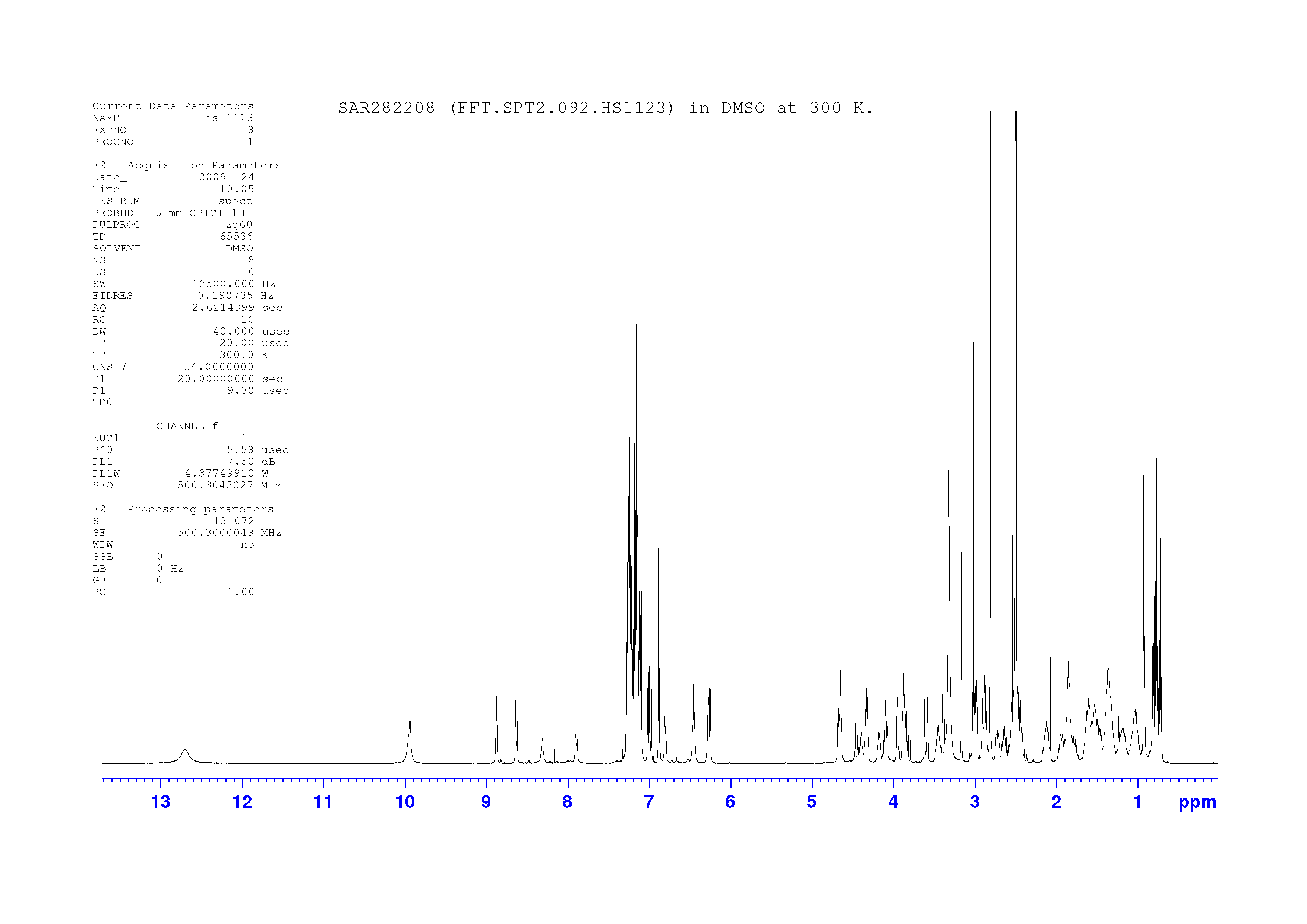


Fig.: ^1^H NMR Spectrum of **9** in DMSO at 300K


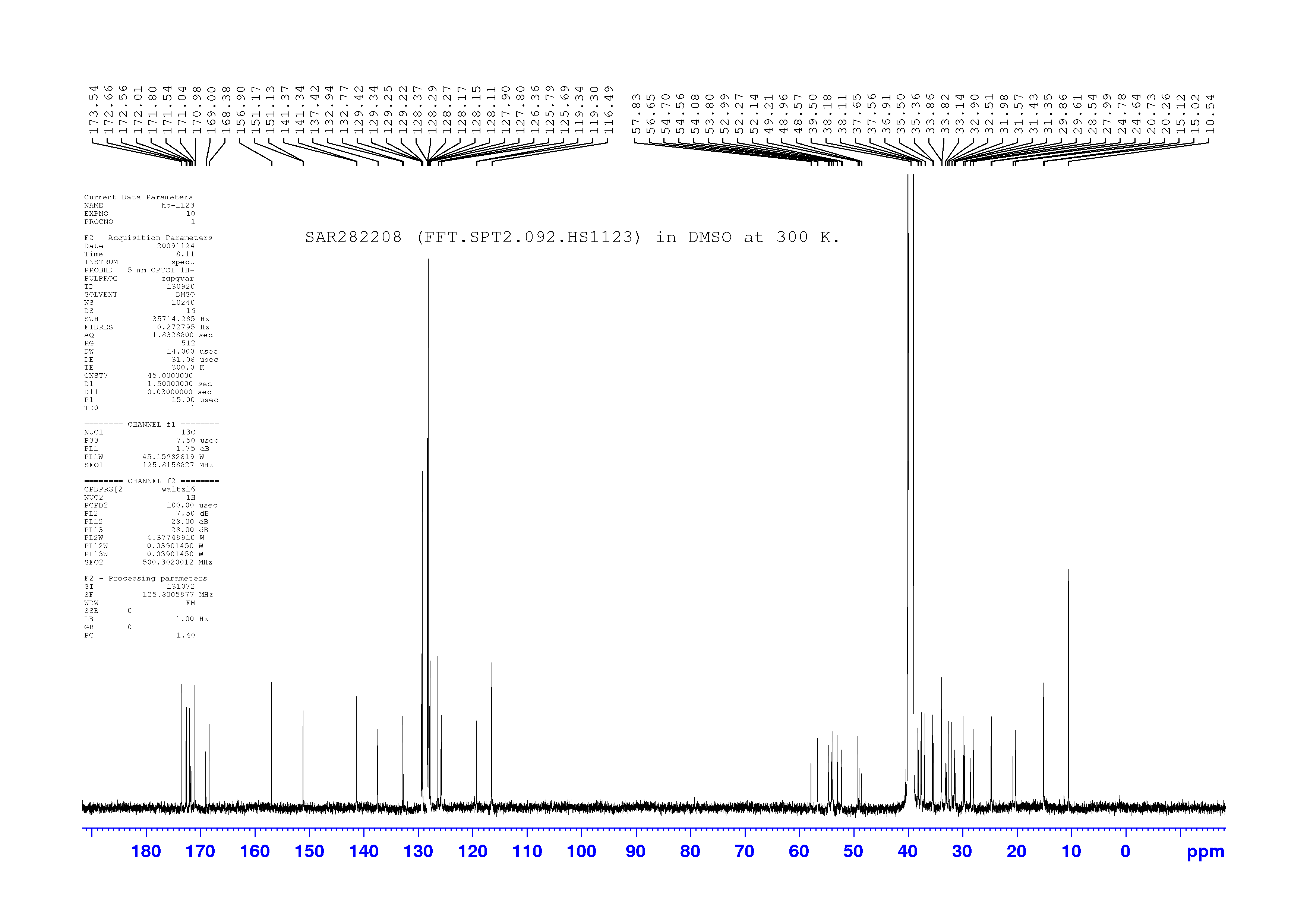


Fig.: ^13^C NMR Spectrum of **9** in DMSO at 300K

Table 1: Chemical shifts of **9** in DMSO at 300 K.

|  | ^1^H | | ^13^C | |
| --- | --- | --- | --- | --- |
|  | cis | trans | cis | trans |
| HPh-1 NH | 8.63 | 7.90 | - | - |
| α | 4.10 | 4.18 | 52.99 | 52.27 |
| β | 2.13/1.94 | 2.11/1.78 | 33.86 | 32.90 |
| homo-β | 2.48 | 2.53/2.44 | 31.98 | 31.57 |
| γ | - | - | 141.37 | 141.34 |
| δ | 7.11 | 7.13 | 128.29 (a) | 128.27 (a) |
| ε | 7.24 | 7.24 | 128.15 (b) | 128.17 (b) |
| ζ | 7.16 | 7.16 | 125.79 | 125.69 |
| C’ | - | - | 170.98 | 170.97 |
| N-Me-Gly-2 NMe | 2.81 | 3.02 | 33.82 | 36.91 |
| α | 4.67/3.60 | 4.46/3.39 | 54.08 | 52.14 |
| C’ | - | - | 169.00 | 168.38 |
| HTy-3 NH | 8.88 | 8.31 | - | - |
| α | 4.66 | 4.40 | 49.21 | 48.96 |
| β | 1.85 | 1.85 | 32.51 | 33.14 |
| homo-β | 2.65/2.52 | 2.56/2.50 | 29.86 | 29.61 |
| 1’ | - | - | 119.34 | 119.30 |
| 2’ | - | - | 151.13 | 151.17 |
| 3’ | 6.88 | 6.88 | 116.49 | 116.51 |
| 4’ | 7.01 | 6.98 | 127.80 | 127.90 |
| 5’ | - | - | 132.94 | 132.77 |
| 6’ | 7.23 | 7.18 | 129.34 | 129.42 |
| C’ | - | - | 171.04 | 171.54 |
| Ile-4 NH | 6.80 | 7.11 | - | - |
| α | 3.95 | 3.84 | 56.65 | 57.83 |
| β | 1.60 | 1.65 | 35.50 | 35.36 |
| β-Me | 0.93 | 0.81 | 15.02 | 15.12 |
| γ | 1.47/1.02 | 1.39/1.02 | 24.64 | 24.78 |
| δ | 0.77 | 0.72 | 10.52 | 10.54 |
| C’ | - | - | 172.56 | 171.80 |
| Lys-5 NH | 6.45 | 6.46 | - | - |
| α | 3.88 | 3.88 | 54.56 | 54.70 |
| β | 1.54 | 1.60/1.52 | 31.43 | 31.35 |
| γ | 1.17/1.06 | 1.35/1.21 | 20.26 | 20.73 |
| δ | 1.34 | 1.37 | 27.99 | 28.54 |
| ε | 3.46/2.73 | 3.32/2.90 | 38.18 | 38.11 |
| ζ-NH | 7.23 | 7.12 | - | - |
| C’ | - | - | 172.01 | 172.66 |
| Phe-6 NH | 6.26 | 6.28 | - | - |
| α | 4.32 | 4.34 | 53.80 | 53.80 |
| β | 2.99/2.86 | 3.00/2.88 | 37.56 | 37.65 |
| γ | - | - | 137.42 | 137.46 |
| δ | 7.16 | 7.17 | 129.22 | 129.25 |
| ε | 7.26 | 7.26 | 128.11 | 128.11 |
| ζ | 7.20 | 7.20 | 126.36 | 126.36 |
| C’ | - | - | 173.54 | 173.54 |
| 1’ | - | - | 156.90 | 156.90 |

1. May be interchanged.
2. May be interchanged.

**10**

UV: 202, 218s, 279 nm
C_44_H_56_ClN_7_O_10_, monoisotopic molecular mass (calc.): 877.3777 Da
Calc. [M+H^+^]: 878.3855 Da; observed [M+H^+^]: 878.386 Da

Fig. 1 Structure of **10**


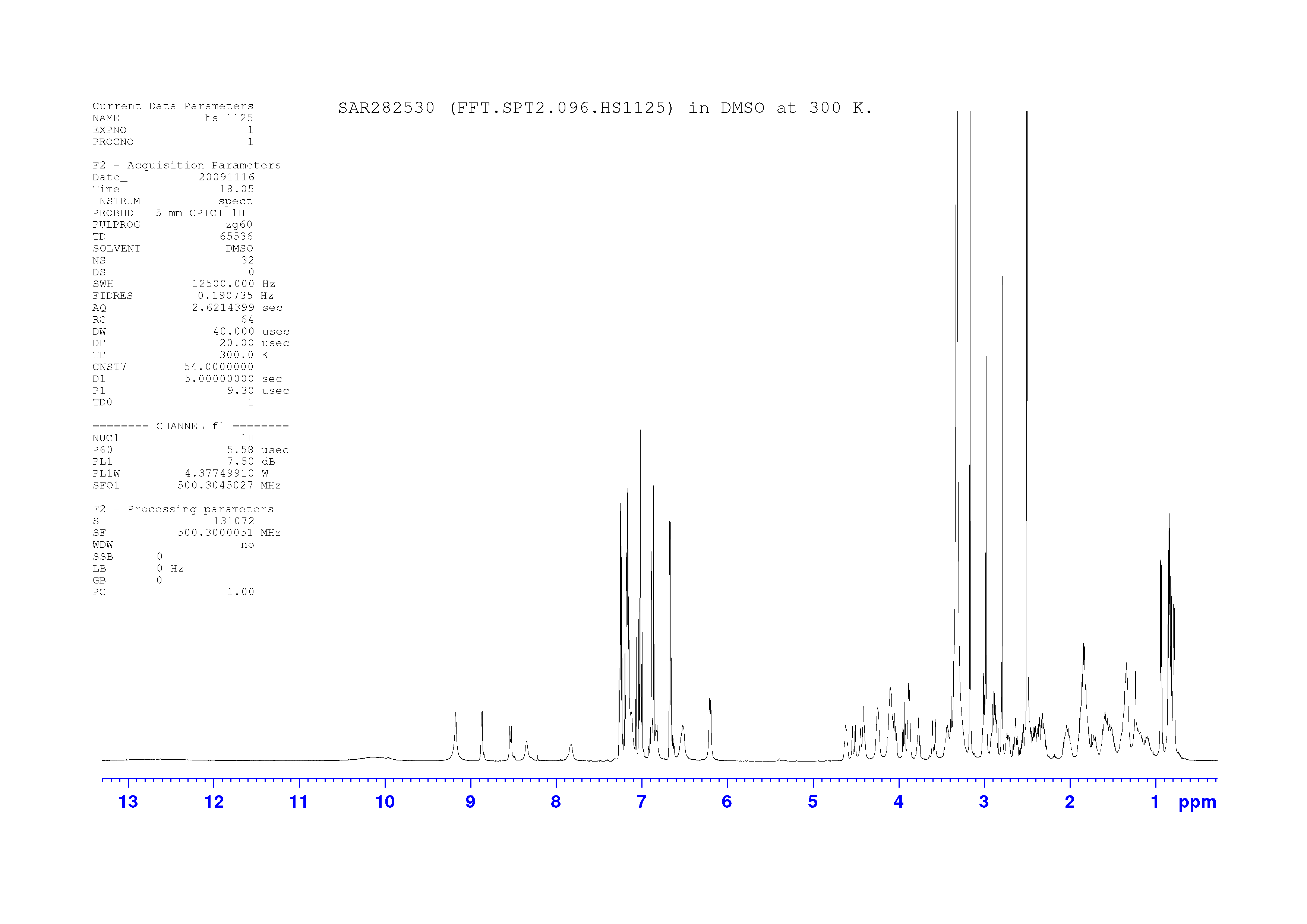


Fig.: ^1^H NMR Spectrum of **10** in DMSO at 300K.


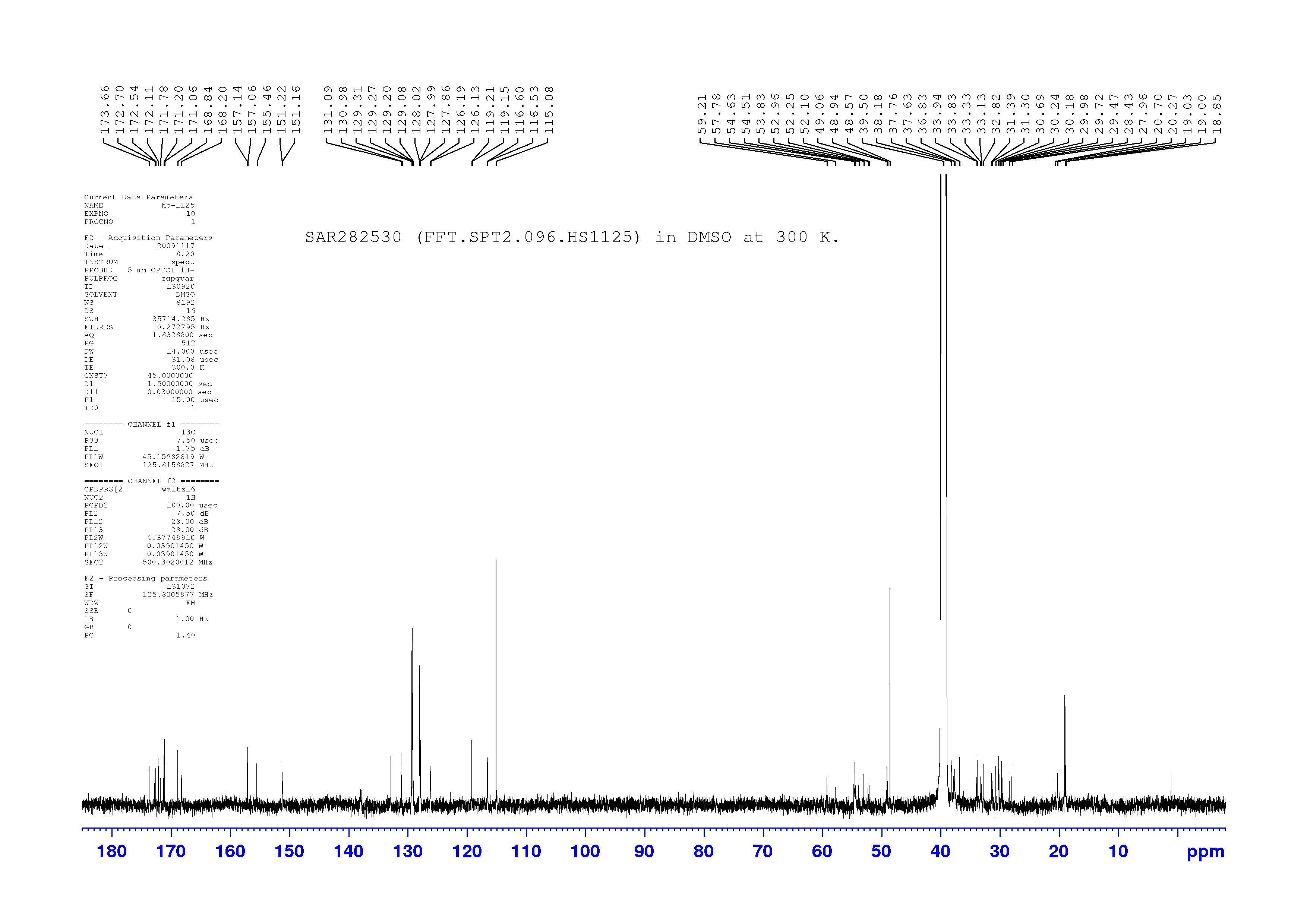


Fig.: ^13^C NMR Spectrum of **10** in DMSO at 300K.

Table 1: Chemical shifts of **10** in DMSO at 300 K.

|  | ^1^H | | ^13^C | |
| --- | --- | --- | --- | --- |
|  | cis | trans | cis | trans |
| HTy-1 NH | 8.53 | 7.83 | - | - |
| α | 4.05 | 4.11 | 52.97 | 52.24 |
| β | 2.05/1.86 | 2.01/1.71 | 33.94 | 33.14 |
| homo-β | 2.38/2.32 | 2.43/2.30 | 30.69 | 30.24 |
| 1’ | - | - | 119.21 | 119.15 |
| 2’ | - | - | 151.22 | 151.16 |
| 3’ | 6.86 | 6.89 | 116.60 | 116.53 |
| 4’ | 6.86 | 6.89 | 127.83 | 127.86 |
| 5’ | - | - | 132.83 | 132.88 |
| 6’ | 7.02 | 7.07 | 129.20 | 129.20 |
| C’ | - | - | 171.06 | 171.06 |
| N-Me-Gly-2 NMe | 2.79 | 2.98 | 33.83 | 36.83 |
| α | 4.53/3.59 | 4.43/3.37 | 53.82 | 52.10 |
| C’ | - | - | 168.84 | 168.20 |
| HTy-3 NH | 8.87 | 8.35 | - | - |
| α | 4.62 | 4.41 | 49.06 | 48.94 |
| β | 1.87/1.81 | 1.84 | 32.82 | 33.33 |
| homo-β | 2.63/2.49 | 2.55/2.48 | 30.18 | 29.98 |
| γ | - | - | 131.09 | 130.98 |
| δ | 7.03 | 7.01 | 129.08 | 129.20 |
| ε | 6.67 | 6.67 | 115.08 | 115.08 |
| ζ | - | - | 155.47 | 155.48 |
| ζ-OH | ~ 10.1 (broad) | ~ 10.1 (broad) | - | - |
| C’ | - | - | 171.20 | 171.82 |
| Val-4 NH | 6.83 | 7.11 | - | - |
| α | 3.93 | 3.77 | 57.79 | 59.21 |
| β | 1.84 | 1.80 | 29.72 | 29.47 |
| γ | 0.94 | 0.82 | 19.03 | 19.00 |
| γ’ | 0.85 | 0.79 | 18.85 | 19.00 |
| C’ | - | - | 172.54 | 171.78 |
| Lys-5 NH | 6.51 | 6.54 | - | - |
| α | 3.88 | 3.88 | 54.51 | 54.63 |
| β | 1.60/1.54 | 1.59/1.51 | 31.29 | 31.39 |
| γ | 1.17/1.10 | 1.34/1.23 | 20.27 | 20.70 |
| δ | 1.34 | 1.36 | 27.96 | 28.43 |
| ε | 3.44/2.73 | 3.27/2.90 | 38.19 | 38.23 |
| ζ-NH | 7.15 | 7.12 | - | - |
| C’ | - | - | 172.11 | 172.70 |
| Phe-6 NH | 6.20 | 6.20 | - | - |
| α | 4.24 | 4.24 | ~ 54.4 (broad) | ~ 54.4 (broad) |
| β | 2.99/2.87 | 3.00/2.89 | 37.63 | 37.76 |
| γ | - | - | ~ 137.8 (broad) | ~ 137.8 (broad) |
| δ | 7.17 | 7.17 | 129.26 | 129.31 |
| ε | 7.25 | 7.25 | 128.02 | 127.99 |
| ζ | 7.18 | 7.18 | 126.19 | 126.13 |
| C’ | - | - | 173.66 | 173.66 |
| 1’ | - | - | 157.06 | 157.14 |

**11**

UV: 204,217s, 280 nm
C_45_H_58_ClN_7_O_10_, monoisotopic molecular mass (calc.): 891.3934 Da
Calc. [M+H^+^]: 892.4012 Da; observed [M+H^+^]: 892.402 Da

Fig. 1 Structure of **11**


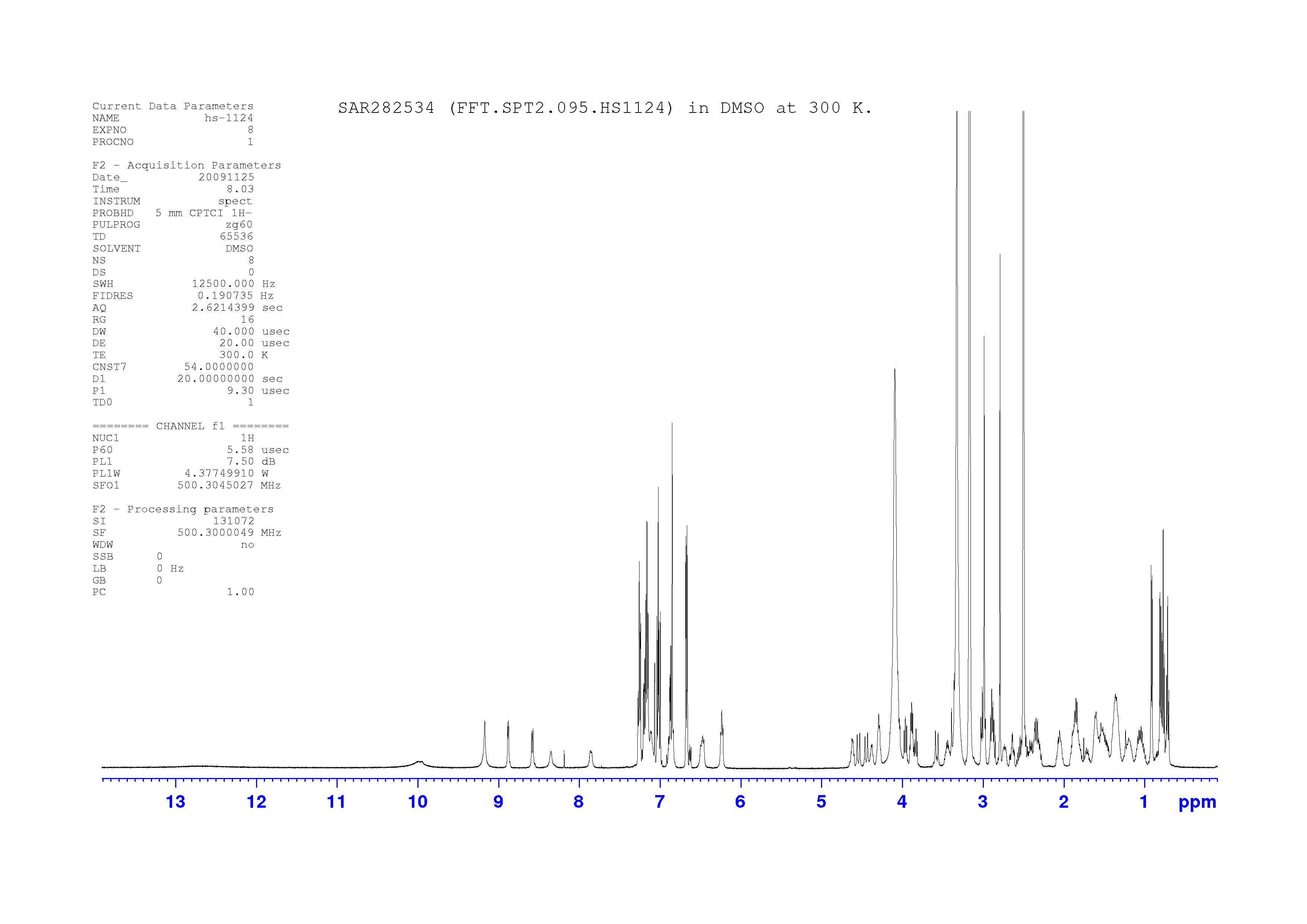


Fig.: ^1^H NMR Spectrum of **11** in DMSO at 300K


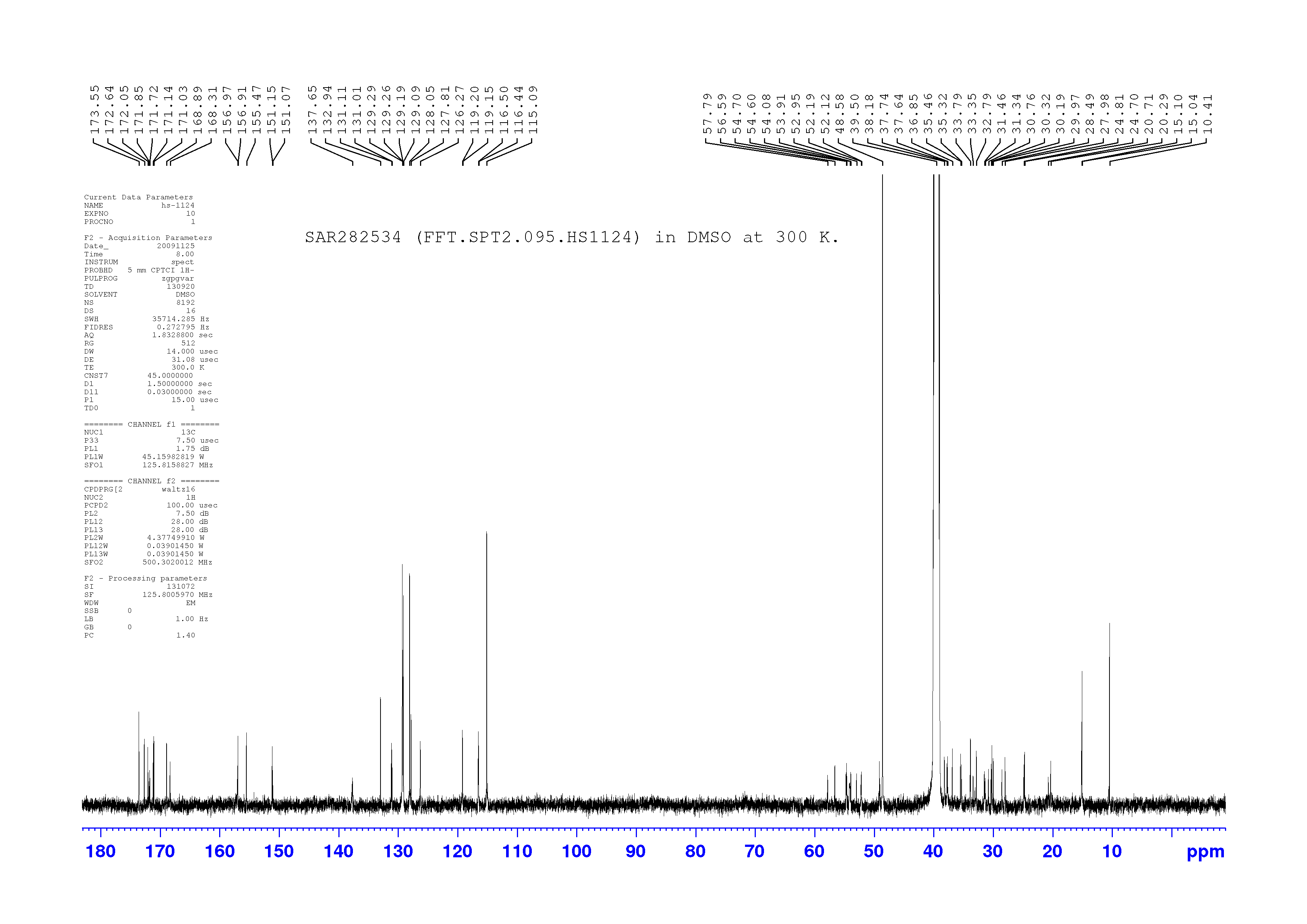


Fig.: ^13^C NMR Spectrum of **11** in DMSO at 300K

Table 1: Chemical shifts of **11**

|  | ^1^H | | ^13^C | |
| --- | --- | --- | --- | --- |
|  | cis | trans | cis | trans |
| HTy-1 NH | 8.58 | 7.86 | - | - |
| α | 4.05 | 4.13 | 52.95 | 52.19 |
| β | 2.06/1.88 | 2.04/1.71 | 33.86 | 33.10 |
| homo-β | 2.35 | 2.42/2.31 | 30.76 | 30.32 |
| 1’ | - | - | 119.20 | 119.15 |
| 2’ | - | - | 151.15 | 151.07 |
| 3’ | 6.86 | 6.86 | 116.50 | 116.44 |
| 4’ | 6.86 | 6.88 | 127.81 | 127.82 |
| 5’ | - | - | 132.94 | 132.94 |
| 6’ | 7.02 | 7.07 | 129.29 | 129.29 |
| C’ | - | - | 171.03 | 171.01 |
| N-Me-Gly-2 NMe | 2.79 | 2.99 | 33.79 | 36.85 |
| α | 4.54/3.57 | 4.46/3.37 | 53.91 | 52.12 |
| C’ | - | - | 168.89 | 168.31 |
| HTy-3 NH | 8.88 | 8.35 | - | - |
| α | 4.62 | 4.38 | 49.09 | 49.03 |
| β | 1.88/1.81 | 1.84 | 32.79 | 33.35 |
| homo-β | 2.64/2.50 | 2.55/2.48 | 30.18 | 29.97 |
| γ | - | - | 131.11 | 131.01 |
| δ | 7.03 | 7.00 | 129.09 | 129.19 |
| ε | 6.67 | 6.67 | 115.09 | 115.09 |
| ζ | - | - | 155.47 | 155.47 |
| ζ-OH | ~ 10.0 (broad) | ~ 10.0 (broad) | - | - |
| C’ | - | - | 171.14 | 171.85 |
| Ile-4 NH | 6.84 | 7.10 | - | - |
| α | 3.96 | 3.83 | 56.59 | 57.79 |
| β | 1.61 | 1.59 | 35.46 | 35.32 |
| β-Me | 0.91 | 0.80 | 15.04 | 15.10 |
| γ | 1.47/1.05 | 1.38/1.02 | 24.70 | 24.81 |
| δ | 0.77 | 0.71 | 10.41 | 10.41 |
| C’ | - | - | 172.64 | 171.85 |
| Lys-5 NH | 6.46 | 6.49 | - | - |
| α | 3.89 | 3.88 | 54.60 | 54.70 |
| β | 1.54 | 1.61/1.52 | 31.46 | 31.33 |
| γ | 1.18/1.08 | 1.34/1.20 | 20.29 | 20.71 |
| δ | 1.35 | 1.36 | 27.98 | 28.49 |
| ε | 3.45/2.73 | 3.30/2.90 | 38.18 | 38.14 |
| ζ-NH | 7.15 | 7.12 | - | - |
| C’ | - | - | 172.05 | 172.65 |
| Phe-6 NH | 6.23 | 6.24 | - | - |
| α | 4.29 | 4.29 | ~ 54.1 (broad) | ~ 54.1 (broad) |
| β | 2.99/2.88 | 2.99/2.88 | 37.64 | 37.74 |
| γ | - | - | ~ 137.6 (broad) | ~ 137.7 (broad) |
| δ | 7.15 | 7.17 | 129.26 | 129.29 |
| ε | 7.26 | 7.26 | 128.05 | 128.05 |
| ζ | 7.19 | 7.19 | 126.27 | 126.27 |
| C’ | - | - | 173.55 | 173.55 |
| 1’ | - | - | 156.91 | 156.97 |

**12**

UV: 220s, 277 nm
C_44_H_57_N_7_O_10_, monoisotopic molecular mass (calc.): 843.4167 Da
Calc. [M+H^+^]: 844.4245 Da; observed [M+H^+^]: 844.425 Da

Fig. 1 Structure of **12**


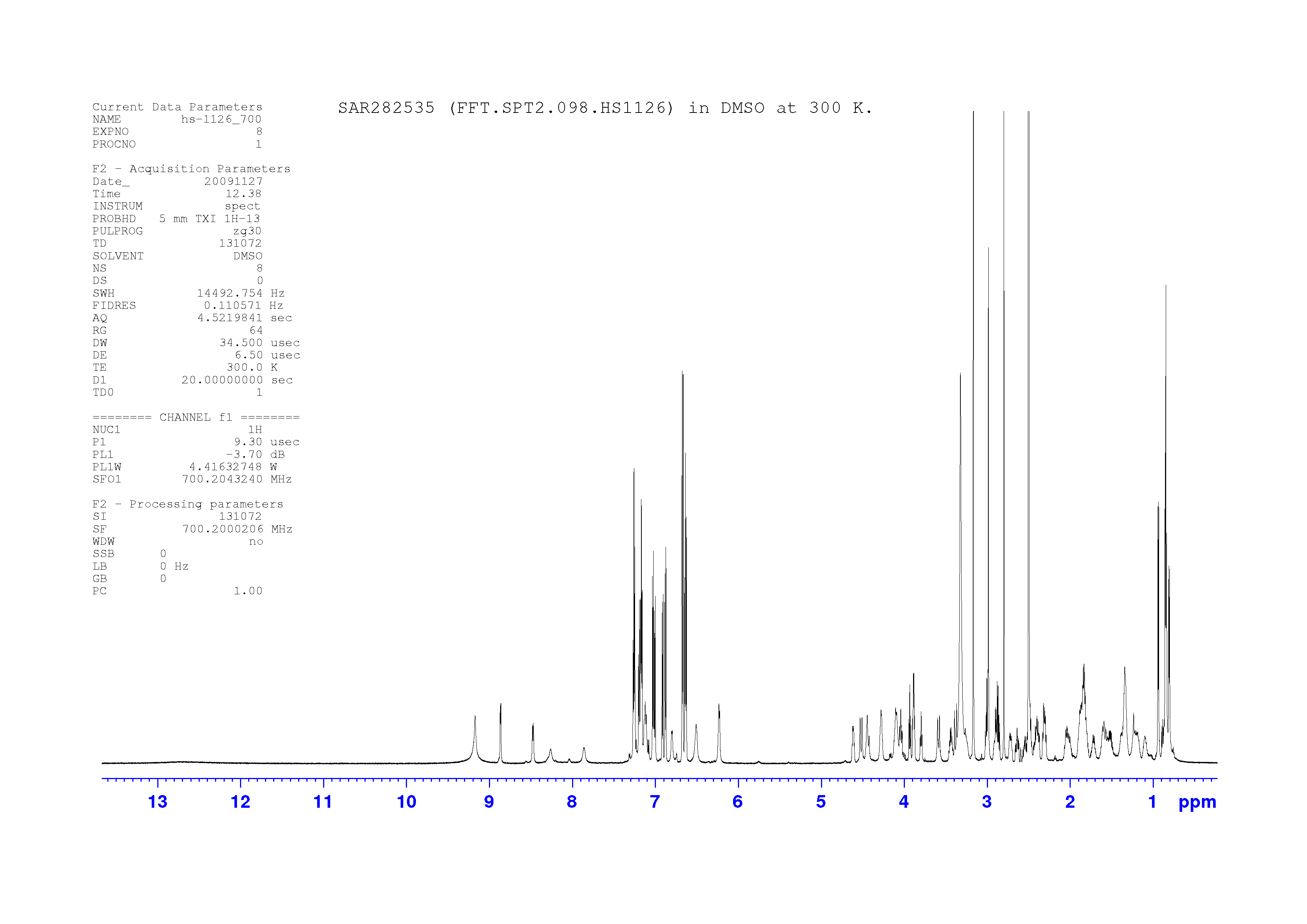


Fig.: ^1^H NMR Spectrum of **12** in DMSO at 300K


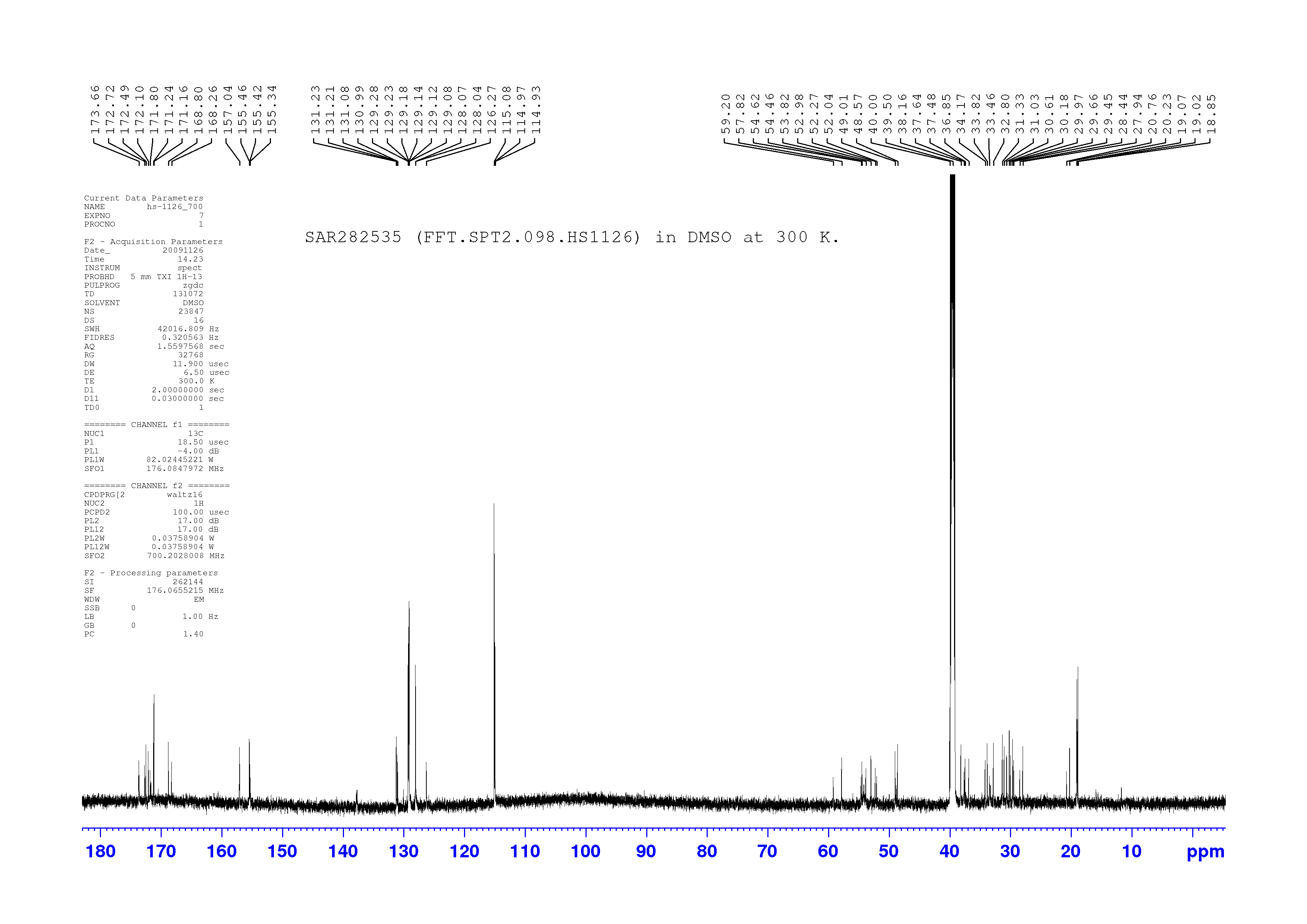


Fig.: ^13^C NMR Spectrum of **12** in DMSO at 300K

Table 1: Chemical shifts of **12** in DMSO at 300 K.

|  | ^1^H | | ^13^C | |
| --- | --- | --- | --- | --- |
|  | major | minor | major | minor |
| HTy-1 NH | 8.47 | 7.86 | - | - |
| α | 4.04 | 4.11 | 52.98 | 52.27 |
| β | 2.04/1.85 | 2.01/1.72 | 34.17 | 33.25 |
| homo-β | 2.38/2.31 | 2.42/2.31 | 31.03 | 30.61 |
| γ | - | - | 131.21 | 131.23 |
| δ | 6.88 | 6.91 | 129.14 | 129.12 |
| ε | 6.63 | 6.64 | 114.97 | 114.93 |
| ζ | - | - | 155.42 | 155.34 |
| C’ | - | - | 171.16 | 171.16 |
| N-Me-Gly-2 NMe | 2.80 | 2.98 | 33.82 | 36.85 |
| α | 4.52/3.59 | 4.44/3.38 | 53.82 | 52.04 |
| C’ | - | - | 168.80 | 168.26 |
| HTy-3 NH | 8.87 | 8.26 | - | - |
| α | 4.61 | 4.45 | 49.01 | 48.82 |
| β | 1.87/1.80 | 1.84 | 32.80 | 33.46 |
| homo-β | 2.64/2.50 | 2.54/2.49 | 30.18 | 29.97 |
| γ | - | - | 131.08 | 130.99 |
| δ | 7.03 | 7.01 | 129.08 | 129.18 |
| ε | 6.67 | 6.67 | 115.08 | 115.08 |
| ζ | - | - | 155.46 | 155.46 |
| ζ-OH | 9.18 | 9.18 | - | - |
| C’ | - | - | 171.24 | 171.65 |
| Val-4 NH | 6.80 | 7.19 | - | - |
| α | 3.93 | 3.80 | 57.82 | 59.20 |
| β | 1.83 | 1.88 | 29.66 | 29.45 |
| γ | 0.94 | 0.84 | 19.02 | 19.07 |
| γ’ | 0.85 | 0.81 | 18.85 | 18.85 |
| C’ | - | - | 172.49 | 171.65 |
| Lys-5 NH | 6.50 | 6.52 | - | - |
| α | 3.88 | 3.88 | 54.46 | 54.62 |
| β | 1.59/1.52 | 1.61/1.55 | 31.33 | 31.33 |
| γ | 1.18/1.09 | 1.32/1.22 | 20.23 | 20.76 |
| δ | 1.34 | 1.37/1.33 | 27.94 | 28.44 |
| ε | 3.44/2.72 | 3.26/2.90 | 38.16 | 38.21 |
| ζ-NH | 7.13 | 7.11 | - | - |
| C’ | - | - | 172.10 | 172.72 |
| Phe-6 NH | 6.23 | 6.23 | - | - |
| α | 4.28 | 4.28 | ~ 54.2 | ~ 54.2 |
| β | 2.99/2.86 | 3.01/2.88 | 37.48 | 37.64 |
| γ | - | - | ~ 137.6 | ~ 137.6 |
| δ | 7.16 | 7.17 | 129.23 | 129.28 |
| ε | 7.26 | 7.26 | 128.07 | 128.04 |
| ζ | 7.19 | 7.19 | 126.27 | 126.22 |
| C’ | - | - | 173.66 | 173.61 |
| 1’ | - | - | 157.04 | 157.04 |

### Isolation of 4 and 6

Solid phase extraction of CBT163

The crude extract (~6L) consisting of a water : methanol 1:1 mixture was loaded onto a column filled with ~5.8L of CHP20-P (MCI® Gel, 75-150µ, Mitsubishi Chemical Corporation) material.
A gradient with ammonium acetate (50g NH_4_Ac / L H_2_O adjusted to pH 4.6 with acetic acid) : acetonitrile was applied (10% to 50% within 25 minutes, then keep 50% for 10 minutes followed by 50% to 100% within 25 minutes, flow rate 120mL/min). Fractions were collected over 2 minutes. The fractions containing the compounds of interest were pooled, freeze-dried and further purified.

Isolation of **4** and **6**

Fractions 22-26 from solid phase extraction were purified using a Phenomenex Luna C18(2) column (dimension: 21x250mm, 10µm) combined with a Waters X-Terra pre-column (dimension: 19x10mm, 10µm). Compounds were eluted using a gradient of ammonium acetate (50g NH_4_Ac /L H_2_O adjusted to pH 7.0) : acetonitrile (15% to 50% within 45 minutes, flow rate 60mL/min). The eluates were collected by UV-triggering (210nm) over 30 sec (30mL). In order to prevent column-overloading the freeze-dried material was split into two portions and the separation just described was carried out in two identical steps.
The pre-purified fractions were further purified using a Waters X-Bridge column (dimension: 19x100mm, 5µm) with a Waters X-Bridge pre-column (dimension: 19x10mm, 10µm) applying a gradient of 0.1% trifluoroacetic acid : acetonitrile (20% to 40% within 30 minutes, flow rate 40ml/min). The fractions were collected by UV-triggering (210nm) over 12 sec (~8mL). Again this separation is carried out with two portions of the freeze-dried material in an identical mode in order to prevent column overload. After freeze-drying **4** (24.3mg) and **6** (4.1mg) were obtained in good purity.

**4**

UV: end

C_44_H_65_N_9_O_9_, monoisotopic molecular mass (calc.): 863.4905 Da
Calc. [M+H^+^]: 864.4984 Da; observed [M+H^+^]: 864.4988 Da

Fig. 1 Structure of **4**


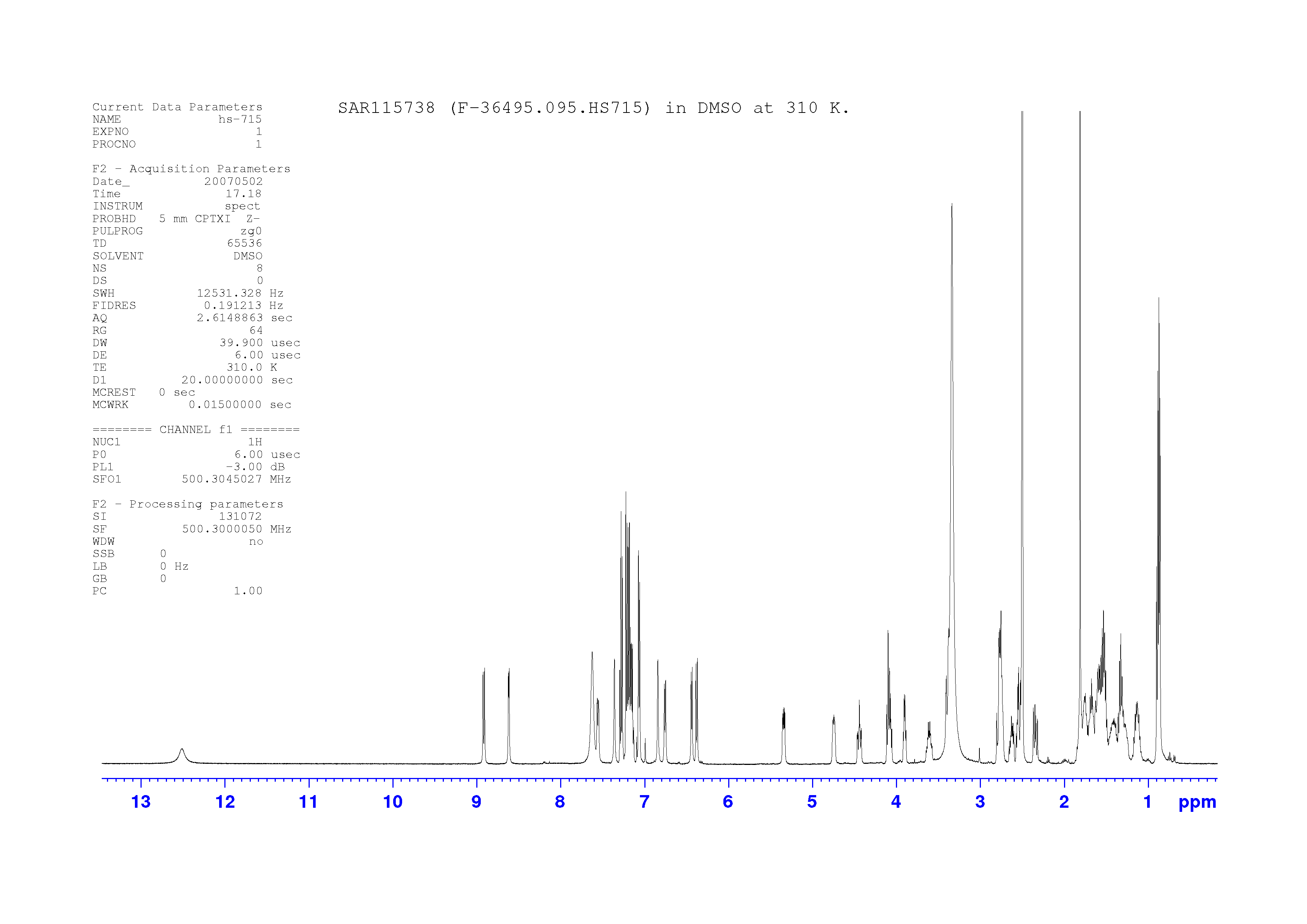


Fig.: ^1^H NMR Spectrum of **4** in DMSO at 310K


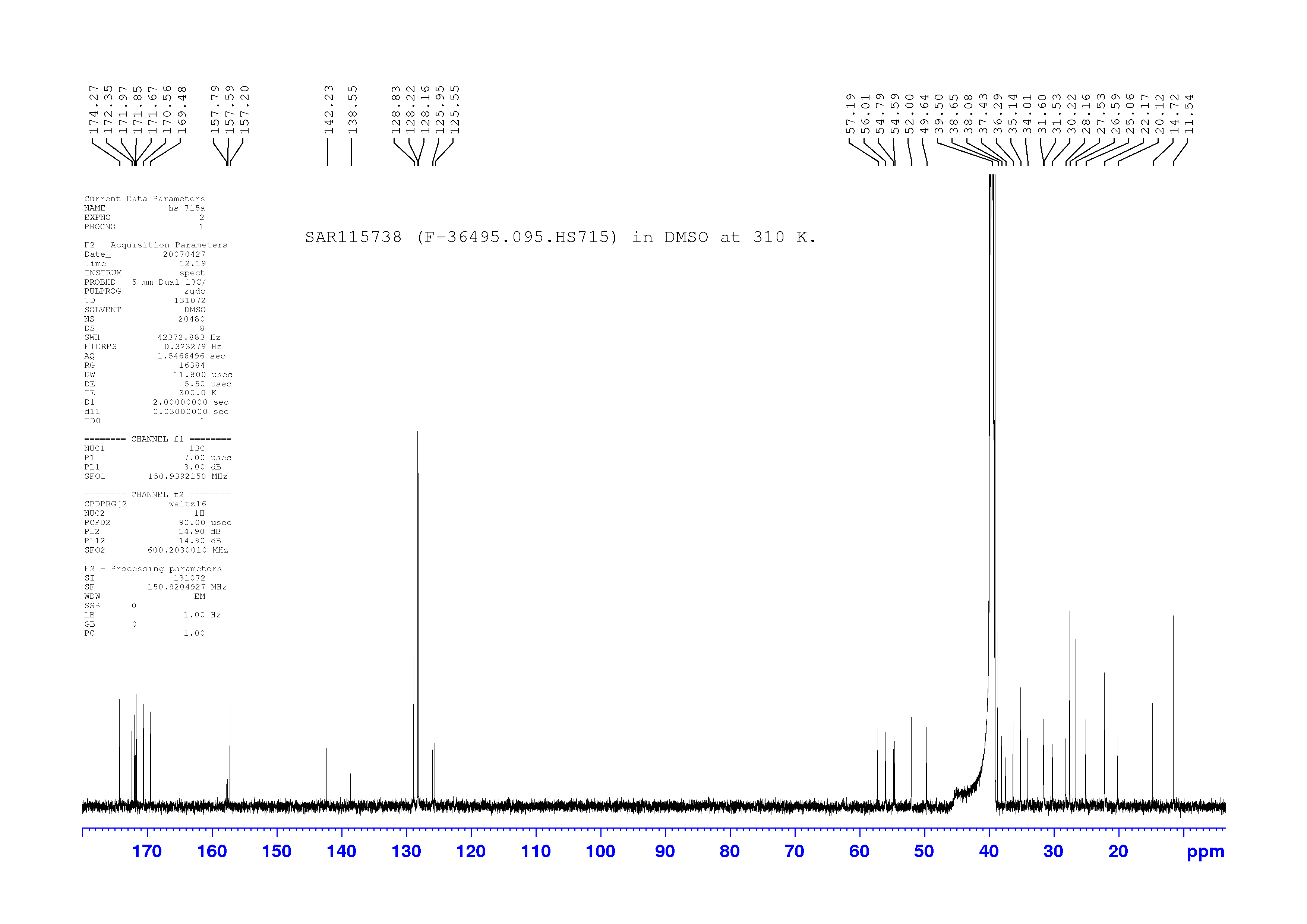


Fig.: ^13^C NMR Spectrum of **4** in DMSO at 300K

Table 1: Chemical shifts of **4** in DMSO at 300/310 K^[[4]](#footnote-4)^.

|  | ^1^H | ^13^C |
| --- | --- | --- |
| Phe-1 NH | 8.92 | - |
| α | 4.44 | 54.79 |
| β | 3.40/2.78 | 37.44 |
| γ | - | 138.55 |
| δ | 7.07 | 128.83 |
| ε | 7.18 | 128.16 |
| ζ | 7.14 | 125.95 |
| C’ | - | 170.56 |
| N-Me-Asn-2 NMe | 1.81 | 27.54 |
| α | 5.34 | 57.19 |
| β | 2.53/2.35 | 34.01 |
| γ | - | 171.67 |
| NH2 | 7.36/6.84 | - |
| C’ | - | 169.78 |
| APPA-3 NH | 8.62 | - |
| α | 4.74 | 49.64 |
| β | 1.82/1.60 | 30.22 |
| Β’ | 1.75/1.68 | 27.53 |
| Β’’ | 2.62/2.55 | 35.15 |
| γ | - | 142.24 |
| δ | 7.22 | 128.22 |
| ε | 7.28 | 128.16 |
| ζ | 7.17 | 125.55 |
| C’ | - | 171.97 |
| Ile-4 NH | 6.76 | - |
| α | 4.10 | 56.01 |
| β | 1.76 | 36.29 |
| β-Me | 0.86 | 14.72 |
| γ | 1.58/1.14 | 25.06 |
| δ | 0.88 | 11.54 |
| C’ | - | 172.35 |
| Lys-5 NH | 6.44 | - |
| α | 3.90 | 54.59 |
| β | 1.63/1.54 | 31.60 |
| γ | 1.28/1.13 | 20.13 |
| δ | 1.42 | 28.16 |
| ε | 3.61/2.75 | 38.09 |
| ζ-NH | 7.56 | - |
| C’ | - | 171.85 |
| Lys-6 NH | 6.38 | - |
| α | 4.07 | 52.00 |
| β | 1.65/1.52 | 31.53 |
| γ | 1.33 | 22.17 |
| δ | 1.53 | 26.59 |
| ε | 2.76 | 38.65 |
| ζ-NH_3_^+^ | 7.62 | - |
| C’ | - | 174.27 |
| 1’-C’ | - | 157.20 |

APPA=2-Amino-5-phenylpentanoic acid

**6**

UV: end

C_43_H_62_N_8_O_9_, monoisotopic molecular mass (calc.): 834.4640 Da
Calc. [M+H^+^]: 835.4718 Da; observed [M+H^+^]: 835.4735 Da

Fig. 1 Structure of **6**


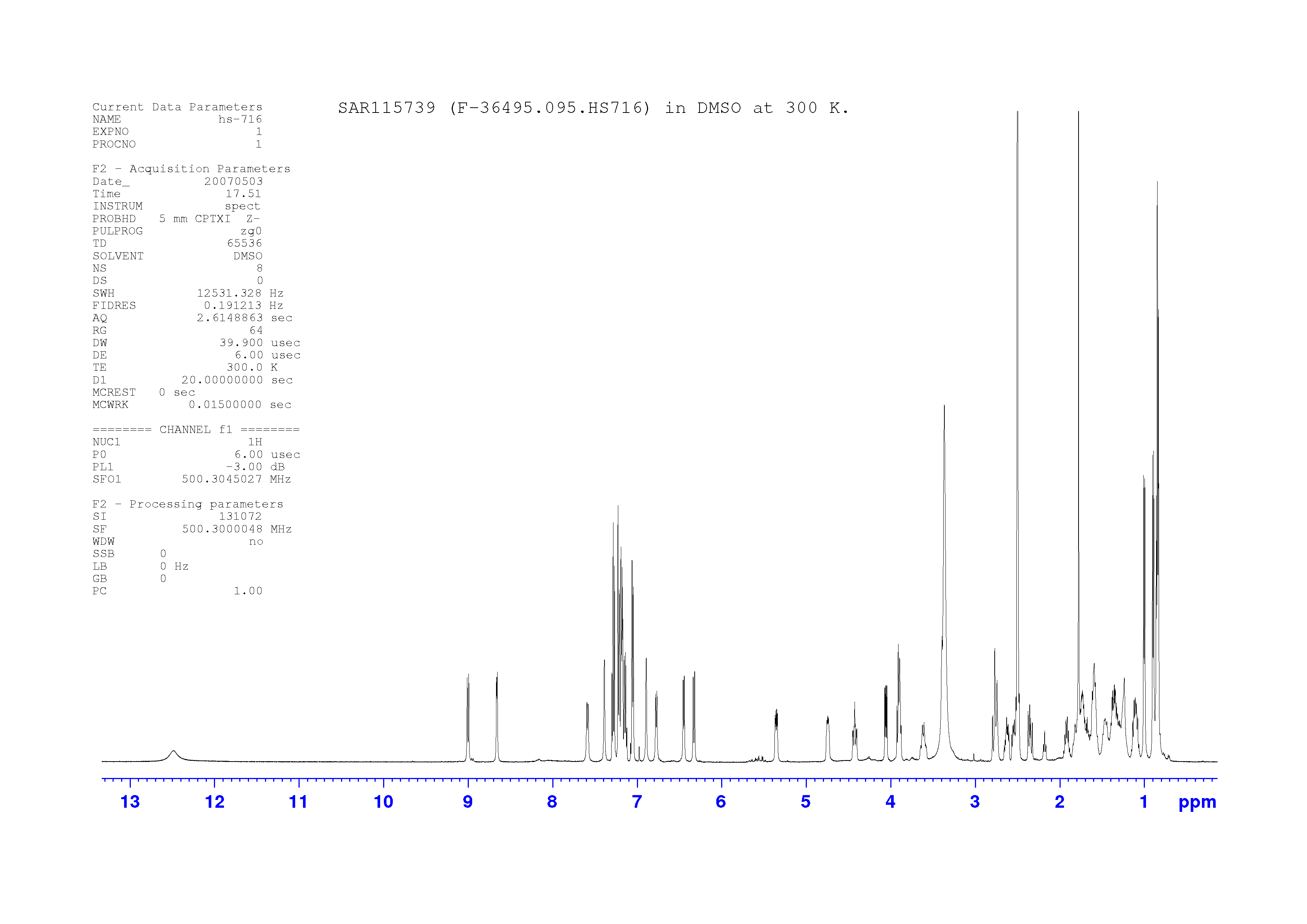


Fig. : ^1^H-spectrum of **6** in DMSO at 300 K.


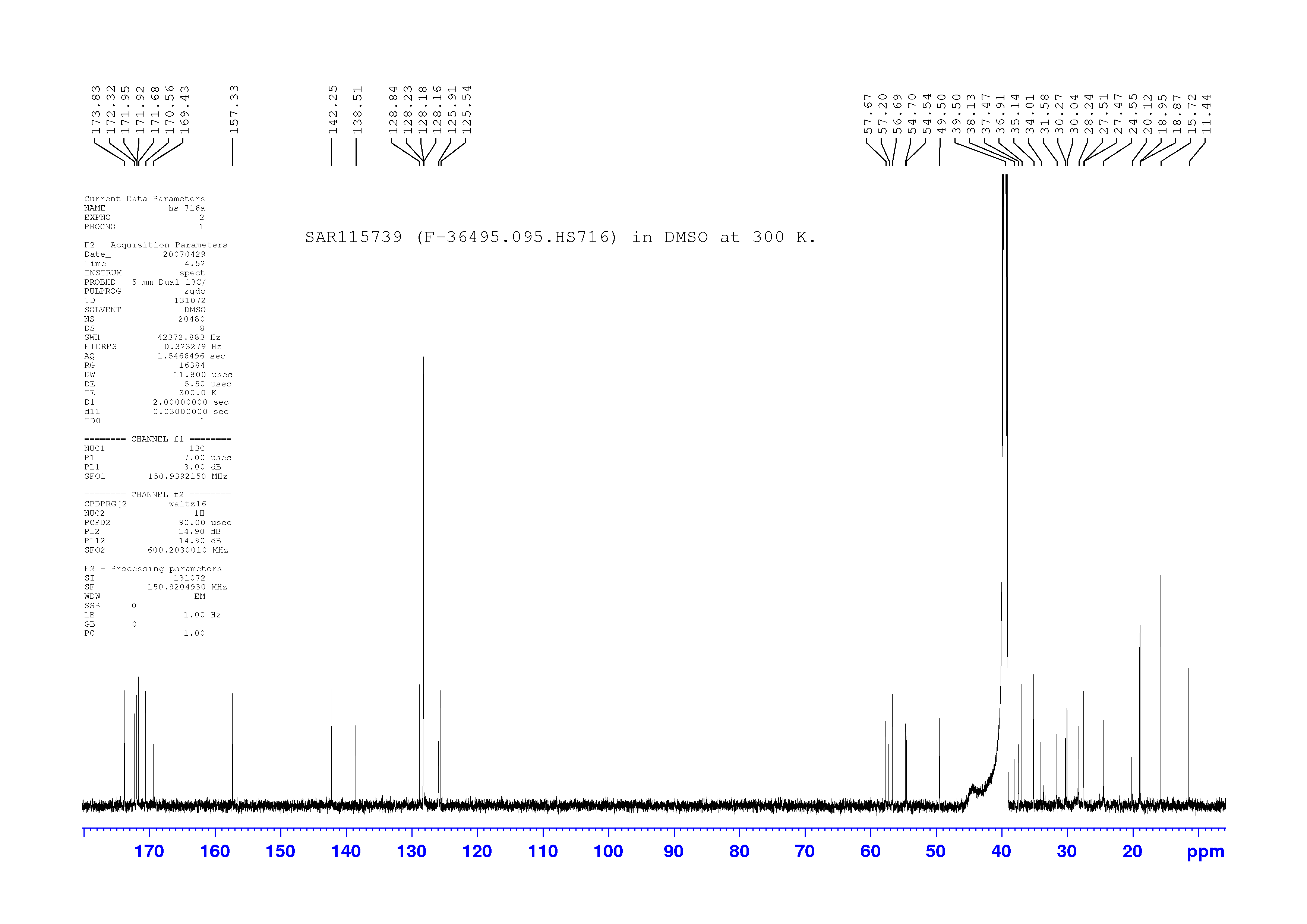


Fig. : ^13^C-spectrum of **6** in DMSO at 300 K.

Table 1: Chemical shifts of **6** DMSO at 300 K.

|  | ^1^H | ^13^C |
| --- | --- | --- |
| Phe-1 NH | 9.00 | - |
| α | 4.42 | 54.71 |
| β | 3.38/2.77 | 37.47 |
| γ | - | 138.51 |
| δ | 7.05 | 128.85 |
| ε | 7.19 | 128.19 |
| ζ | 7.14 | 125.91 |
| C’ | - | 170.56 |
| N-Me-Asn-2 NMe | 1.78 | 27.47 |
| α | 5.35 | 57.20 |
| β | 2.50/2.35 | 34.01 |
| γ | - | 171.68 |
| NH2 | 7.39/6.89 | - |
| C’ | - | 169.43 |
| APPA-3 NH | 8.66 | - |
| α | 4.74 | 49.50 |
| β | 1.81/1.59 | 30.27 |
| Β’ | 1.76/1.68 | 27.51 |
| Β’’ | 2.62/2.54 | 35.14 |
| γ | - | 142.26 |
| δ | 7.22 | 128.23 |
| ε | 7.28 | 128.16 |
| ζ | 7.17 | 125.54 |
| C’ | - | 171.92 (a) |
| Val-4 NH | 6.77 | - |
| α | 3.91 | 57.67 |
| β | 1.92 | 30.04 |
| γ | 1.00 | 18.87 |
| γ’ | 0.90 | 18.95 |
| C’ | - | 172.32 |
| Lys-5 NH | 6.45 | - |
| α | 3.89 | 54.54 |
| β | 1.59 | 31.58 |
| γ | 1.30/1.10 | 20.12 |
| δ | 1.46/1.38 | 28.25 |
| ε | 3.62/2.76 | 38.13 |
| ζ-NH | 7.59 | - |
| C’ | - | 171.95 (a) |
| Ile-6 NH | 6.33 | - |
| α | 4.06 | 56.69 |
| β | 1.73 | 36.92 |
| β-Me | 0.84 | 15.72 |
| γ | 1.35/1.11 | 24.55 |
| δ | 0.84 | 11.44 |
| C’ | - | 173.83 |
| 1’-C’ | - | 157.33 |

(a): maybe interchanged

APPA=2-Amino-5-phenylpentanoic acid

### Isolation of 18

Extraction of biomass of CBT599

152g of dried biomass were extracted four times with a mixture of water : methanol 1:1. All four extracts were combined to give ~5.5L of crude extract.

Solid phase extraction of CBT599

The crude extract (~5.5L) was loaded onto a column filled with ~5.8L of CHP20-P (MCI® Gel, 75-150µ, Mitsubishi Chemical Corporation) material.
A gradient with water : acetonitrile was applied (10% to 80% within 60 minutes, followed by 80% to 100% within 5 minutes, flow rate 120mL/min). Fractions were collected over 2 minutes. The fractions containing the compounds of interest were combined, freeze-dried and further purified.

Isolation of **18**

Fraction 13 from the solid phase extraction was separately purified using a Phenomenex Luna C18(2) column (dimension: 21x250mm, 10µm) equipped with a Waters X-Terra pre-column (dimension: 19x10mm, 10µm).
The compound was eluted using a gradient of ammonium acetate (50g NH_4_Ac /L H_2_O adjusted to pH 4.6 with acetic acid) : acetonitrile (20% to 40% within 45 minutes, flow rate: 60mL/min). Fractions were collected using UV-triggering (210nm) in 30mL fractions.
The separation yielded 7.4mg of **18** after freeze-drying in good purity.

**18**

UV: 204 nm
C_43_H_62_N_8_O_9_, monoisotopic molecular mass (calc.): 834.4640
Calc. [M+H^+^]: 835.4718 Da; observed [M+H^+^]: 835.4717 Da

Fig. 1 Structure of **18**


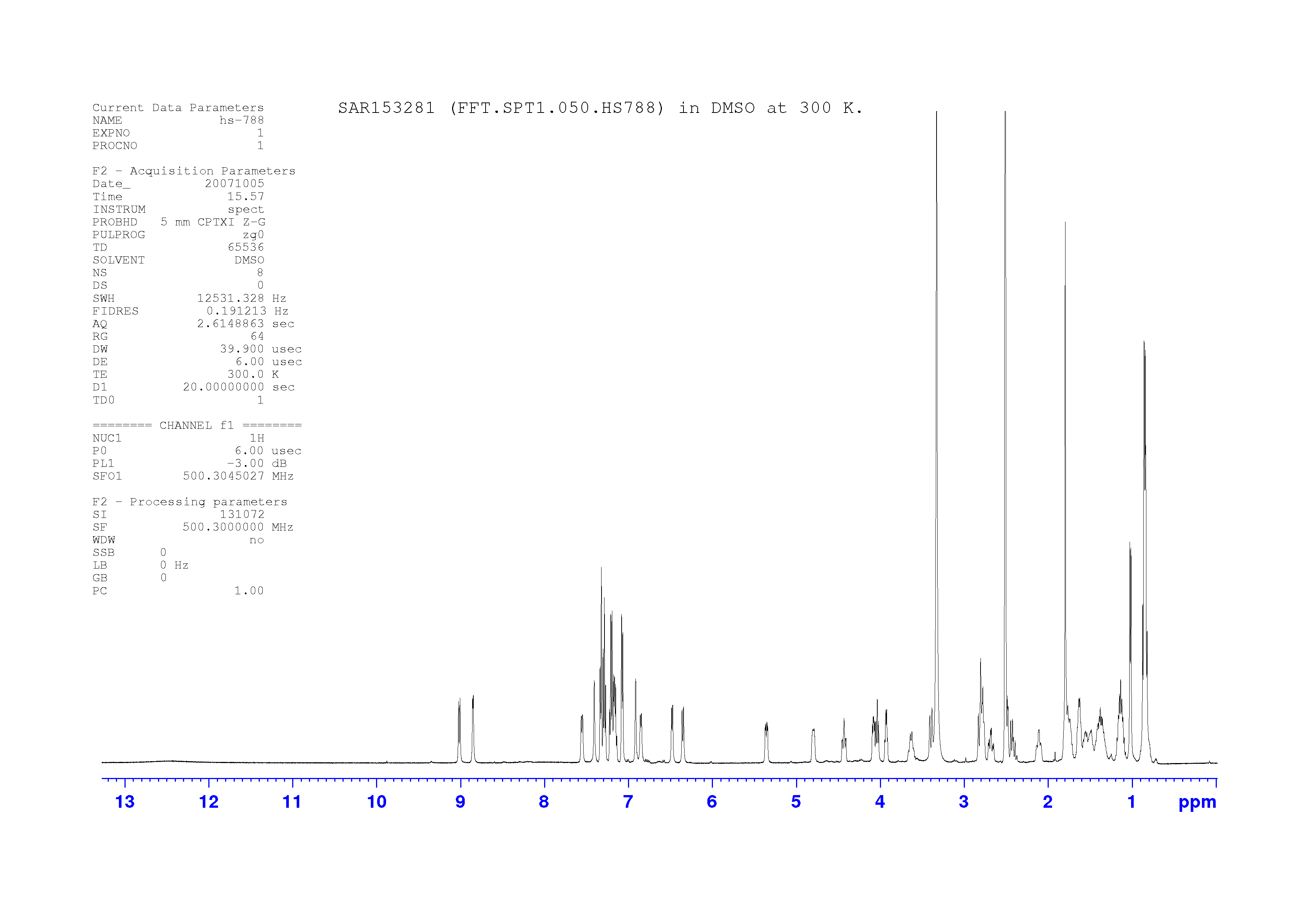


Fig. : ^1^H-spectrum of **18** in DMSO at 300 K.


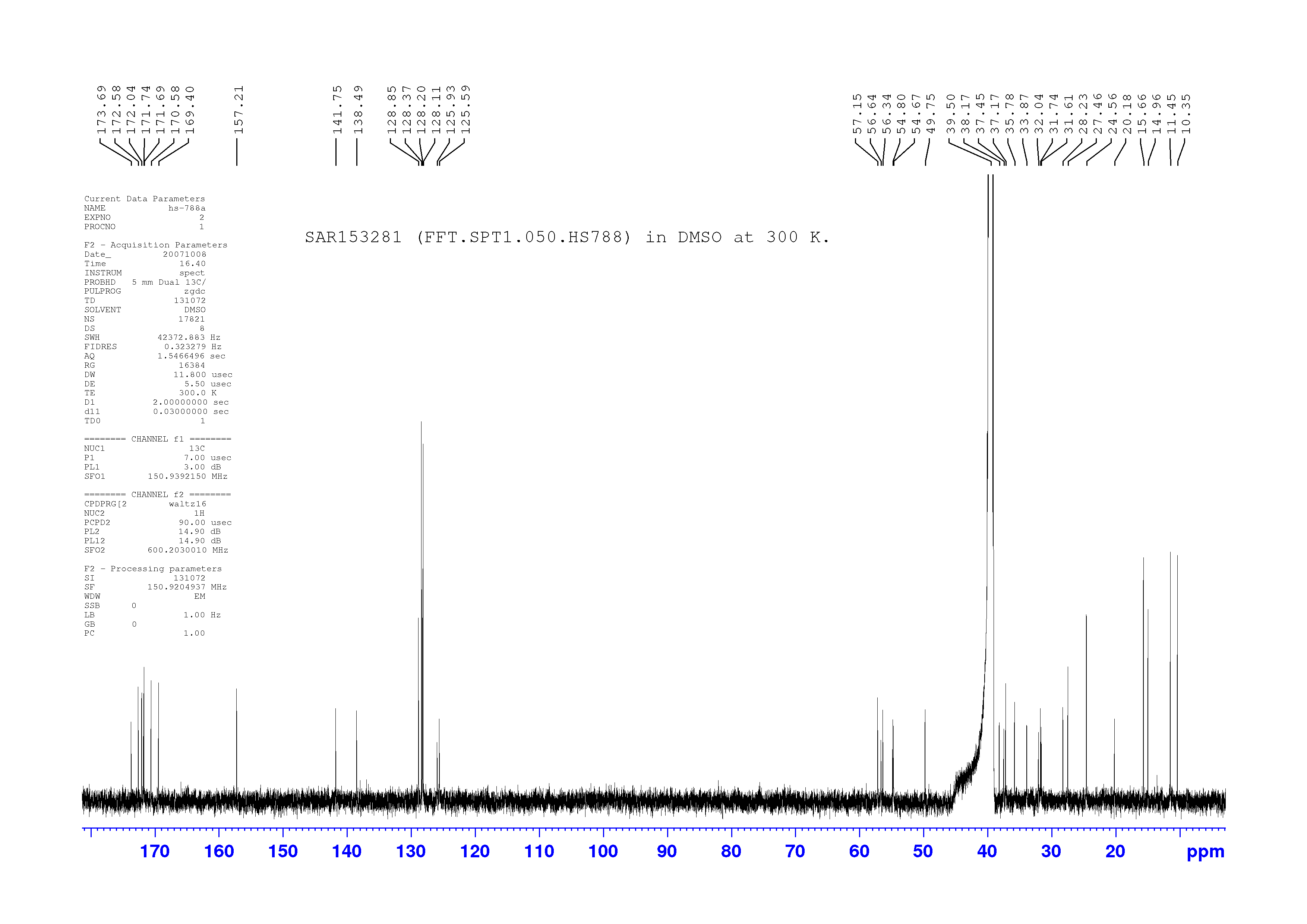


Fig. : ^13^C-spectrum of **18** in DMSO at 300 K.

Table 1: Chemical shifts of **18** in DMSO at 300 K.

|  | ^1^H | ^13^C |
| --- | --- | --- |
| Phe-1 NH | 9.00 | - |
| α | 4.42 | 54.80 |
| β | 3.39/2.79 | 37.45 |
| γ | - | 138.49 |
| δ | 7.06 | 128.85 |
| ε | 7.20 | 128.20 |
| ζ | 7.14 | 125.93 |
| C’ | - | 170.58 |
| N-Me-Asn-2 NMe | 1.78 | 27.46 |
| α | 5.35 | 57.16 |
| β | 2.49/2.41 | 33.87 |
| γ | - | 171.69 |
| NH2 | 7.40/6.90 | - |
| C’ | - | 169.40 |
| Hty-3 NH | 8.84 | - |
| α | 4.79 | 49.75 |
| β | 2.10/1.77 | 32.04 |
| homo-β | 2.80/2.67 | 31.74 |
| γ | - | 141.75 |
| δ | 7.32 | 128.37 |
| ε | 7.28 | 128.11 |
| ζ | 7.17 | 125.59 |
| C’ | - | 171.74 |
| Ile-4 NH | 6.84 | - |
| α | 4.03 | 56.34 |
| β | 1.78 | 35.78 |
| β-Me | 1.01 | 14.96 |
| γ | 1.55/1.14 | 24.58 |
| δ | 0.82 | 10.36 |
| C’ | - | 172.58 |
| Lys-5 NH | 6.47 | - |
| α | 3.92 | 54.67 |
| β | 1.62 | 31.61 |
| γ | 1.33/1.13 | 20.18 |
| δ | 1.49/1.41 | 28.23 |
| ε | 3.62/2.77 | 38.17 |
| ζ-NH | 7.54 | - |
| C’ | - | 172.05 |
| Ile-6 NH | 6.34 | - |
| α | 4.07 | 56.64 |
| β | 1.73 | 37.17 |
| β-Me | 0.84 | 15.66 |
| γ | 1.37/1.11 | 24.56 |
| δ | 0.85 | 11.46 |
| C’ | - | 173.70 |
| 1’-C’ | - | 157.21 |

1. Okumura, Hilary S.; Philmus, Benjamin; Portmann, Cyril; Hemscheidt, Thomas K. **Homotyrosine-​Containing Cyanopeptolins 880 and 960 and Anabaenopeptins 908 and 915 from Planktothrix agardhii CYA 126/8** Journal of Natural Products (2009), 72(1), 172-176. [↑](#footnote-ref-1)
2. Okumura, Hilary S.; Philmus, Benjamin; Portmann, Cyril; Hemscheidt, Thomas K. **Homotyrosine-​Containing Cyanopeptolins 880 and 960 and Anabaenopeptins 908 and 915 from Planktothrix agardhii CYA 126/8** Journal of Natural Products (2009), 72(1), 172-176. [↑](#footnote-ref-2)
3. Grach-Pogrebinsky, Olga; Carmeli, Shmuel. **Three novel anabaenopeptins from the cyanobacterium Anabaena sp.** Tetrahedron (2008), 64(44), 10233-10238. [↑](#footnote-ref-3)
4. ^13^C-chemical shifts have been taken from a ^13^C-spectrum recorded at 300 K. ^1^H-chemical shifts have been determined at 310 K due to an improved dispersion of the amide resonances at this temperature. [↑](#footnote-ref-4)
